# Risk of transmission of respiratory viruses during aerosol-generating medical procedures (AGMPs) revisited in the COVID-19 pandemic: a systematic review

**DOI:** 10.1186/s13756-022-01133-8

**Published:** 2022-08-11

**Authors:** Jenine Leal, Brenlea Farkas, Liza Mastikhina, Jordyn Flanagan, Becky Skidmore, Charleen Salmon, Devika Dixit, Stephanie Smith, Stephen Tsekrekos, Bonita Lee, Joseph Vayalumkal, Jessica Dunn, Robyn Harrison, Melody Cordoviz, Roberta Dubois, Uma Chandran, Fiona Clement, Kathryn Bush, John Conly, Oscar Larios

**Affiliations:** 1 Infection Prevention and Control, Alberta Health Services, Calgary, AB Canada; 2 Department of Community Health Sciences, University of Calgary, Calgary, AB Canada; 3 Department of Microbiology, Immunology, and Infectious Diseases, University of Calgary, Calgary, AB Canada; 4 O’Brien Institute of Public Health, University of Calgary, Calgary, AB Canada; 5 Health Technology Assessment Unit, University of Calgary, Calgary, AB Canada; 6 Department of Pathology & Laboratory Medicine, University of Calgary and Alberta Health Services, Calgary, AB Canada; 7 Department of Medicine (Infectious Diseases), University of Calgary and Alberta Health Services, Calgary, AB Canada; 8 Department of Pediatrics, University of Calgary and Alberta Health Services, Calgary, AB Canada; 9 Workplace Health & Safety, Alberta Health Services, Calgary, AB Canada; 10 Department of Medicine (Infectious Diseases), University of Alberta, Edmonton, AB Canada; 11 Department of Pediatrics (Infectious Diseases), University of Alberta, Edmonton, AB Canada; 12 Provincial Respiratory Services, Alberta Health Services, Edmonton, Canada; 13 Synder Institute for Chronic Diseases, University of Calgary and Alberta Health Services, Calgary, AB Canada; 14 Workplace Health & Safety, Alberta Health Services, Edmonton, Canada

**Keywords:** Aerosol-generating procedures, Acute viral respiratory infection, Healthcare worker, COVID-19, Transmission, SARS

## Abstract

**Background:**

In many jurisdictions healthcare workers (HCWs) are using respirators for aerosol-generating medical procedures (AGMPs) performed on adult and pediatric populations with all suspect/confirmed viral respiratory infections (VRIs). This systematic review assessed the risk of VRIs to HCWs in the presence of AGMPs, the role respirators versus medical/surgical masks have on reducing that risk, and if the risk to HCWs during AGMPs differed when caring for adult or pediatric patient populations.

**Main text:**

We searched MEDLINE, EMBASE, Cochrane Central, Cochrane SR, CINAHL, COVID-19 specific resources, and MedRxiv for English and French articles from database inception to September 9, 2021. Independent reviewers screened abstracts using pre-defined criteria, reviewed full-text articles, selected relevant studies, abstracted data, and conducted quality assessments of all studies using the ROBINS-I risk of bias tool. Disagreements were resolved by consensus. Thirty-eight studies were included; 23 studies on COVID-19, 10 on SARS, and 5 on MERS/ influenza/other respiratory viruses. Two of the 16 studies which assessed associations found that HCWs were 1.7 to 2.5 times more likely to contract COVID-19 after exposure to AGMPs vs. not exposed to AGMPs. Eight studies reported statistically significant associations for nine specific AGMPs and transmission of SARS to HCWS. Intubation was consistently associated with an increased risk of SARS. HCWs were more likely (OR 2.05, 95% CI 1.2–3.4) to contract human coronaviruses when exposed to an AGMP in one study. There were no reported associations between AGMP exposure and transmission of influenza or in a single study on MERS. There was limited evidence supporting the use of a respirator over a medical/surgical mask during an AGMP to reduce the risk of viral transmission. One study described outcomes of HCWs exposed to a pediatric patient during intubation.

**Conclusion:**

Exposure to an AGMP may increase the risk of transmission of COVID-19, SARS, and human coronaviruses to HCWs, however the evidence base is heterogenous and prone to confounding, particularly related to COVID-19. There continues to be a significant research gap in the epidemiology of the risk of VRIs among HCWs during AGMPs, particularly for pediatric patients. Further evidence is needed regarding what constitutes an AGMP.

**Supplementary Information:**

The online version contains supplementary material available at 10.1186/s13756-022-01133-8.

## Introduction

The emergence and ongoing worldwide spread of Severe Acute Respiratory Syndrome Coronavirus-2 (SARS-CoV-2) causing the Coronavirus Disease 2019 (COVID-19) pandemic has led to much debate regarding the predominant routes of transmission of SARS-CoV-2 [1]. Over the course of the pandemic, the science of the modes of transmission of SARS-CoV-2 has evolved with increasing recognition that transmission occurs through multiple modes, including inoculation or inhalation of liquid particles of varying sizes from larger respiratory droplets to small aerosols which may come into contact with the mucous membranes of the respiratory tract or eyes as well as direct contact. An important emerging concept is that the respiratory particles represent a continuum along a spectrum of sizes from fine aerosols to large droplets [1–6]. The relative contribution of each transmission mode and how it may vary by setting and circumstance, however, is not well delineated and requires additional scientific study [1, 5–7]. In healthcare settings, transmission through the aerosolization of SARS-CoV-2, and other respiratory viral pathogens, may stem from specific medical procedures termed aerosol generating medical procedures (AGMPs) [5]. No universally agreed upon list or definition of AGMPs has been established for healthcare settings, and during the COVID-19 pandemic an increasing number of procedures have been considered by professional societies and organizations based on expert opinion [3, 4, 8–13]. The World Health Organization (WHO) and Centers for Disease Control and Prevention (CDC) consider open suctioning of airways, sputum induction, cardiopulmonary resuscitation, endotracheal intubation and extubation, non-invasive ventilation, bronchoscopy, and manual ventilation as AGMPs based on previous literature reviews [3, 4, 14]. These procedures were considered to generate higher concentrations of infectious respiratory aerosols based on epidemiological evidence, predominantly from case–control and retrospective cohort studies done on the transmission of SARS-CoV-1 or based on a theoretical risk of viral aerosolization and therefore increased risk of infection among healthcare workers (HCWs) [4, 5]. As a result, international infection prevention and control guidelines have recommended personal protective equipment (PPE) include a high level of respiratory protection (i.e., N95 or equivalent particulate respirators) among HCWs when performing AGMPs on adult patients with suspected or confirmed acute viral respiratory infections (VRI) [3, 9]. However, this recommendation varies across Canadian pediatric facilities.

An informal survey of ten Canadian pediatric hospital infection control programs in July 2021 showed that eight hospitals were using N95 respirators when performing AGMPs on patients with suspected or confirmed SARS-CoV-2 and MERS but not with other respiratory viruses, and two hospitals were also using N95 respirators with confirmed or suspected influenza (Lee BE, personal communication).

The COVID-19 pandemic resulted in the implementation of several public health and social measures (PHSMs) to mitigate spread, which have likely contributed to the significant reduction of seasonal respiratory viruses in Canada [15], US [16, 17], and Europe [18, 19]. With the increasing distribution of COVID-19 vaccines and as public health restrictions are lifted across jurisdictions, it is hypothesized that there will be a resurgence of some seasonal respiratory viruses, potentially during the troughs of the pandemic waves [17, 19, 20]. This “rebound effect” of VRIs [21–23] has already been reported in many countries, and is thought to be driven by the immunological deficit populations may have due to COVID-19 PHSMs. Many jurisdictions are considering the use of particulate respirators for AGMPs performed on both adult and pediatric populations with all suspect or confirmed VRI. Therefore, the aim of this systematic review was to understand (1) the risk of respiratory viral transmission to HCWs in the presence of AGMPs, including relevant studies from the current COVID-19 pandemic; (2) what role N95 respirators versus medical/surgical masks have on reducing that risk; and (3) whether the overall risk to HCWs during AGMPs differed when caring for adult or pediatric patient populations.

## Methods

### Eligibility criteria

The population of interest were HCWs working in patient-facing roles at risk of contracting an acute VRI from the patient (Table 1). The exposure of interest was performing, assisting, or being present during an AGMP. No restrictions were placed on PPE during AGMPs. The comparator of interest was not performing, assisting, or being present during an AGMP. Studies had to report the risk of transmission of a VRI to HCWs. VRI had to be confirmed by laboratory test (i.e., test for the presence of virus, viral antigens, or antibodies) or recognized case definitions (e.g., WHO criteria for SARS-CoV-1). All comparative studies were eligible for inclusion. Exclusion criteria were studies that did not report transmission of VRI to HCW (e.g. patient-to-patient transmission); no distinction between AGMP and non-AGMP groups; transmission of VRI that was not confirmed by lab test or established case definitions (e.g., reporting influenza-like symptoms); non-comparative study designs; editorials, case reports, and commentaries; non-English or French publications.

**Table 1:** Inclusion and exclusion criteria

Inclusion criteria	Exclusion criteria
• HCWs caring for patients with acute viral respiratory infections^a^ • Provision of care to patients undergoing aerosol-generating procedures (See Additional file 2 for full list of included AGMPs) • A comparator that was exposure to non-aerosol generating medical procedures • Transmission of confirmed VRIs from patients to HCWs • Randomized controlled trials (RCTs), and non-randomized comparative studies	• Population of interest was not HCW • Respiratory infections due to bacterial pathogens • Measure of transmission was not from patient to HCW • HCW did not have confirmed VRI based on established case definitions (e.g., WHO criteria for SARS-COV-1) or laboratory confirmation • Comparator was not exposure to non-AGMPs • Editorials, commentaries, reviews, conference abstracts

^a^Full list of respiratory viruses or viral infections: SARS-CoV-2 (COVID-19), SARS-CoV-1 (SARS), MERS-CoV (MERS), influenza A/B/C, respiratory syncytial virus, parainfluenza, human metapneumovirus

### Information sources & search strategy

A systematic review of the literature was conducted in accordance with the Cochrane Handbook for Systematic Reviews of Interventions [24] and reported in accordance with PRISMA 2020 reporting guidelines [25]. An experienced medical information specialist developed and tested the search strategies through an iterative process in consultation with the review team. The MEDLINE strategy was peer reviewed by another senior information specialist prior to execution using the PRESS Checklist [26].

Using the multifile option and deduplication tool available on the OVID platform, we searched Ovid MEDLINE®, including Epub Ahead of Print, In-Process & Other Non-Indexed Citations, Embase, EBM Reviews—Cochrane Central Register of Controlled Trials, and EBM Reviews—Cochrane Database of Systematic Reviews. We also searched CINAHL on Ebsco. The Ovid searches were performed on September 8, 2021, and the CINAHL search on September 9, 2021 from the inception of these databases to these dates.

We also searched COVID-specific resources (Cochrane COVID-19 Study Register, Covid-END, Living Overviews of Evidence (L-OVE), UNCOVER, ClinicalTrials.gov – Covid-19 resources, and the WHO Covid-19 Database) and the MedRxiv preprint server on September 9, 2021. This search was supplemented by hand-searching the reference lists of systematic literature reviews and recent health technology assessments, to ensure that all relevant studies were captured. Specific details regarding the strategies appear in Additional file 1.

The strategies utilized a combination of controlled vocabulary (e.g., “Aerosols”, “Health Personnel”, “Infectious Disease Transmission”, “Patient-to-Professional”) and keywords (e.g., “nebulize”, “health care worker”, “viral transmission”). Vocabulary and syntax were adjusted across the databases. The list of AGMPs included in the search was derived from the WHO, CDC lists based on previous systematic reviews and those included by expert opinion on the Alberta Health Services AGMP Guidance Tool, as of August 26, 2021 [13] (see Additional file 2). No language or date limits were applied in the search strategy, but animal-only records and conference abstracts were removed where possible. Results were downloaded and duplicates removed using EndNote version 9.3.3 (Clarivate Analytics) and uploaded to Microsoft Word.

This systematic review is registered in the International Prospective Register of Systematic Reviews (PROSPERO-CRD42021282323).

### Selection process

Abstracts identified through database searching were screened in duplicate; all abstracts included at this stage by either reviewer proceeded to full-text review. Full-text publications were screened in duplicate. Any discrepancies between reviewers’ inclusions were resolved through discussion between reviewers. A third-party adjudication process was used in the event of non-resolution between reviewers.

### Data collection process

For all included studies, year of publication, country, study design and setting, patient characteristic (e.g., age and disease status), sample characteristics (e.g., HCW characteristics), type of AGMP, type and diagnosis of VRI, type of PPE, and all data on transmission of VRI were extracted by a single reviewer and verified by a second reviewer. Discrepancies between reviewers during data extraction were resolved through consensus. A third-party adjudication process was used in the event of non-resolution between reviewers.

### Study risk of bias assessment

The quality of each included study was assessed using the Risk of Bias in Non-randomized Studies for Interventions tool (ROBINS-I) [27]. Each study was appraised using seven criteria broadly assessing bias due to confounding, selection of participants, classification of intervention/exposure, deviations from intended intervention/exposure, missing data, measurement of outcomes, and selection of reported results. Quality assessment was completed in duplicate with discrepancies resolved through discussion. A third-party adjudication process was used in the event of non-resolution between reviewers. Studies were not excluded based on quality assessment.

### Synthesis methods

Meta-analysis to pool unadjusted or adjusted odds ratios for studies that reported a statistical comparison between AGMPs and transmission of VRI was considered only if there were no heterogeneity between studies. Studies that reported only count data (e.g., number of events) where no statistical comparisons were made were summarized narratively and are not included in data exhibits. Crude or unadjusted odds ratios were not calculated manually for studies that reported only count data, as this would not take into consideration of any missing data (e.g., the true sample size was unknown). Data were reported and summarized narratively and in tabular form. Data were stratified by VRI type (e.g., COVID-19, SARS, MERS, Influenza, or other), and then by type of AGMP performed. Results pertaining to the secondary research question regarding the use of particulate respirators versus other PPE during AGMPs were narratively reported.

## Results

### Study selection

**Fig. 1:**
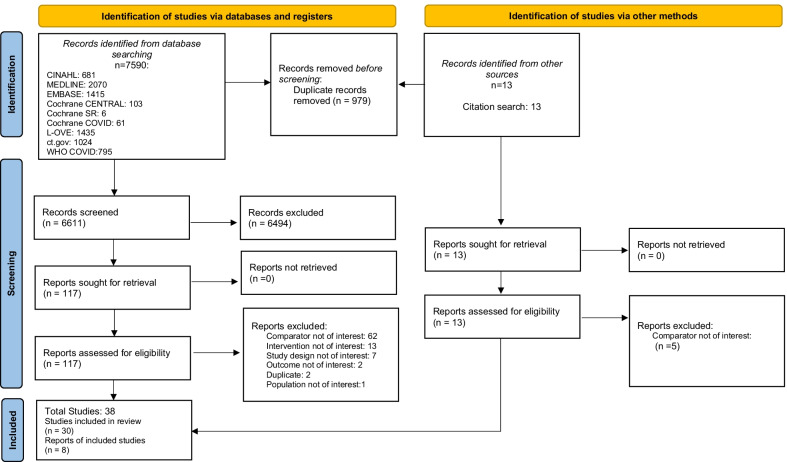
PRISMA flowchart of included studies

The search strategy yielded 6624 unique citations, 6094 of which were excluded following abstract review (Fig. 1). One-hundred twenty-nine studies proceeded to full-text review where an additional 92 studies were excluded for the following reasons: comparator was not non-AGMP (n = 67); exposure was not an AGMP (n = 13); study design was not comparative (n = 7); outcome was not transmission of VRI (n = 2); duplicate study (n = 2); or the population was not HCWs (n = 1) (see Additional file 3). After full-text review, 38 studies met inclusion criteria, and are included in this evidence synthesis. Study characteristics of included studies are presented in Table 2.

**Table 2:** Study characteristics of included studies (n = 38)

Author (Year), Country	Design/setting	Recruitment period	HCW population (n; role)	Patient population (age; disease status; symptoms; or stage of illness)	Aerosol-generating medical procedures (as described in the studies)	Viral respiratory infection/ laboratory tests	Outcome Data analysis presentation
*Alraddadi (2016), Saudi Arabia [54]	Retrospective cohort/Single Centre – Medical intensive care unit, emergency department, neurology unit	May to June 2014	n = 283; radiology technicians, nurses, respiratory therapists, physicians, clerical staff, patient transporters	NR; MERS-positive; NR; NR	Specific AGMPs and AGMPs Combined: Airway suctioning, bronchoscopy, CPR, intubation, extubation, non-invasive ventilation, manual ventilation, HFOV, nebulizer treatments, insertion of nasogastric tubes, insertion of peripheral line insertion of central venous line, chest physiotherapy, manipulating face mask or tubing, tracheostomy care, chest tube insertion/removal	MERS/ELISA	Number of events, stratified RR by mask type
Brown (2021), USA [43]	Retrospective cohort/Multicentre – Emergency medical services	February 16 to July 31, 2020	n = 1592; EMS providers	Adults; COVID-19-positive; fever, cough, shortness of breath, sore throat, nasal congestion, gastrointestinal symptoms, body aches, altered mental status, fatigue/weakness, headache, chest pain; NR	AGMPs Combined: Endotracheal intubation, supraglottic airway insertion, bag-valve-mask (BVM) ventilation (with or without chest compressions for CPR), continuous positive airway pressure nonrebreather mask oxygen (high-flow oxygen 15L/min), nebulizer medication therapy, CPR	COVID-19/ RT-PCR	Number of events, unadjusted IRR
Caglayan (2021), Turkey [34]	Cross-sectional/Multicentre -thoracic societies	March 11 to May 15, 2020	n = 15; physicians	NR; confirmed or suspected COVID-19-positive; fever and other COVID-19 symptoms; NR	Specific AGMP: Bronchoscopy	COVID-19/ Self-reported (type of diagnostic test not reported)	Number of events, statistical comparison of groups (i.e. p-values)
Celebi (2020), Turkey [29]	Case–control/Single Centre – all units	March 20 and May 20, 2020	n** = **181; physicians, nurses, cleaning personnel, and other occupations	NR; suspected or confirmed COVID-19-positive; NR; NR	Specific AGMPs*:* CPR, intubation, bronchoscopy, endoscopy, mechanical ventilation	COVID-19/ RT-PCR	Number of events, statistical comparison of groups (i.e. p-values)
*Chano (2021), Japan [46]	Prospective cross-sectional/Multicentre – COVID-19 designated hospitals	February 15 to February 26, 2021	n = 1237; doctors, nurses, office workers, and others	NR	Not specifically identified in the paper but list of AGMPs combined received from personal communication with the author: Tracheal suction, tracheal intubation, extubation, bronchoscopy, nebulizer therapy	COVID-19/ Serology	Number of events, unadjusted OR
Chatterjee (2020), India [28]	Case–control/Multicentre – all units	April to May 2020	n = 751; doctor, nurses, housekeeping, security, lab techs, other	NR	Specific AGMPs: Respiratory suctioning, endotracheal intubation, nasopharyngeal swab collection	COVID-19/ RT-PCR	Number of events, unadjusted and adjusted OR
Chen (2009), China [65]	Retrospective/Multicentre – all units involved in the care of SARS patients	2003	n = 758; doctors, nurses, health attendant, lab technician, other	NR; suspected or confirmed SARS-positive; NR; ‘caring for super spreading patient(s)’	Specific AGMPs; Tracheal intubation, tracheotomy	SARS/ ELISA	Unadjusted and adjusted OR
Cheng (2015), Hong Kong [53]	Retrospective cohort/Single Centre – adult ICU and general medical ward	April 1, 2013 to May 31, 2014	n = 70; NR	One, 36-year old, female with verified influenza A H7N9; 6-days after onset of symptoms (fever, cough, sputum production, sore throat, dyspnoea); one 65-year old male with verified influenza A H7N9; 4-days after onset of community-acquired pneumonia	AGMPs Combined: Endotracheal intubation, CPR, bronchoscopy, open suction of respiratory tract, sputum induction, use of nebulizer therapy, non-invasive positive pressure ventilation, high-frequency oscillatory ventilation	Influenza A H7N9/ Serology	Number of events, statistical comparison of groups (i.e. p-values)
Cummings (2020), USA [51]	Post-hoc analysis of RCT/Multicentre – outpatient clinics, ED	2011 to 2016	n = 2614; NR	NR; suspected respiratory illness; NR; NR	Specific AGMPs and AGMPs Combined: Respiratory/airway suctioning, intubation, nasopharyngeal aspiration, nebulizer treatment-	Coronavirus strains HKU1, OC43, NL63, 229E/ RT-PCR	Number of events, adjusted and unadjusted OR
Dimcheff (2020), USA [30]	Cross-sectional/Single Centre – all units	June 8 to July 8, 2020	n = 1476; advanced care practitioner (e.g., physician, dentist, NP, PA, nurse anesthetist), administrative, clinical support, nursing	Veterans; NR; NR; NR	Not specifically identified	COVID-19/ Serology	Number of events, unadjusted OR
Firew (2020), USA [31]	Cross-sectional survey/Multicentre – Emergency medicine, ICU, inpatient hospital, pre-hospital, outpatient, long-term care facility/nursing home, other	May 2020	n** = **2040; physicians, nurses, EMTs, resident physicians or fellows, physician assistants and other HCW	NR	AGMPs Combined: Respiratory/airway suctioning, intubation, nasopharyngeal aspiration, nebulizer treatment-	COVID-19/ PCR and antibody	Number of events, unadjusted PR
Fowler (2004), Canada [64]	Retrospective cohort/Single Centre—ICU	April 1 to April 22, 2003	n = 122; physicians, registered nurses, respiratory therapist treating SARS patients	NR; SARS-positive treated with ventilator support; NR; NR	Specific AGMPs: Endotracheal intubation, high-airflow, noninvasive positive-pressure ventilation (NIPPV); high-frequency oscillatory (HFO) ventilation	SARS/ PCR or serology	Number of events, stratified and adjusted RR
Ha (2004), Vietnam [61]	Prospective cohort/Single Centre – all units	May 12 to 14 2003	n = 62; Physicians, nurses, nonclinical staff (housekeepers, clerks, elevator operators, laboratory technicians, and guards)	NR; SARS-positive; NR; NR	Specific AGMPs: Non-invasive positive pressure ventilation -, nebulizer medication	SARS/ Serology	Number of events only
*Haller (2021), Switzerland[49]	Prospective observational cohort/Multicentre – all units	June 22nd to August 15^th^, 2020, data were analysed up to March 9th 2021	n = 3259; primarily nurses and physicians	NR	AGMPs Combined*:* Bronchoscopies, intubation/extubation, gastroscopy, transesophageal echocardiography, reanimation, non-invasive ventilation, and suction of tracheal secretions	COVID-19/ RT-PCR, seroconversion	Number of events, unadjusted and adjusted OR, adjusted HR
Heinzerling (2020), USA[48]	Cohort/Single Centre	February 15 to February 19, 2020	n = 43; RN, RT, phlebotomist, certified nursing assistant, physician, environmental services worker, nutritionist, pharmacist, other	One index patient, assumed adult; COVID-19-positive; NR; NR	Specific AGMPs and AGMPs Combined: Airway suctioning, noninvasive positive pressure ventilation including BiPAP, manual ventilation, nebulizer treatments, breaking the ventilator circuit, sputum induction, intubation, bronchoscopy	COVID-19/ RT-PCR	Number of events and statistical comparison of groups (i.e. p-values)
*Lai (2020), China[50]	Cross-sectional survey/Multicentre – all units	February 11 to February 15, 2020	n = 325; nurses, doctors, technicians, security and cleaning staffs, and financial staffs	NR; COVID-19 confirmed or suspected; NR; NR	Not specifically identified	COVID-19/ nucleic acid test or clinical diagnosis	Number of events and statistical comparison of groups (i.e. p-values)
Langella (2021), USA[34]	Ecological/Single Centre – emergency dental clinic	March 30 to May 8, 2020	n = 267; dental care providers	Adult and pediatric; NR; NR	AGMPs Combined*:* High or low-speed dental handpieces, rotary instruments, ultrasonic scalers, air water syringes in a biologic environment (i.e. intraorally) including manipulation of gingival tissue, procedures likely to produce droplets at operative site, including extractions	COVID-19/ NR	Number of events
*Lentz (2020), USA[32]	Case–control/Multicentre – international healthcare delivery settings	April 20 to May 5, 2020	n = 1130; clinician, nurse, RT, other	NR; laboratory confirmed or persons under investigation for COVID-19; NR; NR	Specific AGMPs and AGMPs Combined*:* Intubation, extubation, open respiratory suctioning, bronchoscopy, nebulizer use, non-invasive positive pressure ventilation (NIPPV), tracheotomy, cardiopulmonary resuscitation	COVID-19/ RT-PCR	Unadjusted and adjusted OR
Liu (2009), China [62]	Retrospective Case control; Single Centre – all units	March 5 to May 17, 2003	n = 477; NR	NR; SARS-positive; NR; NR	Specific AGMPs: Chest compression, intubation, pulmonary lavage, respiratory secretion, sputum	SARS/ Serology (ELISA)	Number of events, unadjusted and adjusted OR
Loeb (2004), Canada [63]	Retrospective cohort/Single Centre – critical care units	March 8 to March 21, 2003	n = 32; nurses	Three adult index patients; suspected SARS; NR; nR	Specific AGMPs: Suctioning after intubation, suctioning before intubation, endotracheal aspirate, bronchoscopy, CPR, intubation, manual ventilation, nebulizer treatment, insertion of a nasogastric tube, manipulation of BiPAP mask, manipulation of oxygen mask, moth or dental care, nasopharyngeal swab obtained	SARS/ Serology	Number of events, unadjusted RR
Lormans (2021), Belgium [36]	Cohort/Single Centre – COVID-19 and non-COVID-19 ICU	April to May 2020	n = 142; nurses	NR	Specific AGMPs and AGMPs Combined: Broncho-alveolar lavage (more than 5 times), any assistance with either intubation or broncho-alveolar lavage, Intubation (more than once)	COVID-19/ RT-PCR	Unadjusted OR
*Lucar (2020), USA[41]	Retrospective cohort/Single Centre – Operating room	NR	n = 11; anesthesiologist, certified registered nurse anesthetist, circulator, orthopedic surgeon, orthopedic resident, scrub technician	One 17-year-old, asymptomatic index patient; COVID-19-positive	AGMPs Combined: Extubation, intubation	COVID-19/ RT-PCR	Number of events
Macintyre (2014), China [56]	Prospective cohort/Multicentre – emergency and respiratory wards	December 1, 2008 to January 15, 2009	n = 481; doctors, nurses, other	NR	AGMPs Combined*:* Provision of nebulizer medications, suctioning, intubation, aerosol-generating procedures and chest physiotherapy	adenoviruses, human meta pneumovirus, coronaviruses, parainfluenza, influenza, respiratory syncytial virus, rhinovirus A/B; *Streptococcus pneumoniae, Mycoplasma pneumoniae, B. pertussis, Legionella spp, Chlamydophila and Haemophilus* influenza type B./ based on presence of symptoms	Number of events, unadjusted and adjusted RR
Nakagama (2021), Japan [45]	Prospective cohort/Single Centre – all units	June 30th to July 2nd 2020	n = 414; NR	NR	Specific AGMPs and AGMPs Combined: Airway suctioning, bronchoscopy, CPR, endotracheal intubation/extubation, bag mask ventilation, non-invasive ventilation, nebulizer administration, sputum induction, oxygen supplementation via tracheostomy, tracheotomy	COVID-19/ RT-PCR or serology	Number of events, unadjusted RR
Ogawa (2020), Japan [33]	Cohort/Single Centre – ICU, general ward	NR	n = 15; doctors, nurses, speech-language-therapist, and physical therapists	One 81-year-old female index patient, COVID-19 positive; 10 days from symptom onset	AGMPs Combined*:* Ventilation, tracheotomy	COVID-19/ IgG antibody blood test, RT-PCR test	Number of events
**Paris (2021), France **[44]	Cross-sectional survey/Single Centre – all units	May, 29th and July, 10th 2020	n = 3234; nurses/midwives, students, residents, medical staff, administrative staff, cleaners	NR	Specific AGMPs and AGMPs Combined: Nasopharyngeal sampling, ear, nose and throat examinations, upper respiratory tract, nasogastric tube insertion, aerosol generating procedures (not specified but referred to as actions on upper respiratory tract)	COVID-19/ Serology (ELISA)	Number of events and statistical comparison of groups (i.e. p-values); adjusted OR
Pei (2006), China [60]	Case–Control/Multicentre – all units	2002–2003 SARS outbreak; questionnaire carried out in April-June 2004	n = 443; doctors, nurses, nursing staff, workers, technicians, administrators, others	NR	Specific AGMPs: Tracheal intubation	SARS/ Serology	Number of events, unadjusted and adjusted OR
Pérez-García (2020), Spain[47]	Cross-sectional/Single Centre – all units	March 5, 2020 to May 30, 2020	n = 2963; medical staff, nurses, technical specialists, auxiliary nursing-care technician, hospital porter, resident physician, kitchen, administrative staff, others	NR	Not specifically described	COVID-19/ RT-PCR and/or serology	Unadjusted and adjusted OR
*Raboud (2010), Canada [55]	Retrospective cohort/Multicentre	2003	n = 624; NR	Adults; SARS-positive requiring intubation; days of illness reported	Specific AGMPs; Suctioning after intubation, suctioning before intubation, bronchoscopy, CPR, defibrillation, intubation, manual ventilation after and before intubation, non-invasive ventilation, mechanical ventilation, nebulizer treatment, chest physiotherapy, insertion of NG tube, manipulation of oxygen mask, high flow oxygen (in room), collection of sputum sample, suction – sputum, bronchoscopy, tracheal intubation, tracheal tube removal, laryngoscope, tracheotomy	SARS/ RT-PCR	Number of events, unadjusted and adjusted OR
Ran (2020), China [42]	Retrospective Cohort Study/Single Centre – high risk and low risk units	The follow-up was ended on Jan 28 2020	n = 72; nurses and clinicians	NR	Specific AGMPs and AGMPs Combined: High exposure operation (medical or surgical procedures that generate respiratory aerosols including tracheal intubation, tracheotomy, tracheal tube removal, CPR, sputum suction, fiber bronchoscopy, laryngoscope)	COVID-19/ RT-PCR	Number of events, unadjusted RR
Rosano (2021), Italy [38]	Cohort/Single Centre – ICU	February 20 to May 5, 2020	n = 145; physicians and nurses	NR; COVID-19-positive; NR; NR	Specific AGMPs: Tracheotomy	COVID-19/ RT-PCR or antibody test	Number of events and statistical comparison of groups (i.e. p-values)
Rzepliński (2021), Poland [37]	Cross-sectional/Single Centre – All units	April 21 to July 20, 2020	n = 1572; NR	NR	Not specifically described	COVID-19/ Serology and RT-PCR	Number of events and statistical comparison of groups (i.e. p-values)
Scales (2003), Canada [59]	Retrospective cohort/Single Centre—ICU	2003	n = 69; quarantined HCWs	One 74-year-old, signs and symptoms of community-acquired pneumonia; SARS-positive	Specific AGMPs: Intubation, non-invasive positive-pressure ventilation (present during procedure), contact with mucous membrane, contact with mucous membranes or respiratory secretions (performed procedure that involved contact with secretions)	SARS/ WHO criteria of SARS	Number of events
**Shah (2021), USA**[40]	Retrospective cohort/Single Centre – emergency department, inpatient, outpatient	May 13 to November 20, 2020	n = 345; RN, physician, RT, patient care assistant, housekeeping, other	NR; confirmed of suspected COVID-19-positive; NR; NR	AGMPs Combined: Tracheal intubation, non-invasive ventilation, tracheotomy, cardiopulmonary resuscitation, manual ventilation before intubation, bronchoscopy	COVID-19/ RT-PCR	Number of events and statistical comparisons of groups (i.e. p-values)
Teleman (2004), Singapore [58]	Case–control/Single Centre – all units	March 1 to March 31, 2003	n = 86; doctors, nurses, other	NR; SARS-positive; NR; NR	Specific AGMPs: Suction, intubation, oxygen administration, contact with respiratory secretions	SARS/ Serology	Number of events, unadjusted and adjusted OR
**Vitrat (2021), France **[39]	Cross-sectional/Multicentre – all units	June 27 to September 31, 2020	n = 3454; HCW, health-associated professionals, health management, support personals	NR	AGMPs Combined: Nasopharyngeal test, intubation, etc. (from survey), oral intubation, aerosolized therapy, high flow oxygen (in discussion)	COVID-19/ Serology	Unadjusted and adjusted OR
Wong (2004), Hong Kong [57]	Retrospective cohort/Single Centre – general medical ward	March 4 to March 10, 2003	n = 66; medical students	One index patient presumed adult; SARS-positive; NR; 8 days from symptom onset	Specific AGMPs: Nebulizer	SARS/ Serology	Number of events
Zhang (2013), China [52]	1:4 matched case–control study/Multicentre – high risk setting (intensive care, emergency, respiratory wards)	August 30, 2009 and January 31, 2010	n = 255; nurse, doctor, or ward clerk	NR; patient with confirmed pandemic influenza A ⁄ (H1N1); NR; NR	AGMPs Combined: Suctioning, intubation, nebulizer medications, chest physiotherapy, other aerosol generating procedures	Influenza A/H1N1/ RT-PCR	Number of events and statistical comparison of groups (i.e. p-values)

*Bolded authors also reported a stratified analysis of transmission of VRI to HCW during an AGMP when using a N95/respiratory versus other PPE. Specific AGMPs: Studies reporting transmission of VRIs by specific AGMPs; AGMPs Combined: Studies reporting transmission of VRIs by listed AGMPs combined; Not specifically described; Studies reporting transmission of VRIs by AGMPs, but AGMPs were not described in the study

*CPR* cardiopulmonary resuscitation; COVID-19 SARS CoV-2; *ELISA* enzyme-linked immunoassay; *EMT* emergency medical technician; *HCW* healthcare worker; *MERS* Middle East Respiratory Syndrome; *NP* nurse practitioner; *NR* not reported; *PA* physicians assistant; *RN* registered nurse; *RR* relative risk; *RT* respiratory therapist; *RT-PCR* real-time polymerase chain reaction; *SARS* Severe Acute Respiratory Syndrome

### Study characteristics

Overall, 23 studies reported on COVID-19, 10 on SARS, and 5 on MERS, influenza, and other respiratory viruses combined. Nearly two-thirds of the included studies were published in 2020/2021, after the onset of the COVID-19 pandemic (n = 24). Of these latter 24 studies, 23 reported on COVID-19 [28–50] and one reported on other coronaviruses (HKU1, OC43, NL63, 229E) [51]. Five studies (13%) were published between 2010 and 2019, reporting transmission of influenza (n = 2) [52, 53], MERS (n = 1) [54], SARS (n = 1) [55], and multiple viruses (e.g., adenovirus, influenza, coronaviruses, human metapneumovirus, rhinovirus, respiratory syncytial virus) (n = 1) [56]. Finally, nine studies (24%) were published between 2000 and 2009, all reporting on SARS [57–65]. Half of the studies were conducted in the United States (n = 8), China (n = 7), and Canada (n = 4). The remaining studies were conducted in Japan (n = 3); France, Hong Kong, and Turkey (n = 2 each); and Belgium, India, Italy, Poland, Saudi Arabia, Singapore, Spain, Switzerland, United Kingdom, and Vietnam (n = 1 each). The study designs most often employed were retrospective cohort (n = 12), cross-sectional (n = 9), and case–control studies (n = 7). No RCTs were included, however there was a post-hoc analysis of the participant intervention groups from the Respiratory Protection Effectiveness Clinical Trial (ResPECT) that was included [51].

Samples sizes varied from 11 [41] to 3454 [39] HCWs. Several different types of HCWs were included in the samples, such as nurses, physicians, nurse practitioners, respiratory therapists, physician assistants, and dentists. Other hospital personnel were included in some studies, including housekeeping/environmental services staff, security, pharmacists, laboratory technicians, hospital administrators, and others that may have encountered respiratory secretions, or been present in the room during an AGMP. While nine studies enrolled HCWs from healthcare settings that provided care to mixed populations, including newborns, children, and adults [29, 31, 35, 39, 40, 51–53, 56], there was only one study that described the outcomes of HCWs exposed to a pediatric (17 years old) patient with asymptomatic COVID-19 during an AGMP [41]. No other studies described HCW exposure to pediatric patients during AGMPs.

### Study risk of bias assessment

**Fig. 2:**
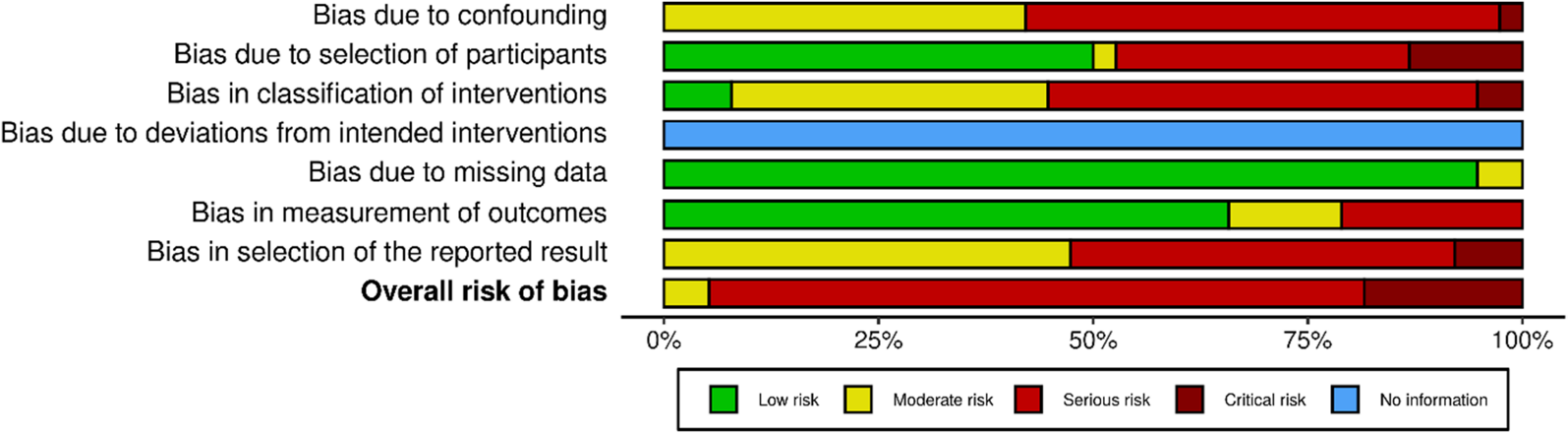
ROBINS-I summary plot

**Fig. 3:**
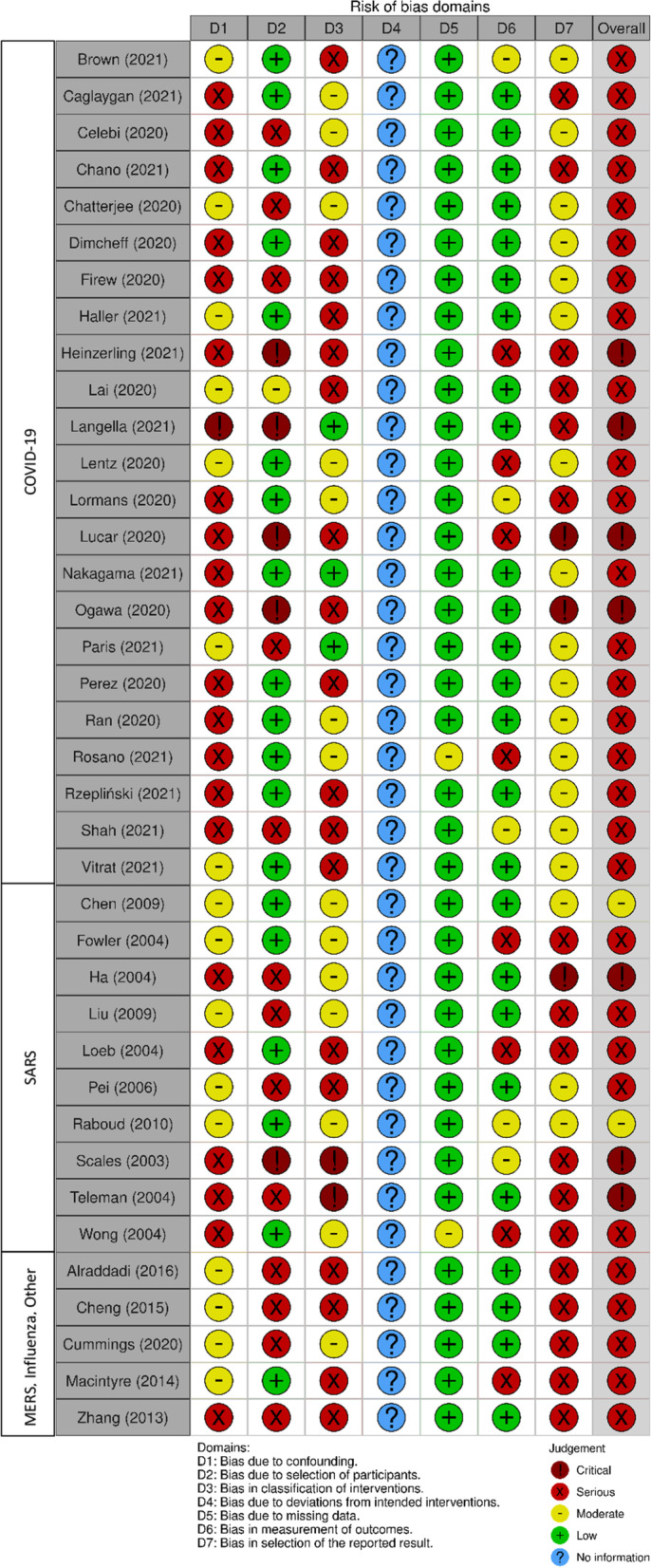
ROBINS-I traffic light plot [66]

Quality assessment was conducted using the Cochrane risk of bias for non-randomized studies (ROBINS-I) [27]. Across the 38 included studies, overall risk of bias was serious for 29 studies (at least one domain was rated as serious), critical for seven studies (at least one domain was rated as critical) [33, 35, 41, 48, 57–59] and moderate for two studies (no serious or critical ratings) [55, 65] (Figs. 2 and 3).

On risk of bias due to confounding, most studies (n = 21) were rated as serious because confounding factors were not considered or controlled. Moderate ratings for 16 studies indicated that these studies attempted to control for confounding factors in their analysis. However, there was considerable heterogeneity in the characteristics or factors considered as confounders and how each study accounted for them in multivariate analysis in these studies. It should be noted that studies that presented adjusted odds ratios/risk ratios are not reported in this review for consistency in results reporting. On risk of bias for participant selection, half of the included studies (n = 19) recruited all eligible HCWs at their site, or hospital ward of interest, therefore were rated low risk of bias. Serious (n = 13) and critical (n = 5) studies were rated as such when the participants were recruited based on the outcome of interest (e.g., case control studies that recruited COVID-19 positive, and COVID-19 negative HCWs). On bias in classification of intervention/exposure, 19 studies were rated serious as they did not define what they considered to be AGMPs, 14 studies were moderate, three were low [35, 44, 45], and two were critical [58, 59].

With respect to bias due to deviations from intended exposure, there was not enough information provided in the studies to rate on this domain. Because the studies do not comment on any known deviations from intended exposure, all studies were rated as “no information”. For bias due to missing data, most studies were low, with the exception of two studies that were moderate due to missing data for more than five percent of the sample [38, 57]. Bias in the measurement of the outcome was rated as low for 25 studies because diagnostic testing for VRI was conducted on all HCWs, regardless of exposure. Serious and moderate studies were rated as such if testing for VRI was dependent on the presence of symptoms (n = 8) [32, 38, 41, 48, 56, 57, 63, 64], or known exposure (n = 5) [36, 40, 43, 55, 59], respectively. On bias regarding selection of reported results, 18 studies reported results on all outcomes that were outlined in their data analysis plan and were rated as moderate. Seventeen studies did not report their intended outcomes in their analysis plan, therefore were rated as serious. Three studies were rated as critical because they did not report a data analysis plan [33, 41, 57]. The only two studies which achieved a moderate level of risk of bias were consistent in their findings of an increased risk of transmission of SARS in association with intubation [55, 65].

### Summary of findings

Across all pathogens, 24 studies reported transmission of VRIs during AGMPs that were not specifically identified (n = 5), or results were reported for multiple AGMPs combined (n = 19). Nineteen of these studies conducted a statistical test of significance between AGMPs that were not specified or were combined and the transmission of: COVID-19 (n = 15) [30–32, 36, 37, 39, 40, 42–45, 47–50], multiple viruses (n = 2) [51, 56], influenza (n = 2) [52, 56], MERS (n = 1) [54]. The remaining three studies reported event data only, with no statistical comparison reported [35, 41, 53]. Based on the level of heterogeneity between studies (e.g., variety of AGMPs included, degree of PPE, disease status of the patient), a meta-analysis on AGMPs which were not specified or were combined and the transmission of VRIs was not conducted.

Across all pathogens, 22 studies reported transmission of VRIs during a specific, individual AGMP. Of these, 20 studies conducted a statistical test of significance between individual AGMPs and the transmission of COVID-19 (n = 10) [12, 28, 29, 32, 34, 36, 38, 44, 45, 48], SARS (n = 8) [55, 58–60, 62–65], MERS (n = 1) [54], or multiple VRIs (n = 1) [51]. The remaining two studies reported event data only, with no statistical comparison reported [57, 61]. Based on the level of heterogeneity between studies, a meta-analysis on individual AGMPs and the transmission of VRIs was not conducted.

A more detailed synthesis stratified by VRI is presented below.

### COVID-19

#### AGMPs not specifically identified

Nineteen studies (50%) explored transmission of COVID-19 to HCWs after exposure to AGMPs which were not specifically identified (n = 5) or were combined (n = 14). Three studies reported count data only and did not conduct a statistical analysis for the association between AGMPs which were not specified or combined and transmission of COVID-19 [33, 35, 41]. For the 16 studies [30–32, 36, 37, 39, 40, 42–50] that reported results from a test of statistical significance, the results can be found in Table 3, grouped by study design. There were four statistically significant associations for transmission of COVID-19 to HCWs exposed to AGMPs in three separate studies and one study close to reaching statistical significance [45]. Of the two studies that reported statistically significant odds ratios, HCWs were 1.7 to 2.5 times more likely to contract COVID-19 after exposure to AGMP versus HCWs who were not exposed to AGMP. Only five studies accounted for confounding in adjusted measures of effect [32, 39, 44, 47, 49]. Pérez-Garcia et al. [47] found that participation in an AGMP no longer increased risk to HCWs when accounting for use of PPE, close vs. casual contact, and contact with COVID-19 patients. Paris et al. [44] found an increased risk of COVID-19 during an AGMP when adjusted for age, sex, occupation, and contact with COVID-19 patient or relative at home. Lentz et al. and Vitrat et al. [32, 39] saw no change in results when adjusting for confounders. Studies which demonstrated no associations were generally smaller or those which had no actual reported discrete sample sizes.

**Table 3:** Summary of findings from the studies that included statistical comparisons with respect to transmission of COVID-19 during AGMPs and where the AGMPs were not specified or were combined *

Author	Study design	Aerosol-generating medical procedures (as described in the studies)	Sample size	Odds ratio^a^ (95%CI)
Haller [49]	Prospective cohort	AGMPs Combined: Bronchoscopies, intubation/extubation, gastroscopy, transesophageal echocardiography, reanimation, non-invasive ventilation, and suction of tracheal secretions	AGMP: n = NR Control: n = NR	aOR: 0.96 (0.75 to 1.24)^b^ aHR: 1.18 (0.96 to 1.45)^c^
Nakagama [45]	Prospective cohort	AGMPs Combined: Airway suctioning, bronchoscopy, CPR, endotracheal intubation/extubation, bag mask ventilation, non-invasive ventilation, nebulizer administration, sputum induction, oxygen supplementation via tracheostomy, tracheotomy	AGMP: n = 212 Control: n = 202	RR: 1.59 (NR); *p* = 0.057
Brown [43]	Retrospective cohort	AGMPs Combined: Endotracheal intubation, supraglottic airway insertion, bag-valve-mask (BVM) ventilation (with or without chest compressions for CPR), continuous positive airway pressure nonrebreather mask oxygen (high-flow oxygen 15L/min), nebulizer medication therapy, CPR	AGMP: n = 705 Control: n = 1389	IRR^d^: 1.64 (0.22 to 12.26)
Ran [42]	Retrospective cohort	Specific AGMPs and AGMPs Combined: High exposure operation (medical or surgical procedures that generate respiratory aerosols including tracheal intubation, tracheotomy, tracheal tube removal, CPR, sputum suction, fiber bronchoscopy, laryngoscope)	AGMP: n = 13 Control: n = 59	0.54 (0.19 to 1.53)
Shah [40]	Retrospective cohort	AGMPs Combined: Tracheal intubation, non-invasive ventilation, tracheotomy, cardiopulmonary resuscitation, manual ventilation before intubation, bronchoscopy	AGMP: n = 225 Control: n = 120	AGMP cases: 3 Control cases: 5 *p* = 0.13
Heinzerling [48]	Cohort	Specific AGMPs and AGMPs Combined: Airway suctioning, noninvasive positive pressure ventilation including BiPAP, manual ventilation, nebulizer treatments, breaking the ventilator circuit, sputum induction, intubation, bronchoscopy	AGMP: n = 17 Control: n = 20	AGMP cases: 2 Control cases: NR *p* = 0.58
Lormans [36]	Cohort	Specific AGMPs and AGMPs Combined: Broncho-alveolar lavage (more than 5 times),any assistance with either intubation or broncho-alveolar lavage, Intubation (more than once)	AGMP: n = NR Control: n = NR	0.36 (0.1 to 1.26)
Lentz [32]	Case–control	Specific AGMPs and AGMPs Combined: Intubation, extubation, open respiratory suctioning, bronchoscopy, nebulizer use, non-invasive positive pressure ventilation (NIPPV), tracheotomy, cardiopulmonary resuscitation	AGMP: n = 321 Control: n = 634	0.90 (0.60 to 1.2)
**Chano **[46]	Cross-sectional	Not specifically identified in the paper but list of AGMPs combined received from personal communication with the author: Tracheal suction, tracheal intubation, extubation, bronchoscopy, nebulizer therapy	AGMP: n = 893 Control: n = 333	**AGMP cases: 20** **Control cases: 1** ***p* < 0.001**
Dimcheff [30]	Cross-sectional	Not specifically identified	AGMP: n = 155 Control: n = 1291	0.62 (0.25 to 1.56)
Firew [31]	Cross-sectional survey	AGMPs Combined: Respiratory/airway suctioning, intubation, nasopharyngeal aspiration, nebulizer treatment-	AGMP: n = 1080 Control: n = 774	PR: 1.09 (0.95 to 1.26)
Lai [50]	Cross-sectional survey	Not specifically identified	AGMP: n = 43 Control: n = 282	AGMP cases: 17 Control cases: 134 *p* = 0.328
Paris [44]	Cross-sectional survey	Specific AGMPs and AGMPs Combined: Nasopharyngeal sampling, ear, nose and throat examinations, upper respiratory tract, nasogastric tube insertion, aerosol generating procedures (not specified but referred to as actions on upper respiratory tract)	AGMP: n = 999 Control: n = 2071	AGMP cases: 44 Control cases: 68 *p* = 0.12
**Pérez-García **[47]	Cross-sectional	Not specifically described	AGMP: n = NR Control: n = NR	**2.54 (1.71 to 3.77)**
Rzepliński [37]	Cross-sectional	Not specifically described	AGMP: n = 332 Control: n = 1240	AGMP cases: 79 Control cases: 278 *p* = 0.6
**Vitrat **[39]	Cross-sectional	AGMPs Combined: Nasopharyngeal test, intubation, etc. (from survey), oral intubation, aerosolized therapy, high flow oxygen (in discussion)	AGMP: n = 1033 Control: n = 2421	**1.7 (1.15 to 2.49)**^**e**^ **2.03 (1.36 to 3.02)**^**f**^

*Bolded estimates represent statistically significant results (p < 0.05). Details pertaining to patients and settings and studies that only reported count data but no statistical comparisons can be found in Table 2

*AGMP* aerosol-generating medical procedure; *aOR* adjusted Odds Ratio; *aHR* adjusted Hazard Ratios; *NR* not reported; *CI* confidence interval; *RR* risk ratio; *IRR* incidence rate ratio; *PR* prevalence ratio

^a^Unadjusted Odds Ratio unless otherwise specified

^b^Adjusted Odds Ratios of SARS-CoV-2 seroconversion reported because unadjusted odds ratios could not be found

^c^Adjusted Hazard Ratios of SARS-CoV-2 PCR/rapid antigen tests reported because unadjusted odds ratios could not be found

^d^AGMP status of comparator unclear

^e^AGMP with systematic use of FFP2 compared to non-AGMP exposure

^f^AGMP without systematic use of FFP2 compared to non-AGMP exposure

### Specific AGMPs

Nine studies reported tests of statistical significance for 12 specific AGMPs and the transmission of COVID-19 including: airway suctioning (n = 5) [12, 28, 32, 45, 48], broncho-alveolar lavage (n = 1) [36], bronchoscopy (n = 4) [12, 32, 34, 48],CPR (n = 4)[12, 29, 32, 45], extubation (n = 2) [12, 32], extubation/intubation combined (n = 1) [45], intubation (n = 5) [28, 29, 32, 36, 48], manual ventilation (n = 3) [32, 45, 48], mechanical ventilation (n = 1) [29], nebulizer administration (n = 3) [32, 45, 48], oxygen administration (n = 1) [32], and tracheostomy (n = 3) [32, 38, 45] (Table 4). All reported, unadjusted associations were non-significant or had 95% CIs that crossed unity for broncho-alveolar lavage, bronchoscopy, CPR, extubation, extubation/intubation combined, mechanical ventilation, oxygen administration, and tracheostomy. The study designs and HCW role varied, and sample sizes were small or not reported. In a prospective cohort of 414 participants, the risk ratio of contracting COVID-19 after participating in airway suctioning was 1.67 (*p* = 0.04), and after participating in manual ventilation the risk ratio was 3.1 (*p* = 0.008) [45]. In a small cohort study, being present for or assisting with nebulizer treatments was more common among HCWs who developed COVID-19 than among those who did not (*p* = 0.04) [48]. In a case–control study of 751 participants, the odds of contracting COVID-19 if the HCW was exposed to intubation (exposure not defined) was 2.5 (95% CI 1.13 to 5.5) [28]. Only three of the nine studies adjusted for confounding factors in their analysis. Open airway suctioning in the Lentz et al. [32] study had a lower risk of transmission to HCWs (aOR 0.48, 95% CI 0.25–0.90) after adjusting for age, gender, smoking status, presence of baseline comorbidities, healthcare worker role, and world region. The other two studies did not show a change in the relative effect of COVID-19 transmission risk to HCWs in the presence of specific AGMPs [28, 36].

**Table 4:** Summary of findings from the studies that included statistical comparisons with respect to transmission of COVID-19 during specific AGMPs*

AGMP	HCW role during AGMP	Author	Study design	Sample size	Odds ratio^a^ (95%CI)
Airway Suctioning	Participated in	Nakagama [45]	Prospective cohort	AGMP: n = 202 Control: n = 202	**RR: 1.67(NR); *p* = 0.04**
	NR; sputum suction	Ran [42]	Retrospective cohort	AGMP: n = 11 Control: n = 61	RR: 0.43 (0.12 to 1.55)
	Performed or assisted, or present in room	Heinzerling [48]	Cohort	AGMP: n = 7 Control: n = NR	AGMP cases: 0 Control cases: NR *p* = 1.00
	NR	Chatterjee [28]	Case–control	AGMP: n = 35 Control: n = 716	0.73 (0.37 to 1.45)
	Performed open suctioning	Lentz [32]	Case–control	AGMP: n = NR Control: n = NR	0.55 (0.3 to 1.01)
	Performed closed suctioning	Lentz [32]	Case–control	AGMP: n = NR Control: n = NR	0.89 (0.57 to 1.39)
Broncho-Alveolar Lavage	Performed (> 5 times)	Lormans [36]	Cohort	AGMP: n = NR Control: n = NR	0.42 (0.05 to 3.53)
Bronchoscopy	NR	Ran [42]	Retrospective cohort	AGMP: n = 1 Control: n = 71	RR: 0.63 (0.06 to 7.08)
	Performed or assisted	Heinzerling [48]	Cohort	AGMP: n = 3 Control: n = NR	AGMP cases: 0 Control cases: NR *p* = 1.00
	Present in room	Heinzerling [48]	Cohort	AGMP: n = 3 Control: n = NR	AGMP cases: 0 Control cases: NR *p* = 1.00
	Performed rigid bronchoscopy	Lentz [32]	Case–control	AGMP: n = NR Control: n = NR	0.256 (0.03 to 2.0)
	Witnessed/assisted rigid bronchoscopy	Lentz [32]	Case–control	AGMP: n = NR Control: n = NR	1.09 (0.3 to 4.0)
	Performed	Lentz [32]	Case–control	AGMP: n = NR Control: n = NR	0.55 (0.3 to 1.03)
	Witnessed/assisted	Lentz [32]	Case–control	AGMP: n = NR Control: n = NR	0.7 (0.32 to 1.51)
	Performed	Caglayan [34]	Cross-sectional	AGMP: n = 31 Control: n = 122	AGMP cases:3 Control cases:5 *p* = 0.21
CPR	Participated in	Nakagama [45]	Prospective cohort	AGMP: n = 13 Control: n = 202	RR: 1.94 (NR); *p* = 0.214
	NR	Ran [42]	Retrospective cohort	AGMP: n = 1 Control: n = 71	RR: 0.63 (0.06 to 7.08)
	Performed	Celebi [29]	Case–control	AGMP: n = 20 Control: n = 161	AGMP cases:6 Control cases:41 *p* = 0.629
	Advanced airway throughout	Lentz [32]	Case–control	AGMP: n = NR Control: n = NR	1.06 (0.5 to 2.25)
	No advanced airway at some point	Lentz [32]	Case–control	AGMP: n = NR Control: n = NR	1.32 (0.61 to 2.86)
Extubation	NR	Ran [42]	Retrospective cohort	AGMP: n = 1 Control: n = 71	0.63 (0.06 to 7.08)
	Performed	Lentz [32]	Case–control	AGMP: n = NR Control: n = NR	0.55 (0.25 to 1.24)
	Witnessed or assisted	Lentz [32]	Case–control	AGMP: n = NR Control: n = NR	1.29 (0.68 to 2.47)
Intubation/Extubation	Participated in	Nakagama [45]	Prospective cohort	AGMP: n = 21 Control: n = 202	RR: 0.8 (NR); *p* = 1
Intubation	Performed or assisted	Heinzerling [48]	Cohort	AGMP: n = 2 Control: n = NR	AGMP cases: 1 Control cases: NR *p* = 0.16
	Present in room	Heinzerling [48]	Cohort	AGMP: n = 1 Control: n = NR	AGMP cases: 0 Control cases: NR *p* = 1.00
	Performed (> 1 time)	Lormans [36]	Cohort	AGMP: n = NR Control: n = NR	0.23 (0.03 to 1.98)
	Performed or present in room	Celebi [29]	Case–control	AGMP: n = 27 Control: n = 154	AGMP cases:6 Control cases:41 *p* = 0.705
	NR	Chatterjee [28]	Case–control	AGMP: n = 31 Control: n = 720	**2.5 (1.13 to 5.5)**
	Performed	Lentz [32]	Case–control	AGMP: n = NR Control: n = NR	0.73 (0.38 to 1.37)
	Witnessed or assisted	Lentz [32]	Case–control	AGMP: n = NR Control: n = NR	0.74 (0.44 to 1.26)
Manual Ventilation	Participated in	Nakagama [45]	Prospective cohort	AGMP: n = 19 Control: n = 202	**RR: 3.1(NR); *p* = 0.008**
	Performed or assisted, or present in room (BiPAP, CPAP)	Heinzerling [48]	Cohort	AGMP: n = 6 Control: n = NR	AGMP cases: 2 Control cases: NR *p* = 0.06
	Performed or assisted, or present in room	Heinzerling [48]	Cohort	AGMP: n = 3 Control: n = NR	AGMP cases: 1 Control cases: NR *p* = 0.23
	Adjusted NIPPV mask	Lentz [32]	Case–control	AGMP: n = NR Control: n = NR	**0.6 (0.38 to 0.95)**
	Present during use of NIPPV	Lentz [32]	Case–control	AGMP: n = NR Control: n = NR	0.71 (0.49 to 1.04)
Mechanical Ventilation	Present in room	Celebi [29]	Case–control	AGMP: n = 42 Control: n = 139	AGMP cases:9 Control cases:38 *p* = 0.484
Nebulizer	Administration	Nakagama [45]	Prospective cohort	AGMP: n = 8 Control: n = 202	RR: 1.05(NR); * p* = 1
	Sputum induction	Nakagama [45]	Prospective cohort	AGMP: n = 12 Control: n = 202	RR: 2.8(NR); * p* = 0.055
	Performed or assisted, or present in room	Heinzerling [48]	Cohort	AGMP: n = 5 Control: n = NR	**AGMP cases: 2** **Control cases: NR** ***p* = 0.04**
	Present during delivery	Lentz [32]	Case–control	AGMP: n = NR Control: n = NR	1.11 (0.69 to 1.8)
Oxygen administration	Applied or adjusted patient's HFNC	Lentz [32]	Case–control	AGMP: n = NR Control: n = NR	0.98 (0.65 to 1.47)
	Present during use	Lentz [32]	Case–control	AGMP: n = NR Control: n = NR	1.05 (0.74 to 1.47)
Tracheostomy	Oxygen supplementation via tracheostomy	Nakagama [45]	Prospective cohort	AGMP: n = 63 Control: n = 202	RR: 1.07 (NR); * p* = 0.83
	Exposed to with any role (first operator, fiberoscopist, instrumental nurse, or anesthesia nurse)	Rosano [38]	Cohort	AGMP: n = 91 Control: n = 52	AGMP cases:7 Control cases:6 *p* = 0.55
	Performed open tracheostomy	Lentz [32]	Case–control	AGMP: n = NR Control: n = NR	1.04 (0.21 to 5.03)
	Witnessed or assisted open tracheostomy	Lentz [32]	Case–control	AGMP: n = NR Control: n = NR	0.99 (0.27 to 3.58)
	Performed percutaneous tracheostomy	Lentz [32]	Case–control	AGMP: n = NR Control: n = NR	0.45 (0.06 to 3.63)
	Witnessed or assisted percutaneous tracheostomy	Lentz [32]	Case–control	AGMP: n = NR Control: n = NR	0.78 (0.22 to 2.72)

Bolded estimates represent statistically significant results (p < 0.05). Details pertaining to patients and settings and studies that only reported count data but no statistical comparisons can be found in Table 2

*AGMP* aerosol-generating medical procedure; *CI* confidence interval; *HCW* healthcare worker; *HFNC* high flow nasal cannula; *NIPPV* noninvasive positive-pressure ventilation; *NR* not reported; *RR* risk ratio

^a^Unadjusted Odds Ratio and 95% confidence interval unless otherwise specified

### SARS

#### Not specifically identified AGMPs

No studies reported statistical associations for the transmission of SARS to HCWs after exposure to AGMPs not specifically identified.

#### Specific AGMPs

Two studies reported count data only and did not conduct a statistical analysis for the association between specific AGMPs and the transmission of SARS [57, 61]. Eight studies reported statistical associations for nine specific AGMPs and the transmission of SARS including: airway suctioning (n = 2) [55, 63], bronchoscopy (n = 1) [63], CPR (n = 3) [55, 62, 63],intubation (n = 7) [55, 58, 60, 62–65], manual ventilation (n = 3) [55, 59, 63], mechanical ventilation (n = 1) [55], nebulizer administration (n = 1) [63], oxygen administration (n = 3) [55, 58, 63],and tracheostomy (n = 1)[65] (Table 5). All reported associations were non-significant for bronchoscopy and mechanical ventilation. An increased risk of SARS to HCWs, that reached statistical significance, was observed for airway suctioning, CPR, intubation, manual ventilation, nebulizer administration, oxygen administration, and tracheostomy. Notably, all seven studies reported an elevated risk of SARS when participating in intubation, and all but one study reached statistical significance at *p* < 0.005.

**Table 5:** Summary of findings from studies that included statistical comparisons with respect to transmission of SARS for Specific AGMPs*

AGMP	AGMP activity	Author	Study design	Sample size	Odds ratio^a^ (95%CI)
Airway Suctioning	After intubation	Loeb [63]	Retrospective cohort	AGMP: n = 19 Control: n = 13	0.68 (0.21 to 2.26)
	Before intubation	Loeb [63]	Retrospective cohort	AGMP: n = 4 Control: n = 28	**4.2 (1.58 to 11.14)**
	Endotracheal aspirate	Loeb [63]	Retrospective cohort	AGMP: n = 12 Control: n = 20	1.0 (0.29 to 3.45)
	Present in room after intubation	Raboud [55]	Retrospective cohort	AGMP: n = 165 Control: n = 459	1.63 (0.97 to 2.73)
	Present in room before intubation	Raboud [55]	Retrospective cohort	AGMP: n = 113 Control: n = 511	1.78 (0.97 to 3.29)
Bronchoscopy	Performed	Loeb [63]	Retrospective cohort	AGMP: n = 2 Control: n = 30	2.14 (0.46 to 9.9)
CPR	Performed	Loeb [63]	Retrospective cohort	AGMP: n = 3 Control: n = 29	AGMP cases: 0 Control cases: 8 *p* = 0.55
	Present in room during compressions	Raboud [55]	Retrospective cohort	AGMP: n = 9 Control: n = 615	**6.39 (3.27 to 12.5)**
	Present in room during defibrillation	Raboud [55]	Retrospective cohort	AGMP: n = 4 Control: n = 620	**15.73 (5.27 to 46.9)**
	Close proximity (< 1 m)	Liu [62]	Case–control	AGMP: n = 15 Control: n = 462	**AGMP cases: 5** **Control cases: 51** ***p* = 0.02**
Intubation	Performed	Chen [65]	Retrospective cohort	AGMP: n = 33 Control: n = 715	**8.03 (3.9 to 16.56)**
	Performed or assisted	Fowler [64]	Retrospective cohort	AGMP: n = 14 Control: n = 62	**RR: 13.29 (2.99 to 59.04)**
	Performed	Loeb [63]	Retrospective cohort	AGMP: n = 4 Control: n = 28	**4.2 (1.58 to 11.14)**
	Present in room	Raboud [55]	Retrospective cohort	AGMP: n = 144 Control: n = 480	**2.92 (1.7 to 5.03)**
	Close proximity (< 1 m)	Liu [62]	Case–control	AGMP: n = 12 Control: n = 465	**AGMP cases: 6** **Control cases: 45** ***p* = < 0.001**
	Performed/Assisted	Teleman [58]	Case–control	AGMP: n = 6 Control: n = 80	1.5 (0.4 to 5.4)
	Performed	Pei [60]	Case–control	AGMP: n = 37 Control: n = 406	**30.79 (7.91 to 119.84)**^**b**^
Manual Ventilation	Performed	Loeb [63]	Retrospective cohort	AGMP: n = 7 Control: n = 25	1.19 (0.3 to 4.65)
	Present in room during on-invasive ventilation	Raboud [55]	Retrospective cohort	AGMP: n = 109 Control: n = 515	1.37 (0.14 to 13.1)
	Present in room during manual ventilation after intubation	Raboud [55]	Retrospective cohort	AGMP: n = 120 Control: n = 504	**1.83 (1.09 to 3.07)**
	Present in room during manual ventilation before intubation	Raboud [55]	Retrospective cohort	AGMP: n = 118 Control: n = 506	**2.68 (1.34 to 5.35)**
	Present > 30 min during use of NPPV	Scales [59]	Retrospective cohort	AGMP: n = 22 Control: n = 9	105 (3 to 3035)
Mechanical Ventilation	Present in room	Raboud [55]	Retrospective cohort	AGMP: n = 236 Control: n = 388	1.06 (0.49 to 2.3)
Nebulizer	Performed	Loeb [63]	Retrospective cohort	AGMP: n = 5 Control: n = 27	**3.24 (1.11 to 9.42)**
Oxygen administration	Manipulated BiPAP mask	Loeb [63]	Retrospective cohort	AGMP: n = 6 Control: n = 26	2.6 (0.8 to 7.99)
	Manipulated oxygen mask	Loeb [63]	Retrospective cohort	AGMP: n = 14 Control: n = 18	**9 (1.25 to 64.89)**
	Present in room during procedure	Raboud [55]	Retrospective cohort	AGMP: n = 108 Control: n = 516	0.67(0.43 to 1.04)
	Performed	Teleman [58]	Case–control	AGMP: n = 17 Control: n = 69	1.0 (0.3 to 2.8)
Tracheostomy	Performed	Chen [65]	Retrospective cohort	AGMP: n = 17 Control: n = 731	**4.15 (1.5 to 11.5)**

Bolded estimates represent statistically significant results (*p* < 0.05). Details pertaining to patients and settings and studies that only reported count data but no statistical comparisons can be found in Table 2

*AGMP* aerosol-generating medical procedure; *CI* confidence interval; *RR* risk ratio

^a^Odds Ratio unless otherwise specified

^b^Adjusted odds ratio reported because unadjusted odds ratios could not be found

### MERS

One study looked at MERS infection among a cohort of HCWs who reported direct contact (i.e. within 2 m) with a MERS patient in the hospital. No statistically significant difference in seropositivity in HCWs was observed for manipulation of oxygen face mask or tubing, airway suction, non-invasive ventilation, manual ventilation, nebulizer treatments, intubation, CPR, high-frequency oscillatory ventilation, bronchoscopy, or extubation. Overall, there was no statistically significant difference in seropositivity in HCWs present in the room when any of these procedures were performed [54].

#### Other coronaviruses

In the study looking at various coronavirus strains HKU1, OC43, NL63, 229E, HCWs were 1.77 times more likely to contract the virus if they participated in airway suctioning (OR = 1.77, 95% CI: 1.19 to 2.57), 2.01 times if they participated in nasopharyngeal aspiration (OR = 2.01, 95% CI: 1.27 to 3.04), and 1.81 times if they participated in nebulizer treatment (OR = 1.81, 95% CI: 1.34 to 2.42); no statistically significant differences were observed for intubation; a pooled OR of all these activities revealed that HCWs who participated in them were 2.05 times more likely to contract any one of the human CoVs (OR = 2.05, 95% CI: 1.21 to 3.4) [51]. This risk was reduced but remained significant (aOR 1.80, 95% CI 1.04–3.1) when adjusting for age, household members under the age of 5 years, whether participants saw adult, pediatric or both patient populations, the proportion workdays with exposure to patients or co-workers with respiratory illness, and occupational risk level [51].

### Other pathogens

Across the included studies, three reported on influenza [52, 53, 56], including one that reported on several different pathogens (adenoviruses, human metapneumovirus (HMP), coronaviruses 229E/NL63 and OC43/HKU1, parainfluenza viruses 1, 2 and 3, influenza viruses A and B, respiratory syncytial virus (RSV) A and B, and rhinovirus A/B; *Streptococcus pneumoniae, Mycoplasma pneumoniae, B. pertussis, Legionella spp, Chlamydophilia and Haemophilus* i*nfluenzae* type B, combined influenza with human coronavirus 229E/NL63, rhinoviruses, and respiratory syncytial viruses) [56]. These three studies did not report statistical comparisons for specific AGMPs.

#### Influenza

Two of the three influenza studies reported statistical comparisons between the AGMP and non-AGMP groups; in both studies, there were no statistically significant differences in VRI rates between HCWs in AGMP and non-AGMP groups [52, 56]. One study adjusted for other variables (e.g. number of hours worked, number of patients the HCW was in contact with, number of contacts with patients with ILI) and found no significant association between influenza and AGMPs [56].

#### Multiple pathogens

In one study examining various pathogens, HCWs performing AGMPs were 2.5 times more likely to contract the respiratory infection based on the presence of respiratory symptoms (OR = 2.5, 95% CI: 1.3 to 6.5; *p* < 0.01); this effect, however, was not significant when looking only at laboratory-confirmed viral infection by influenza combined with human coronavirus 229E/NL63, rhinoviruses, and respiratory syncytial viruses (OR = 2.8, 95% CI: 0.9 to 8.7, *p* = 0.07) [56]. However in a more robust Poisson regression analysis, adjusted for HCW age, education, occupation, history of influenza vaccine, and hand hygiene for the outcome of laboratory-confirmed viral infection, the RR of 3·3 (95% CI 1·01–11·02, *p* = 0·05) was significantly associated with the performance of an AGMP (defined as nebulizer medications, suctioning, intubation, aerosol-generating procedures and chest physiotherapy). Respiratory airway suctioning was the most common AGMP with 66% of respondents reported performing this procedure. Respondents represented a convenience sample of HCWs from a control group of a larger RCT, who did not wear a mask and who did not have any interventions on the use of masks or respirators and being observed in their usual working conditions [67].

### Particulate respirators versus medical/surgical masks

Eight studies examined transmission of VRIs during AGMPs when comparing the use of N95 versus other PPE [32, 40, 41, 46, 49, 50, 54, 55]. Six of these studies examined the transmission of COVID-19 [32, 40, 41, 46, 49, 50], one on SARS [55], and one on MERS [54]. Sample sizes for this comparison ranged from seven [41] to 624 [55]. Five of the studies reported statistical comparisons between particulate respirators and medical/surgical masks and VRI transmission during AGMPs and are further described below [32, 40, 49, 54, 55].

#### COVID-19

An international case–control study reported that wearing a respirator (N95/FFP2/FFP3/PAPRs/reusable elastomeric respirators) during AGMPs and non-AGMPs had a 60% reduced risk (aOR 0.4, 95%CI: 0.2–0.8, *p* = 0.005) of contracting COVID-19 when exposed to COVID-19 patients, versus HCWs who did not report wearing a respirator during AGMPs and non-AGMPs. However, the use of medical masks during both AGMPs and non-AGMPs was associated with HCW infection (aOR 7.4, 95%CI 2.8–20.0, *p* < 0.001) [32]. Respirator use during most individual AGMPs (intubation witness or assistant, performed extubation, performed closed suctioning, present during delivery of nebulized medication, present during NIPPV use, adjusted or applied patients NIPPV mask, performed bronchoscopy, bronchoscopy witness or assistant) demonstrated OR < 1.0 and aOR < 1.0 but many did not meet statistical significance. The use of respirators compared to non-respirators while performing open suctioning was protective against COVID-19 (OR 0.4 95% CI 0.18–0.96, *p* = 0.0396; aOR 0.3, 95%CI 0.12–0.80, *p* = 0.0159) [32].

In a retrospective cohort study where all HCW used either a respirator or surgical mask during an AGMP, they found the use of a surgical mask instead of respirator during AGMP was not associated with transmission of COVID-19 even in the context of a prolonged (≥ 5 min) close contact (< 2 m or 6 ft) to a patient with confirmed COVID-19 (RR 0.99; 95% CI 0.96–1; *p* = 1) [40].

In a prospective cohort study, HCWs performing AGMPs with universal use of FFP2 compared to wearing surgical masks (either only, mostly, or equally to FFP2) during AGMPs irrespective of a patient’s COVID-19 status showed no effect in the outcome of SARS-CoV-2 positive swab using Cox regression (aHR 1.08 95% 0.71–1.64, *p* = 0.726) or in the outcome of SARS-CoV-2 seroconversion using multivariable logistic regression (aOR 0.89, 95% CI 0.62–1.28, *p* = 0.535) [49].

Three other studies reported the use of respirators (N95/KN95/FFP2) during AGMPs as either increasing or reducing the risk of COVID-19, however it was not clear whether this was compared to medical/surgical masks [41, 46, 50].

An additional two studies reported the risk of COVID-19 among HCWs exposed to AGMPs compared to non-AGMP exposures, where the AGMP group was stratified by appropriate/systematic or sub-optimal/non-systematic use of masks [39, 44]. For AGMPs with self-declared appropriate, or sub-optimal mask use there was an increased association with a positive test for SARS-CoV-2 IgG (OR 1.37, 95% CI 1.04 to 1.81; and OR 1.74, 95% CI 1.05 to 2.88, respectively) [44]. The systematic use of FFP2 during AGMPs compared to non-AGMP exposures, decreased the serological prevalence of COVID-19 when compared to the serological prevalence of COVID-19 after exposure to AGMPs without the systematic use of FFP2 (OR 1.70; 95% CI 1.15 to 2.49, and OR 2.03; 95% CI 1.36 to 3.02, respectively); however the 95% CI cross each other and therefore there is no statistical significance between AGMP exposures [39].

#### SARS and MERS

In a retrospective cohort of MERS, HCWs who reported always covering their nose and mouth with either a medical mask or N95 respirator while present in the room during an AGMP had a 68% lower risk for infection than HCWs reporting not always or never doing so (RR 0.32 95%CI 0.12–0.86, *p* = 0.03); however when medical masks or N95 respirators always worn during AGMPs were evaluated independently and compared to sometimes/never worn during AGMPs, both showed a protective effect but were not statistically significant [54].

A study on the risk of SARS to HCWs providing care to intubated SARS patients showed that HCWs who contracted SARS were more likely to have used less effective methods of respiratory protection while in a patient’s room (Cochran-Armitage test for trend for no respiratory protection, to surgical mask, to N95 or equivalent to protection higher than N95, *p* = 0.04) [55].

## Discussion

Thirty-eight studies were identified in this systematic review on the risk of viral transmission in the presence of AGMPs, with 60% of studies focused on COVID-19 and therefore published within the past two years. Based on the studies assessed in this review, exposure to an AGMP may increase the risk of respiratory viral transmission to HCWs, however the evidence base is inconsistent, particularly related to the transmission of COVID-19. Most of the studies were on COVID-19 and SARS with only three studies on influenza, one study on other human coronaviruses, one on MERS and one study with multiple respiratory viruses where the HCWs were a no-mask control group for a clinical trial [67]. For COVID-19, of the 16 studies that reported unadjusted results, two studies revealed an increased risk of transmission of COVID-19 to HCWs exposed to either not specified or combined AGMPs, reaching statistical significance. When adjusting for different confounders, the results also varied between demonstrating an increased risk of transmission and not. These were cross-sectional studies and one was a pre-print manuscript not yet peer-reviewed [46] but they were among the largest of the studies. One prospective cohort study found a trend towards a significant association for all AGMPs combined and found significant associations of transmission with specific AGMPs [45]. This study used both RT-PCR and serology for case ascertainment. Studies that did not show a statistically significant relationship between AGMPs and COVID-19 varied in study design, AGMPs and HCW role during the AGMP, and sample sizes were small or not reported.

Eight of the ten SARS studies reported statistical associations for nine specific AGMPs and the transmission of SARS. Six of these studies demonstrated increased risk of transmission of SARS with a specific AGMP, reaching statistical significance. Of the two studies that did not report a statistical association between a specific AGMP and the transmission of SARS, one reported proportions of HCWs who did not develop SARS with exposure to nebulized medications or NIPPV [61] and the other studied medical students present in a room during nebulization therapy but who had been previously exposed from a different source [57], which compromises meaningful interpretation. All but one study reported associations for SARS transmission during intubation that were statistically significant including the two moderately rated studies on risk of bias. The one study on human coronavirus infections reported statistically significant associations with various AGMPs and for the one MERS study there was no statistically significant difference in seropositivity in HCWs across any of the specific AGMPs. Another study on mixed VRIs also revealed statistically significant associations for transmission to HCWs during the performance of AGMPs [56].

A systematic review previously published by Tran et al. [14] also evaluated the risk of transmission of acute respiratory infections to HCWs exposed to AGMPs. Their review identified ten studies, all investigating the risk of transmission of SARS to HCWs exposed and not exposed to AGMPs. They concluded that some procedures potentially capable of generating aerosols were epidemiologically associated with an increased risk of SARS transmission to HCWs, with the most consistent association observed with tracheal intubation [14]. However, the precision of the reported statistical associations was wide among the studies included in the Tran et al. review and they also acknowledged that the study quality was low. Nonetheless, the associations were quite consistent across the studies which added epidemiologic strength to the association with respect to SARS. Only one of the five studies in this review was found to have a significant statistical association between tracheal intubation and the risk of COVID-19, however details about the patient exposure were not provided [28].

There was limited evidence supporting the use of a particulate respirator over a medical/surgical mask during an AGMP to reduce the risk of viral transmission. Only one study reported a statistically significant, 60% reduction in the risk of contracting COVID-19 among HCWs wearing a respirator during an AGMP [32]. One study indicated that risk of infection during an AGMP (across various viral pathogens) was higher amongst HCWs who did not wear a mask or respirator as compared to those who did [56]. RCTs and cluster-RCTs comparing N95 respirators and medical/surgical masks have primarily been conducted during routine care of patients, not AGMPs, with VRIs in healthcare settings (n = 5 studies) and households (n = 1 study). These studies revealed that N95 respirators compared to medical/surgical masks made no significant differences in transmission of laboratory-confirmed influenza for routine care with moderate certainty evidence [68].

No conclusions could be made on the relative risk of VRI to HCWs during AGMPs between pediatric and adult patient populations. The lack of pediatric specific data makes it challenging to draw firm conclusions or make recommendations for those HCWs caring for pediatric patients. Only one small study described the outcomes of 11 HCWs exposed to a pediatric (17-year-old) patient with asymptomatic COVID-19 during endotracheal intubation. Nine of the HCWs did not wear N95 respirators during the procedure, and no cases of COVID-19 occurred [41]. In the post-hoc analysis of the ResPECT study, HCWs that saw pediatric patients had a 57% increased odds of coronavirus infection compared to those that saw only adult patients [51]. However, there was no analysis assessing whether this risk was related to AGMPs or not. Based on the recent environmental scan of pediatric facilities in Canada, no consensus on the use of respirators for endemic or emerging/re-emerging respiratory pathogens has been clearly established.

Ninety-five per cent of the studies had an overall serious or critical risk of bias. First, there were issues related to study design. Many studies were cross-sectional or retrospective observational studies, limiting our understanding of directionality between AGMP exposure and the VRI outcome.

The comparison of HCWs performing AGMPs vs not performing AGMPs is difficult, indirect, and prone to significant confounding. Most studies did not consider or control for confounding factors when assessing the risk of VRI in the presence of AGMPs such as the use of PPE, disease status of the patient, days from symptom onset, vaccination status of the patient and/or HCWs, exposure time to the patient with VRI, training and experience of the AGMP operator, or other potentially clinically relevant details such as whether or not anesthesia or paralysis is involved with procedures such as intubation. Some clinical scenarios are managed in ways that may ultimately reduce the risk of transmission that effectively negate aerosol generation or regardless of which respiratory protection is used. Consideration of community-level prevalence, increased transmissibility of variants of concern in the context of COVID-19, and procedure room ventilation, were not discussed.

Second, no genomic testing of viral pathogens among HCWs and patients were reported to verify transmission of the same strain.

Third, many studies used questionnaires to retrospectively collect exposure information from HCWs at varying follow-up periods, introducing recall bias in the study and further limiting our understanding of the directionality between AGMP exposure and the VRI outcome.

Finally, over half of the studies did not list specific AGMPs but instead combined multiple procedures into the category of AGMP or did not define their AGMP group. Lack of describing the AGMP and identifying potential confounders precludes distinguishing whether the increased risk of VRI during AGMPs is due to the procedure itself or the circumstances in which the procedures are being performed [4, 14, 69, 70]. For example, the increased risk observed among certain procedures may be explained by symptoms experienced by the patient, viral load, proximity of the HCW to the patient during the procedure, and duration of the exposure [4, 70]. Similarly, PPE use was poorly reported, and the effect of PPE was seldom controlled for in the statistical comparisons within the studies. Where it was reported, it was self-reported leaving room for recall bias or it was not always measured in the same way (e.g., percentage of time PPE was used properly vs. dichotomous measure of always or sometimes used). Therefore, it is unknown if the difference in risk among AGMPs was due to exposure to the AGMP itself or exposure to an AGMP with inappropriate PPE or lapses in adherence to PPE.

Another issue highlighted by this systematic review was the lack of a definitive and comprehensive list of AGMPs for healthcare settings. Tran et al. [14] acknowledged the presence of a significant research gap in the epidemiology of the risk of transmission of VRI from patients undergoing AGMPs to HCWs and the difficulty in defining AGMPs. Specifically, the scientific evidence for the creation of aerosols associated with the procedures and the burden of infectious microbes within the created aerosols, and the mode of transmission were not well studied. In their conclusions, they suggested that funding agencies should focus attention on this area. Many organizations or professional societies have itemized AGMPs [3, 4, 8–13] but not all of these procedures were identified based on formal measures of potential infectious aerosols produced by the procedure or robust epidemiological studies demonstrating increased risk of infection to HCWs or others exposed to patients undergoing these procedures [69]. This continue to be the case, as most procedures listed as AGMPs have limited data characterizing the creation and dispersion of aerosols and the quantification of infectious agents from the aerosols [71, 72]. Therefore, further rigourous multidisciplinary research is needed to understand the relative increase in infectious aerosols by these procedures relative to symptoms of illness that may generate aerosols (e.g. coughing and sneezing).

We also agree that more rigorous studies should be conducted to understand the factors that lead to increased transmission risk, which not only consider the procedures that are being performed but also the specific pathogens, the circumstances surrounding the patient with respect to the procedure and their state of illness and immunity; and HCW related factors such as immunity, PPE, and general health.

In this review, pooled effect sizes were not calculated due to the heterogeneity in the studies including variations in the jurisdiction, viral pathogen, type of AGMP, and consideration of confounding factors. The precision of the reported statistical associations was wide across several studies making it difficult to infer the true effect size. We were unable to identify sufficient evidence to make any conclusions regarding the type of mask (medical/surgical mask or respirator) that should be used during AGMPs. Specific AGMPs could not be delineated from the grouped analyses in most studies on SARS-CoV-2 and therefore risk could not be assigned to individual procedures. Although some studies examined the risk of VRI among HCWs caring for pediatric and adult patients undergoing AGMPs, the ability to pull the pediatric information into a discrete number was not possible.

Despite these limitations our systematic review has several strengths including the comprehensive search, inclusion of medical procedures traditionally considered AGMPs, the inclusion of procedures identified by consensus by our AGMP working group which expanded upon the traditional list; a search for studies that assessed the risk to HCWs during AGMPs in pediatric populations specifically, and the evaluation of studies exploring the role of medical/surgical masks versus respirators during AGMPs. We did not identify any recent studies after our search date that would change our overall findings. In addition, this review looked at all members of the coronavirus family and was unique in this approach and found there may be an increased risk of transmission with AGMPs and of any of the coronaviruses except for MERS.

## Conclusion

The findings from this systematic review, mainly of unadjusted comparisons from observational studies, suggest that there may be an elevated risk of transmission with AGMPs for SARS, COVID-19, and human coronaviruses. What proportion of that effect is due to confounding, and what proportion is due to a true effect is unknown and needs careful evaluation in future studies of higher quality that take confounding into consideration. Evidence of the difference in protection between medical masks and N95 respirators is still lacking, but HCWs who did not wear masks or had low masking compliance were found to be at an increased risk of transmission with many of the respiratory viruses studied. It is important to not abandon the concept of the increased risk of transmission associated with AGMPs as some have suggested [70]. It may be especially important for emerging pathogens, and as Palmore and Henderson have pointed out, the current protocols have served us well [73]. Our review continues to identify the presence of significant research gaps in the epidemiology of the risk of VRI among HCWs during AGMPs, and unfortunately little progress has occurred since the study by Tran et al. [14] who originally suggested this area should be a priority for research and policymakers. This research gap is compounded by the lack of precision in the literature regarding the definition of AGMPs and the circumstances surrounding these and other procedures that may increase risk to HCWs. The gap is also particularly true for HCWs providing care to pediatric patients.

## Supplementary Information

**Additional file 1**. Complete Search Strategy. Outlines the search strategies used across all databases.

**Additional file 2**. List of AGMPs Considered in the Context of this Review. List of all the AGMPs considered in the context of this review, based on existing AGMP lists and by consensus from the Alberta Health Services’ AGMP guidance tool.

**Additional file 3**. List of Excluded Studies. List of excluded studies and main reason for exclusion.

## Data Availability

Not applicable.

## Abbreviations

AGMPs: Aerosol-generating medical procedures
aHR: Adjusted hazard ratio
aOR: Adjusted odds ratio
CDC: Centers for disease control and prevention
CI: Confidence interval
COVID-19: Coronavirus disease 2019
Covid-END: COVID-10 evidence network to support decision-making
CPR: Cardiopulmonary resuscitation
EBM: Evidence-based medicine
ELISA: Enzyme-linked immunoassay
EMT: Emergency medical technician
FFP: Filtering face piece
HCWs: Healthcare workers
HFNC: High flow nasal cannula
IRR: Incidence rate ratio
L-OVE: Living overviews of evidence
NIPPV: Non-invasive positive-pressure ventilation
NP: Nurse practitioner
NR: Not reported
OR: Odds ratio
PA: Physicians assistant
PAPR: Powered air purifying respirators
PHSM: Public health and social measures
PPE: Personal protective equipment
PR: Prevalence ratio
PRESS: Peer review electronic search strategies
PRISMA: Preferred reporting items for systematic reviews and meta-analyses
RCT: Randomized controlled trial
RT-PCR: Real-time polymerase chain reaction
ResPECT: Respiratory protection effectiveness clinical trial
ROBINS-I: Risk of bias in non-randomised studies–of interventions
RN: Registered nurse
RT: Respiratory therapist
SARS: Severe acute respiratory syndrome
SARS-CoV-2: Severe acute respiratory syndrome coronavirus-2
MERS: Middle Eastern respiratory syndrome
VRI: Viral respiratory infection
WHO: World Health Organization
