# Supplementary material for: Risk of transmission of respiratory viruses during aerosol-generating medical procedures (AGMPs) revisited in the COVID-19 pandemic: a systematic review
Source: Antimicrob Resist Infect Control. 2022 Aug 11;11:102. doi: 10.1186/s13756-022-01133-8 (PMC9366810; doi:10.1186/s13756-022-01133-8)
Supplement: Supplementary file 1 — Additional file 1. Complete Search Strategy. Outlines the search strategies used across all databases. [file 13756_2022_1133_MOESM1_ESM.docx]

**Additional File 1: Complete Search Strategy**

Aerosol Transmission – HCWs

Final Searches

2021 Sep 8-9

*2021 Sep 8*

Ovid Multifile

Database: EBM Reviews - Cochrane Central Register of Controlled Trials <August 2021>, EBM Reviews - Cochrane Database of Systematic Reviews <2005 to September 01, 2021>, Embase <1974 to 2021 September 07>, Ovid MEDLINE(R) and Epub Ahead of Print, In-Process, In-Data-Review & Other Non-Indexed Citations and Daily <1946 to September 07, 2021>

Search Strategy:

--------------------------------------------------------------------------------

1 exp Aerosols/ (94139)

2 (aerosoli#e* or aerosoli#ing).tw,kf. (17860)

3 (aerosol* adj3 (contact* or dispers* or expos* or generat* or procedure* or produc* or spread* or transmi*)).tw,kf. (20624)

4 ((AGP or AGPs or AGMP or AGMPs or AGDP or AGDPs) and aerosol*).tw,kf. (307)

5 (aerosol* adj5 (clinic* or dent* or medic* or therap* or treatment*)).tw,kf. (11016)

6 (bioaerosol* or bio-aerosol*).tw,kf. (4341)

7 ("3-in-1" adj2 syringe?).tw,kf. (29)

8 (water adj1 air? adj2 syringe?).tw,kf. (230)

9 ((air rota or air rotar*) and dent*).tw,kf. (1)

10 (((polish* or rotar* or finishing or handpiece* or hand piece*) adj3 (device* or equipment* or instrument*)) and dent*).tw,kf. (2061)

11 ((ultrasonic* or sonic*) adj3 (scale* or scaling)).tw,kf. (2001)

12 (air polish* or air abrasion* or air turbine handpiece? or air turbine hand piece?).tw,kf. (2093)

13 (dent* adj3 (handpiece* or hand piece*) adj5 (highspeed* or high speed* or lowspeed* or low speed*)).tw,kf. (159)

14 Autopsy/ (195259)

15 (autops* or postmortem exam* or post-mortem exam*).tw,kf. (196968)

16 bag-valve*.tw,kf. (1649)

17 (BVM adj10 (bag? or valve? or mask*)).tw,kf. (393)

18 (ambu bag? or bag-mask? or (bag? adj1 resuscitat*) or (manual* adj1 resuscitat?) or self-inflating bag? or selfinflating bag? or "Revivator-Plus").tw,kf. (2518)

19 exp Positive-Pressure Respiration/ (40077)

20 ((positive end-expiratory or (positive adj2 pressure)) adj (breathing or ventilat*)).tw,kf. (18621)

21 positive airway pressure.tw,kf. (35711)

22 (PEEP and (positive or expiratory or pressure)).tw,kf. (14310)

23 (APRV or BiPAP or BPAP or CPCP or nCPAP or CPPB or CPPV or IPPB or IPPV).tw,kf. (11263)

24 Noninvasive Ventilation/ (16076)

25 ((noninvasive* or non-invasive*) adj3 ventilat*).tw,kf. (29573)

26 (NIV adj10 (noninvasive* or non-invasive* or ventilat*)).tw,kf. (8672)

27 (noninvasive PPV or non-invasive PPV or NIPPV or NPPV).tw,kf. (3792)

28 Oxygen/ and (atomi#er? or inhaler* or inhalator* or inhalation device? or nebuli* or vapo?ri*).tw,kf. (1844)

29 (oxygen* adj5 (atomi#er? or inhaler* or inhalator* or inhalation device? or nebuli* or vapo?ri*)).tw,kf. (1264)

30 ((air or gas$3) adj5 (atomi#er? or inhaler* or inhalator* or inhalation device? or nebuli* or vapo?ri*)).tw,kf. (2874)

31 FLO2max*.tw,kf. (0)

32 Bronchial Provocation Tests/ (13462)

33 ((bronchial* or endobronchial or endo-bronchial) adj3 (challenge? or provocation)).tw,kf. (7744)

34 (BP test* adj10 bronch*).tw,kf. (5)

35 ((inhalation adj3 (challenge? or provocation)) and test*).tw,kf. (2224)

36 "Nebulizers and Vaporizers"/ (20187)

37 exp Metered Dose Inhalers/ (9341)

38 (nebulize* or nebulise* or (inhal* adj3 aerosol*)).tw,kf. (37008)

39 Aerogen*.tw,kf. (8904)

40 Bronchoscopy/ (79805)

41 (bronchoscop* or broncho-scop*).tw,kf. (78324)

42 (bronch* adj3 endoscop*).tw,kf. (1986)

43 (laryngotracheobronchoscop* or laryngo-tracheobronchoscop* or laryngo-tracheo-bronchoscop* or laryngotracheo-bronchoscop* or tracheobronchoscop* or tracheo-bronchoscop*).tw,kf. (581)

44 exp Cardiopulmonary Resuscitation/ (138942)

45 ((cardiopulmonary or cardio-pulmonary or mouth-to-mouth) adj3 resuscitat*).tw,kf. (43033)

46 (CPR and (BVM or respirat* or resuscitat* or intubat*)).tw,kf. (24342)

47 (chest adj3 compress*).tw,kf. (13189)

48 (cough* adj2 assist*).tw,kf. (895)

49 (CoughAssist* or Pegaso* or Cofflator* or Cof-flator* or cough machine*).tw,kf. (211)

50 (cough* adj2 augment*).tw,kf. (228)

51 Cough/rh [Rehabilitation] (25)

52 Insufflation/ (17767)

53 ("in-exsufflator" or "in-exsufflators" or "in-exsufflation" or "in-exsufflations").tw,kf. (295)

54 (insufflat* adj1 exsufflat*).tw,kf. (499)

55 "MI-E".tw,kf. (313)

56 (breathstack* or breath-stack*).tw,kf. (181)

57 (airstack* or air-stack*).tw,kf. (151)

58 (direct* adj2 cough*).tw,kf. (184)

59 ((glossopharyn* or glosso-pharyn*) adj2 (breath* or respirat*)).tw,kf. (157)

60 (cough* adj2 flow* adj5 (improv* or increas* or enhanc* or expan* or exten*)).tw,kf. (191)

61 (respiratory muscle* adj2 (aid* or support*)).tw,kf. (112)

62 (recruit* adj2 ("lung volume" or alveolar)).tw,kf. (2389)

63 ((lung or alveolar) adj1 recruit* adj2 man?euv*).tw,kf. (1090)

64 ((LVRM or LVR) adj10 (lung or volume or recruit* or man?euv*)).tw,kf. (458)

65 Frozen Sections/is, mt [Instrumentation, Methods] (475)

66 (frozen adj2 (section* or specimen* or tissue*) adj3 (biops* or cut or cuts or cutting* or knife? or knives or shave or shaved or shaves or shaving* or microtome*)).tw,kf. (2829)

67 (cryostat* or cryomicrotome* or cryo-microtome* or freezing microtome*).tw,kf. (12326)

68 Suction/ (25121)

69 suction*.tw,kf. (50388)

70 (aspirat* adj3 mechanical*).tw,kf. (970)

71 exp High-Frequency Ventilation/ (7579)

72 ((highfrequen* or high-frequen*) adj3 (ventilat* or oscillat*)).tw,kf. (15391)

73 ((HFV or HFJV or HFOV) adj10 ventilat*).tw,kf. (3486)

74 (HFV adj3 oscillat*).tw,kf. (39)

75 Oxygen Inhalation Therapy/ (50738)

76 (("high flow" or highflow) adj5 (oxygen* or O2 or nasal* or can?ul*)).tw,kf. (9669)

77 ((HHHFO or HHFO) and (heat* or humid* or "high flow" or highflow or oxygen* or O2)).tw,kf. (2)

78 (AIRVO* or Optiflow* or Vapotherm*).tw,kf. (659)

79 ((oxygen* or O2) adj10 flush flow*).tw,kf. (2)

80 Venturi*.tw,kf. (3531)

81 ((oxygen* or O2) adj5 (nonhumid* or non-humid* or dry)).tw,kf. (1150)

82 ((non-rebreath* mask* or nonrebreath* mask* or NRBM or NRBMs) and (nasal can?ula* or nose tube? or nasal tube?)).tw,kf. (144)

83 exp Intubation/ (163859)

84 Airway Extubation/ (24006)

85 (intubat* or extubat* or detubat*).tw,kf. (207586)

86 Respiration, Artificial/ (191676)

87 (artificial* adj3 respirat*).tw,kf. (5388)

88 (manual* adj3 ventilat*).tw,kf. (2224)

89 (manual* adj3 resuscitat*).tw,kf. (741)

90 ((selfinflat* or self-inflat* or flowinflat* or flow-inflat*) adj3 resuscitat*).tw,kf. (224)

91 (T-piece adj3 resuscitat*).tw,kf. (307)

92 exp Ventilators, Mechanical/ (14562)

93 (mechanical* adj3 ventilat*).tw,kf. (165365)

94 (mechanical* adj3 resuscitat*).tw,kf. (1347)

95 (support* adj3 (ventilat* or resuscitat*)).tw,kf. (34182)

96 (nasopharyngoscop* or naso-pharyngoscop* or fiberoptic* nasendoscop* or fiber-optic* nasendoscop* or fibreoptic* nasendoscop* or fibre-optic* nasendoscop* or nasolaryngoscop* or naso-laryngoscop* or nasopharyngolaryngoscop* or naso-pharyngolaryngoscop* or (flexible adj2 laryngoscop*)).tw,kf. (3520)

97 Tracheostomy/ (36680)

98 tracheostom*.tw,kf. (40582)

99 Laryngectomy/ (21821)

100 (laryngectom* or laryn-gectom*).tw,kf. (18149)

101 Sputum/ (48810)

102 (sputum* or expectorat* or spit*).tw,kf. (288987)

103 ((supraglottic or supra-glottic) adj airway*).tw,kf. (3396)

104 laryngeal airway*.tw,kf. (676)

105 Airway Management/ and Laryngeal Masks/ (1464)

106 (LMA and (supraglottic or supra-glottic)).tw,kf. (1086)

107 (I-GEL or I-GELR or I-GELTM).tw,kf. (2287)

108 (King LT or King LTR or King LTTM).tw,kf. (89)

109 (PAxpress* or SLIPA or SLIPAR or SLIPATM).tw,kf. (178)

110 ((transsphenoid* or trans-sphenoid*) adj3 (surger* or surgical*)).tw,kf. (9471)

111 Sphenoid Bone/su [surgery] (1804)

112 Sphenoid Sinus/su [surgery] (1619)

113 Aerosol Propellants/ (2361)

114 propellant*.tw,kf. (4126)

115 (compress* adj1 (gas or gases or gasses) adj5 (administ* or deliver* or method* or treat* or therap*)).tw,kf. (78)

116 ((analg?esi* or an?esthe*) and (cryo* or freez* or frozen*) and (agent* or spray*)).tw,kf. (882)

117 (cryoan?esthesi* or cryo-an?esthesi* or cryoanalgesi* or cryo-analgesi*).tw,kf. (556)

118 (cold adj (analg?esi* or an?esthe*)).tw,kf. (127)

119 ((oral* or spray*) adj5 lidocaine*).tw,kf. (2253)

120 Perfusion Imaging/ (31105)

121 (VQ scan* or "V/Q scan*" or (ventilation adj1 perfusion adj3 scan*) or (lung adj1 perfusion adj3 scan*) or (pulmonary adj1 perfusion adj3 scan*)).tw,kf. (6390)

122 (VQ scintigraph* or "V/Q scintigraph*" or (ventilation adj1 perfusion adj3 scintigraph*) or (lung adj1 perfusion adj3 scintigraph*) or (pulmonary adj1 perfusion adj3 scintigraph*)).tw,kf. (2565)

123 (VQ scintiscan* or "V/Q scintiscan*" or (ventilation adj1 perfusion adj3 scintiscan*) or (lung adj1 perfusion adj3 scintiscan*) or (pulmonary adj1 perfusion adj3 scintiscan*)).tw,kf. (27)

124 (VQ scintigram* or "V/Q scintigram*" or (ventilation adj1 perfusion adj3 scintigram*) or (lung adj1 perfusion adj3 scintigram*) or (pulmonary adj1 perfusion adj3 scintigram*)).tw,kf. (250)

125 (VQ scintiphoto* or "V/Q scintiphoto*" or (ventilation adj1 perfusion adj3 scintiphoto*) or (lung adj1 perfusion adj3 scintiphoto*) or (pulmonary adj1 perfusion adj3 scintiphoto*)).tw,kf. (18)

126 (VQ scinti-photo* or "V/Q scinti-photo*" or (ventilation adj1 perfusion adj3 scinti-photo*) or (lung adj1 perfusion adj3 scinti-photo*) or (pulmonary adj1 perfusion adj3 scinti-photo*)).tw,kf. (0)

127 droplet?.tw,kf. (110458)

128 ((secrete* or secreti*) adj3 (breath* or cough* or mouth* or mucus* or nasal* or nose? or oral* or nasopharyn* or naso-pharyn* or oropharyn* or oro-pharyn* or pharyn* or respirat* or saliva*)).tw,kf. (38139)

129 or/1-128 [AEROSOL PROCEDURES] (1938330)

130 Cross Infection/tm [transmission] (5377)

131 (cross infection* adj5 (assistant? or employee* or personnel or professional? or staff or technician* or worker?)).tw,kf. (205)

132 ((healthcare associated infection* or health care associated infection*) adj5 (assistant? or employee* or personnel or professional? or staff or technician* or worker?)).tw,kf. (189)

133 ((healthcare associated transmi* or health care associated transmi*) adj5 (assistant? or employee* or personnel or professional? or staff or technician* or worker?)).tw,kf. (7)

134 (hospital infection* adj5 (assistant? or employee* or personnel or professional? or staff or technician* or worker?)).tw,kf. (235)

135 (nosocomial infection* adj5 (assistant? or employee* or personnel or professional? or staff or technician* or worker?)).tw,kf. (370)

136 (nosocomial adj5 transmi* adj5 (assistant? or employee* or personnel or professional? or staff or technician* or worker?)).tw,kf. (251)

137 ((disease* or infect* or viral* or virus*) adj5 risk* adj5 (assistant? or employee* or personnel or professional? or staff or technician* or worker?)).tw,kf. (7047)

138 Infectious Disease Transmission, Patient-to-Professional/ (109802)

139 (((disease* or infect* or viral* or virus*) adj3 transmi*) and (patient? adj5 (assistant? or employee* or personnel or professional? or staff or technician* or worker?))).tw,kf. (2444)

140 (((disease* or infect* or viral* or virus*) adj3 spread*) and (patient? adj5 (assistant? or employee* or personnel or professional? or staff or technician* or worker?))).tw,kf. (1176)

141 (((disease* or infect* or viral* or virus*) adj3 expos*) and (patient? adj5 (assistant? or employee* or personnel or professional? or staff or technician* or worker?))).tw,kf. (706)

142 (((disease* or infect* or viral* or virus*) adj3 contact*) and (patient? adj5 (assistant? or employee* or personnel or professional? or staff or technician* or worker?))).tw,kf. (414)

143 (((disease* or infect* or viral* or virus*) adj3 transmi*) and (patient? adj5 (allergist* or an?esthet?ist* or an?esthesia assistant* or an?esthesiologist* or audiologist* or cardiologist* or dental technician* or dentist* or dermatologist* or doctor* or emergency medical technician* or endocrinologist* or endodontist* or exodontist* or gastroenterologist* or gastro-enterologist* or general practitioner* or geriatrician* or gyn?ecologist* or hospitalist* or nephrologist* or neurologist* or nurse* or obstetrician* or oncologist* or ophthalmologist* or orthodontist* or osteopath or osteopaths or otolaryngologist* or paramedic* or pathologist* or p?ediatrician* or periodontist* or physiatrist* or physician* or physiotherapist* or physio-therapist* or prosthodontist* or pulmonologist* or radiologist* or respiratory technician* or rheumatologist* or surgeon* or therapist* or urologist*))).tw,kf. (1906)

144 (((disease* or infect* or viral* or virus*) adj3 spread*) and (patient? adj5 (allergist* or an?esthet?ist* or an?esthesia assistant* or an?esthesiologist* or audiologist* or cardiologist* or dental technician* or dentist* or dermatologist* or doctor* or emergency medical technician* or endocrinologist* or endodontist* or exodontist* or gastroenterologist* or gastro-enterologist* or general practitioner* or geriatrician* or gyn?ecologist* or hospitalist* or nephrologist* or neurologist* or nurse* or obstetrician* or oncologist* or ophthalmologist* or orthodontist* or osteopath or osteopaths or otolaryngologist* or paramedic* or pathologist* or p?ediatrician* or periodontist* or physiatrist* or physician* or physiotherapist* or physio-therapist* or prosthodontist* or pulmonologist* or radiologist* or respiratory technician* or rheumatologist* or surgeon* or therapist* or urologist*))).tw,kf. (650)

145 (((disease* or infect* or viral* or virus*) adj3 expos*) and (patient? adj5 (allergist* or an?esthet?ist* or an?esthesia assistant* or an?esthesiologist* or audiologist* or cardiologist* or dental technician* or dentist* or dermatologist* or doctor* or emergency medical technician* or endocrinologist* or endodontist* or exodontist* or gastroenterologist* or gastro-enterologist* or general practitioner* or geriatrician* or gyn?ecologist* or hospitalist* or nephrologist* or neurologist* or nurse* or obstetrician* or oncologist* or ophthalmologist* or orthodontist* or osteopath or osteopaths or otolaryngologist* or paramedic* or pathologist* or p?ediatrician* or periodontist* or physiatrist* or physician* or physiotherapist* or physio-therapist* or prosthodontist* or pulmonologist* or radiologist* or respiratory technician* or rheumatologist* or surgeon* or therapist* or urologist*))).tw,kf. (573)

146 (((disease* or infect* or viral* or virus*) adj3 contact*) and (patient? adj5 (allergist* or an?esthet?ist* or an?esthesia assistant* or an?esthesiologist* or audiologist* or cardiologist* or dental technician* or dentist* or dermatologist* or doctor* or emergency medical technician* or endocrinologist* or endodontist* or exodontist* or gastroenterologist* or gastro-enterologist* or general practitioner* or geriatrician* or gyn?ecologist* or hospitalist* or nephrologist* or neurologist* or nurse* or obstetrician* or oncologist* or ophthalmologist* or orthodontist* or osteopath or osteopaths or otolaryngologist* or paramedic* or pathologist* or p?ediatrician* or periodontist* or physiatrist* or physician* or physiotherapist* or physio-therapist* or prosthodontist* or pulmonologist* or radiologist* or respiratory technician* or rheumatologist* or surgeon* or therapist* or urologist*))).tw,kf. (300)

147 ((disease* or infect* or viral* or virus*) adj5 risk* adj5 (allergist* or an?esthet?ist* or an?esthesia assistant* or an?esthesiologist* or audiologist* or cardiologist* or dental technician* or dentist* or dermatologist* or doctor* or emergency medical technician* or endocrinologist* or endodontist* or exodontist* or gastroenterologist* or gastro-enterologist* or general practitioner* or geriatrician* or gyn?ecologist* or hospitalist* or nephrologist* or neurologist* or nurse* or obstetrician* or oncologist* or ophthalmologist* or orthodontist* or osteopath or osteopaths or otolaryngologist* or paramedic* or pathologist* or p?ediatrician* or periodontist* or physiatrist* or physician* or physiotherapist* or physio-therapist* or prosthodontist* or pulmonologist* or radiologist* or respiratory technician* or rheumatologist* or surgeon* or therapist* or urologist*)).tw,kf. (3568)

148 or/130-147 [DISEASE RISK/TRANSMISSION - PATIENT TO PROFESSIONAL] (131048)

149 129 and 148 [AEROSOL PROCEDURES - DISEASE RISK/TRANSMISSION - PATIENT TO PROFESSIONAL] (6724)

150 exp Health Personnel/ (2261940)

151 ((allied health adj3 personnel) or (allied health adj3 employee*) or (allied health adj3 staff) or (allied health adj3 worker*) or (health care adj3 assistant*) or (healthcare adj3 assistant*) or (health care adj3 employee*) or (healthcare adj3 employee*) or (health care adj3 personnel) or (healthcare adj3 personnel) or (health care adj3 staff) or (healthcare adj3 staff) or (health care adj3 worker*) or (healthcare adj3 worker*) or (health adj3 personnel) or (health adj3 employee*) or (health adj3 staff) or (health adj3 worker*) or (health adj3 assistant*) or (health care adj3 professional*) or (healthcare adj3 professional*) or (health care adj3 provider*) or (healthcare adj3 provider*) or (hospital adj3 assistant*) or (hospital adj3 employee*) or (hospital adj3 personnel) or (hospital adj3 staff) or (hospital adj3 worker*) or (medical adj3 assistant*) or (medical adj3 employee*) or (medical adj3 personnel) or (medical adj3 staff) or (medical adj3 worker*) or (nursing adj3 assistant*) or (nursing adj3 employee*) or (nursing adj3 personnel*) or (nursing adj3 staff) or (nursing adj3 worker*) or (critical care adj3 assistant*) or (critical care adj3 employee*) or (critical care adj3 personnel) or (critical care adj3 staff) or (critical care adj3 worker*) or (intensive care adj3 assistant*) or (intensive care adj3 employee*) or (intensive care adj3 personnel) or (intensive care adj3 staff) or (intensive care adj3 worker*) or (ICU adj3 assistant*) or (ICU adj3 employee*) or (ICU adj3 personnel) or (ICU adj3 staff) or (ICU adj3 worker*) or (respiratory adj3 assistant*) or (respiratory adj3 employee*) or (respiratory adj3 personnel) or (respiratory adj3 staff) or (respiratory adj3 worker*) or (emergency adj3 assistant*) or (emergency adj3 employee*) or (emergency adj3 personnel) or (emergency adj3 staff) or (emergency adj3 worker*)).tw,kf. (615222)

152 (allergist* or an?esthet?ist* or an?esthesia assistant* or an?esthesiologist* or audiologist* or cardiologist* or dental assistant* or dental personnel or dental staff or dental technician* or dentist* or dermatologist* or doctor* or emergency medical personnel or emergency medical technician* or endocrinologist* or endodontist* or exodontist* or gastroenterologist* or gastro-enterologist* or general practitioner* or geriatrician* or gyn?ecologist* or hospitalist* or nephrologist* or neurologist* or nurse* or obstetrician* or occupational therapist* or oncologist* or ophthalmologist* or orthodontist* or osteopath or osteopaths or otolaryngologist* or paramedic* or pathologist* or p?ediatrician* or periodontist* or physiatrist* or physician* or physiotherapist* or physio-therapist* or prosthodontist* or pulmonologist* or radiologist* or respiratory technician* or rheumatologist* or surgeon* or therapist* or urologist*).tw,kf. (3426180)

153 or/150-152 [HEALTHCARE WORKERS] (4835417)

154 129 and 153 [AEROSOL PROCEDURES - HEALTHCARE WORKERS] (193175)

155 Influenza, Human/ (70922)

156 exp Influenza A virus/ (62663)

157 Influenza B virus/ (6536)

158 Influenzavirus C/ (355)

159 (influenza* or flu or grippe).tw,kf. (298606)

160 (H1N1 or PH1N1 or H3N2 or AH1N1 or "A(H1N1)" or "A/H1N1" or AH3N2 or "A(H3N2)" or "A/H3N2" or H5N1).tw,kf. (62197)

161 Common Cold/ (13700)

162 (common cold or colds).tw,kf. (13963)

163 coryza.ti. (293)

164 COVID-19/ (104862)

165 SARS-CoV-2/ (101517)

166 Coronavirus/ (11706)

167 Betacoronavirus/ (41005)

168 Coronavirus Infections/ (57090)

169 (COVID-19 or COVID19).tw,kf. (307431)

170 ((coronavirus* or corona virus*) and (hubei or wuhan or beijing or shanghai)).tw,kf. (10628)

171 (wuhan adj5 virus*).tw,kf. (572)

172 (2019-nCoV or 19nCoV or 2019nCoV).tw,kf. (3366)

173 (nCoV or n-CoV or "CoV 2" or CoV2).tw,kf. (112695)

174 (SARS-CoV-2 or SARS-CoV2 or SARSCoV-2 or SARSCoV2 or SARS2 or SARS-2 or severe acute respiratory syndrome coronavirus 2).tw,kf. (114649)

175 (2019-novel CoV or Sars-coronavirus2 or Sars-coronavirus-2 or SARS-like coronavirus* or ((novel or new or nouveau) adj2 (CoV or nCoV or covid or coronavirus* or corona virus or Pandemi*2)) or (coronavirus* and pneumonia)).tw,kf. (40324)

176 (novel coronavirus* or novel corona virus* or novel CoV).tw,kf. (20025)

177 ((coronavirus* or corona virus*) adj2 "2019").tw,kf. (68382)

178 ((coronavirus* or corona virus*) adj2 "19").tw,kf. (10949)

179 ("coronavirus 2" or "corona virus 2").tw,kf. (35862)

180 (OC43 or NL63 or 229E or HKU1 or HCoV* or Sars-coronavirus*).tw,kf. (7954)

181 COVID-19.rx,px,ox. or severe acute respiratory syndrome coronavirus 2.os. (8390)

182 (coronavirus* or corona virus*).ti. (47697)

183 COVID.ti. (246259)

184 ("B.1.1.7" or "B.1.351" or "B.1.617" or "B.1.427" or "B.1.429").tw,kf,rx,px,ox. (1255)

185 ("P.1" and (Brazil* or variant?)).tw,kf,rx,px,ox. (3695)

186 (((alpha or beta or delta or eta or gamma or iota or kappa or lambda) adj3 variant?) and (coronavirus* or corona virus* or covid*)).tw,kf. (241)

187 SARS Virus/ (10760)

188 Severe Acute Respiratory Syndrome/ (16143)

189 ((severe acute or acute severe) adj2 respiratory syndrome*).tw,kf. (48808)

190 SARS.tw,kf. (131929)

191 Middle East Respiratory Syndrome Coronavirus/ (5547)

192 (middle east adj2 respiratory syndrome*).tw,kf. (6666)

193 MERS.tw,kf. (14158)

194 Pneumovirus Infections/ (281)

195 (pneumovirus* or pneumo-virus*).tw,kf. (838)

196 Respiratory Syncytial Virus Infections/ (10910)

197 Respiratory Syncytial Viruses/ or Respiratory Syncytial Virus, Human/ (15129)

198 (respiratory adj1 sync#tial adj2 (virus* or pneumovir* or pneumo-vir*)).tw,kf. (33604)

199 (HRSV or HRSVs or RSV or RSVs or sync#tial virus* or sync#tial pneumovirus* or sync#tial pneumo-virus*).tw,kf. (44607)

200 (parainfluenza* or para-influenza*).tw,kf. (14777)

201 Respiratory Tract Infections/ (67150)

202 (respiratory adj3 (infectio* or illness* or virus*)).tw,kf. (199995)

203 ((ARI or ARIs) adj5 (acute or respirator* or infect* or illness*)).tw,kf. (4560)

204 (RTI or RTIs or URTI or URTIs or LRTI or LRTIs).tw,kf. (15108)

205 Pneumonia, Viral/ (63969)

206 (pneumonia* adj3 (viral* or virus*)).tw,kf. (10212)

207 or/155-206 [RESPIRATORY VIRAL INFECTIONS] (930021)

208 154 and 207 [AEROSOL PROCEDURES - HEALTHCARE WORKERS - RESPIRATORY VIRAL INFECTIONS] (13039)

209 Cross Infection/tm [transmission] (5377)

210 Disease Transmission, Infectious/ (115345)

211 Infectious Disease Transmission, Patient-to-Professional/ (109802)

212 Influenza, Human/tm [transmission] (3426)

213 Common Cold/tm [transmission] (106)

214 COVID-19/tm [transmission] (3680)

215 Coronavirus Infections/tm [transmission] (4704)

216 Severe Acute Respiratory Syndrome/tm [transmission] (934)

217 Pneumovirus Infections/tm [transmission] (6)

218 Respiratory Syncytial Virus Infections/tm [transmission] (151)

219 Respiratory Tract Infections/tm [transmission] (587)

220 Pneumonia, Viral/tm [transmission] (4203)

221 ((disease* or infect* or viral* or virus*) adj5 transmi*).tw,kf. (257639)

222 ((Influenza* or flu or flus or grippe or common cold* or colds or COVID or COVID-19 or COVID19 or coronavirus* or corona virus* or 2019-nCoV or 19nCoV or 2019nCoV or nCoV or n-CoV or "CoV 2" or CoV2 or SARS-CoV-2 or SARS-CoV2 or SARSCoV-2 or SARSCoV2 or SARS2 or SARS-2 or SARS or MERS or respiratory syndrome* or pneumovirus* or pneumo-virus* or HRSV or HRSVs or RSV or RSVs or sync#tial virus* or sync#tial pneumovirus* or sync#tial pneumo-virus* or parainfluenza* or para-influenza* or RTI or RTIs or URTI or URTIs or LRTI or LRTIs or viral pneumonia* or pneumonia virus* or (respiratory adj2 infection*) or (respiratory adj2 virus*) or (respiratory adj2 illness*)) adj5 transmi*).tw,kf. (23729)

223 ((disease* or infect* or viral* or virus*) adj5 spread*).tw,kf. (97032)

224 ((Influenza* or flu or flus or grippe or common cold* or colds or COVID or COVID-19 or COVID19 or coronavirus* or corona virus* or 2019-nCoV or 19nCoV or 2019nCoV or nCoV or n-CoV or "CoV 2" or CoV2 or SARS-CoV-2 or SARS-CoV2 or SARSCoV-2 or SARSCoV2 or SARS2 or SARS-2 or SARS or MERS or respiratory syndrome* or pneumovirus* or pneumo-virus* or HRSV or HRSVs or RSV or RSVs or sync#tial virus* or sync#tial pneumovirus* or sync#tial pneumo-virus* or parainfluenza* or para-influenza* or RTI or RTIs or URTI or URTIs or LRTI or LRTIs or viral pneumonia* or pneumonia virus* or (respiratory adj2 infection*) or (respiratory adj2 virus*) or (respiratory adj2 illness*)) adj5 spread*).tw,kf. (25014)

225 ((disease* or infect* or viral* or virus*) adj5 expos*).tw,kf. (110968)

226 ((Influenza* or flu or flus or grippe or common cold* or colds or COVID or COVID-19 or COVID19 or coronavirus* or corona virus* or 2019-nCoV or 19nCoV or 2019nCoV or nCoV or n-CoV or "CoV 2" or CoV2 or SARS-CoV-2 or SARS-CoV2 or SARSCoV-2 or SARSCoV2 or SARS2 or SARS-2 or SARS or MERS or respiratory syndrome* or pneumovirus* or pneumo-virus* or HRSV or HRSVs or RSV or RSVs or sync#tial virus* or sync#tial pneumovirus* or sync#tial pneumo-virus* or parainfluenza* or para-influenza* or RTI or RTIs or URTI or URTIs or LRTI or LRTIs or viral pneumonia* or pneumonia virus* or (respiratory adj2 infection*) or (respiratory adj2 virus*) or (respiratory adj2 illness*)) adj5 expos*).tw,kf. (13169)

227 ((disease* or infect* or viral* or virus*) adj5 contact*).tw,kf. (32265)

228 ((Influenza* or flu or flus or grippe or common cold* or colds or COVID or COVID-19 or COVID19 or coronavirus* or corona virus* or 2019-nCoV or 19nCoV or 2019nCoV or nCoV or n-CoV or "CoV 2" or CoV2 or SARS-CoV-2 or SARS-CoV2 or SARSCoV-2 or SARSCoV2 or SARS2 or SARS-2 or SARS or MERS or respiratory syndrome* or pneumovirus* or pneumo-virus* or HRSV or HRSVs or RSV or RSVs or sync#tial virus* or sync#tial pneumovirus* or sync#tial pneumo-virus* or parainfluenza* or para-influenza* or RTI or RTIs or URTI or URTIs or LRTI or LRTIs or viral pneumonia* or pneumonia virus* or (respiratory adj2 infection*) or (respiratory adj2 virus*) or (respiratory adj2 illness*)) adj5 contact*).tw,kf. (5448)

229 Occupational Exposure/ (142940)

230 ((health care or healthcare or hospital* or occupation* or employee* or personnel* or procedur* or staff* or work*) adj5 (contact* or expos*)).tw,kf. (214315)

231 Air Microbiology/ (262736)

232 (air adj3 (microbiol* or micro-biol* or transmi*)).tw,kf. (1866)

233 (risk* adj5 (contact* or expos* or spread* or transmi*)).tw,kf. (218373)

234 or/209-233 [DISEASE TRANSMISSION/EXPOSURE] (1238684)

235 208 and 234 [AEROSOL PROCEDURES - HEALTHCARE WORKERS - RESPIRATORY VIRAL INFECTIONS - DISEASE TRANSMISSION Pt 1] (4604)

236 149 and 207 [AEROSOL PROCEDURES - HEALTHCARE WORKERS - RESPIRATORY VIRAL INFECTIONS - DISEASE TRANSMISSION Pt 2] (2814)

237 235 or 236 [AEROSOL PROCEDURES - HEALTHCARE WORKERS - RESPIRATORY VIRAL INFECTIONS - DISEASE TRANSMISSION - COMBINED] (5767)

238 exp Animals/ not Humans/ (17508743)

239 237 not 238 [ANIMAL-ONLY REMOVED] (4602)

240 239 use ppez [MEDLINE RECORDS] (2085)

241 aerosol/ (90495)

242 aerosol generating procedure/ (682)

243 (aerosoli#e* or aerosoli#ing).tw,kw. (17926)

244 (aerosol* adj3 (contact* or dispers* or expos* or generat* or procedure* or produc* or spread* or transmi*)).tw,kw. (20767)

245 ((AGP or AGPs or AGMP or AGMPs or AGDP or AGDPs) and aerosol*).tw,kw. (311)

246 (aerosol* adj5 (clinic* or dent* or medic* or therap* or treatment*)).tw,kw. (11548)

247 (bioaerosol* or bio-aerosol*).tw,kw. (4767)

248 ("3-in-1" adj2 syringe?).tw,kw. (29)

249 (water adj1 air? adj2 syringe?).tw,kw. (230)

250 ((air rota or air rotar*) and dent*).tw,kw. (1)

251 dental polishing device/ (28)

252 (((polish* or rotar* or finishing or handpiece* or hand piece*) adj3 (device* or equipment* or instrument*)) and dent*).tw,kw. (2113)

253 ((ultrasonic* or sonic*) adj3 (scale* or scaling)).tw,kw. (2017)

254 (air polish* or air abrasion* or air turbine handpiece? or air turbine hand piece?).tw,kw. (2113)

255 (dent* adj3 (handpiece* or hand piece*) adj5 (highspeed* or high speed* or lowspeed* or low speed*)).tw,kw. (160)

256 autopsy/ (195259)

257 (autops* or postmortem exam* or post-mortem exam*).tw,kw. (198178)

258 manual emergency ventilator/ (672)

259 bag-valve*.tw,kw. (1679)

260 (BVM adj10 (bag? or valve? or mask*)).tw,kw. (392)

261 (ambu bag? or bag-mask? or (bag? adj1 resuscitat*) or (manual* adj1 resuscitat?) or self-inflating bag? or selfinflating bag? or "Revivator-Plus").tw,kw. (2530)

262 exp positive pressure ventilation/ (40077)

263 ((positive end-expiratory or (positive adj2 pressure)) adj (breathing or ventilat*)).tw,kw. (19061)

264 positive airway pressure.tw,kw. (36154)

265 (PEEP and (positive or expiratory or pressure)).tw,kw. (14465)

266 (APRV or BiPAP or BPAP or CPCP or nCPAP or CPPB or CPPV or IPPB or IPPV).tw,kw. (11390)

267 exp noninvasive ventilation/ (18524)

268 ((noninvasive* or non-invasive*) adj3 ventilat*).tw,kw. (30089)

269 (NIV adj10 (noninvasive* or non-invasive* or ventilat*)).tw,kw. (8772)

270 (noninvasive PPV or non-invasive PPV or NIPPV or NPPV).tw,kw. (3824)

271 oxygen/ and (atomi#er? or inhaler* or inhalator* or inhalation device? or nebuli* or vapo?ri*).tw,kw. (1859)

272 (oxygen* adj5 (atomi#er? or inhaler* or inhalator* or inhalation device? or nebuli* or vapo?ri*)).tw,kw. (1360)

273 ((air or gas$3) adj5 (atomi#er? or inhaler* or inhalator* or inhalation device? or nebuli* or vapo?ri*)).tw,kw. (2888)

274 FLO2max*.tw,kw. (0)

275 inhalation test/ (3380)

276 ((bronchial* or endobronchial or endo-bronchial) adj3 (challenge? or provocation)).tw,kw. (8010)

277 (BP test* adj10 bronch*).tw,kw. (5)

278 ((inhalation adj3 (challenge? or provocation)) and test*).tw,kw. (2280)

279 exp nebulizer/ (25841)

280 exp metered dose inhaler/ (9341)

281 (nebulize* or nebulise* or (inhal* adj3 aerosol*)).tw,kw. (37661)

282 Aerogen*.tw,kw. (8630)

283 exp bronchoscopy/ (89296)

284 (bronchoscop* or broncho-scop*).tw,kw. (80200)

285 (bronch* adj3 endoscop*).tw,kw. (2128)

286 (laryngotracheobronchoscop* or laryngo-tracheobronchoscop* or laryngo-tracheo-bronchoscop* or laryngotracheo-bronchoscop* or tracheobronchoscop* or tracheo-bronchoscop*).tw,kw. (592)

287 exp resuscitation/ (224613)

288 ((cardiopulmonary or cardio-pulmonary or mouth-to-mouth) adj3 resuscitat*).tw,kw. (44745)

289 (CPR and (BVM or respirat* or resuscitat* or intubat*)).tw,kw. (25151)

290 (chest adj3 compress*).tw,kw. (13279)

291 non continuous ventilator/ (137)

292 (cough* adj2 assist*).tw,kw. (913)

293 (CoughAssist* or Pegaso* or Cofflator* or Cof-flator* or cough machine*).tw,kw. (215)

294 (cough* adj2 augment*).tw,kw. (233)

295 exp coughing/rh [Rehabilitation] (14)

296 aeration/ (13900)

297 ("in-exsufflator" or "in-exsufflators" or "in-exsufflation" or "in-exsufflations").tw,kw. (299)

298 (insufflat* adj1 exsufflat*).tw,kw. (515)

299 "MI-E".tw,kw. (312)

300 (breathstack* or breath-stack*).tw,kw. (183)

301 (airstack* or air-stack*).tw,kw. (151)

302 (direct* adj2 cough*).tw,kw. (187)

303 ((glossopharyn* or glosso-pharyn*) adj2 (breath* or respirat*)).tw,kw. (171)

304 (cough* adj2 flow* adj5 (improv* or increas* or enhanc* or expan* or exten*)).tw,kw. (191)

305 (respiratory muscle* adj2 (aid* or support*)).tw,kw. (112)

306 (recruit* adj2 ("lung volume" or alveolar)).tw,kw. (2443)

307 ((lung or alveolar) adj1 recruit* adj2 man?euv*).tw,kw. (1101)

308 ((LVRM or LVR) adj10 (lung or volume or recruit* or man?euv*)).tw,kw. (459)

309 frozen section/ and (biops* or cut or cuts or cutting* or knife? or knives or shave or shaved or shaves or shaving* or microtome*).tw,kw. (5686)

310 (frozen adj2 (section* or specimen* or tissue*) adj3 (biops* or cut or cuts or cutting* or knife? or knives or shave or shaved or shaves or shaving* or microtome*)).tw,kw. (2838)

311 (cryostat* or cryomicrotome* or cryo-microtome* or freezing microtome*).tw,kw. (12352)

312 suction/ (25121)

313 suction*.tw,kw. (50742)

314 (aspirat* adj3 mechanical*).tw,kw. (1021)

315 high frequency ventilation/ (6018)

316 ((highfrequen* or high-frequen*) adj3 (ventilat* or oscillat*)).tw,kw. (15593)

317 ((HFV or HFJV or HFOV) adj10 ventilat*).tw,kw. (3507)

318 (HFV adj3 oscillat*).tw,kw. (39)

319 exp oxygen therapy/ (82223)

320 (("high flow" or highflow) adj5 (oxygen* or O2 or nasal* or can?ul*)).tw,kw. (9696)

321 ((HHHFO or HHFO) and (heat* or humid* or "high flow" or highflow or oxygen* or O2)).tw,kw. (2)

322 (AIRVO* or Optiflow* or Vapotherm*).tw,kw. (666)

323 ((oxygen* or O2) adj10 flush flow*).tw,kw. (2)

324 Venturi*.tw,kw. (3559)

325 ((oxygen* or O2) adj5 (nonhumid* or non-humid* or dry)).tw,kw. (1151)

326 ((non-rebreath* mask* or nonrebreath* mask* or NRBM or NRBMs) and (nasal can?ula* or nose tube? or nasal tube?)).tw,kw. (145)

327 exp intubation/ (163859)

328 extubation/ (21839)

329 (intubat* or extubat* or detubat*).tw,kw. (209514)

330 artificial ventilation/ (149809)

331 (artificial* adj3 respirat*).tw,kw. (5307)

332 (manual* adj3 ventilat*).tw,kw. (2324)

333 (manual* adj3 resuscitat*).tw,kw. (749)

334 ((selfinflat* or self-inflat* or flowinflat* or flow-inflat*) adj3 resuscitat*).tw,kw. (232)

335 (T-piece adj3 resuscitat*).tw,kw. (308)

336 mechanical ventilator/ (14181)

337 (mechanical* adj3 ventilat*).tw,kw. (167741)

338 (mechanical* adj3 resuscitat*).tw,kw. (1382)

339 (support* adj3 (ventilat* or resuscitat*)).tw,kw. (34374)

340 (nasopharyngoscop* or naso-pharyngoscop* or fiberoptic* nasendoscop* or fiber-optic* nasendoscop* or fibreoptic* nasendoscop* or fibre-optic* nasendoscop* or nasolaryngoscop* or naso-laryngoscop* or nasopharyngolaryngoscop* or naso-pharyngolaryngoscop* or (flexible adj2 laryngoscop*)).tw,kw. (3567)

341 tracheostomy/ (36680)

342 tracheostom*.tw,kw. (41150)

343 laryngectomy/ (21821)

344 (laryngectom* or laryn-gectom*).tw,kw. (18382)

345 sputum/ (48810)

346 (sputum* or expectorat* or spit*).tw,kw. (289543)

347 supraglottic airway device/ (1492)

348 ((supraglottic or supra-glottic) adj airway*).tw,kw. (3478)

349 laryngeal airway*.tw,kw. (681)

350 respiration control/ and laryngeal mask/ (1033)

351 (LMA and (supraglottic or supra-glottic)).tw,kw. (1109)

352 (I-GEL or I-GELR or I-GELTM).tw,kw. (2296)

353 (King LT or King LTR or King LTTM).tw,kw. (89)

354 (PAxpress* or SLIPA or SLIPAR or SLIPATM).tw,kw. (178)

355 ((transsphenoid* or trans-sphenoid*) adj3 (surger* or surgical*)).tw,kw. (9807)

356 sphenoid/su [surgery] (207)

357 sphenoid sinus/su [surgery] (1619)

358 propellant/ (1527)

359 propellant*.tw,kw. (4183)

360 (compress* adj1 (gas or gases or gasses) adj5 (administ* or deliver* or method* or treat* or therap*)).tw,kw. (78)

361 cryoanesthesia/ (2800)

362 ((analg?esi* or an?esthe*) and (cryo* or freez* or frozen*) and (agent* or spray*)).tw,kw. (917)

363 (cryoan?esthesi* or cryo-an?esthesi* or cryoanalgesi* or cryo-analgesi*).tw,kw. (618)

364 (cold adj (analg?esi* or an?esthe*)).tw,kw. (106)

365 ((oral* or spray*) adj5 lidocaine*).tw,kw. (2271)

366 ventilation-perfusion scan/ (430)

367 (VQ scan* or "V/Q scan*" or (ventilation adj1 perfusion adj3 scan*) or (lung adj1 perfusion adj3 scan*) or (pulmonary adj1 perfusion adj3 scan*)).tw,kw. (6454)

368 (VQ scintigraph* or "V/Q scintigraph*" or (ventilation adj1 perfusion adj3 scintigraph*) or (lung adj1 perfusion adj3 scintigraph*) or (pulmonary adj1 perfusion adj3 scintigraph*)).tw,kw. (2597)

369 (VQ scintiscan* or "V/Q scintiscan*" or (ventilation adj1 perfusion adj3 scintiscan*) or (lung adj1 perfusion adj3 scintiscan*) or (pulmonary adj1 perfusion adj3 scintiscan*)).tw,kw. (38)

370 (VQ scintigram* or "V/Q scintigram*" or (ventilation adj1 perfusion adj3 scintigram*) or (lung adj1 perfusion adj3 scintigram*) or (pulmonary adj1 perfusion adj3 scintigram*)).tw,kw. (251)

371 (VQ scintiphoto* or "V/Q scintiphoto*" or (ventilation adj1 perfusion adj3 scintiphoto*) or (lung adj1 perfusion adj3 scintiphoto*) or (pulmonary adj1 perfusion adj3 scintiphoto*)).tw,kw. (18)

372 (VQ scinti-photo* or "V/Q scinti-photo*" or (ventilation adj1 perfusion adj3 scinti-photo*) or (lung adj1 perfusion adj3 scinti-photo*) or (pulmonary adj1 perfusion adj3 scinti-photo*)).tw,kw. (0)

373 droplet?.tw,kw. (110695)

374 ((secrete* or secreti*) adj3 (breath* or cough* or mouth* or mucus* or nasal* or nose? or oral* or nasopharyn* or naso-pharyn* or oropharyn* or oro-pharyn* or pharyn* or respirat* or saliva*)).tw,kw. (38377)

375 or/241-374 [AEROSOL PROCEDURES] (1966072)

376 (cross infection* adj5 (assistant? or employee* or personnel or professional? or staff or technician* or worker?)).tw,kw. (209)

377 ((healthcare associated infection* or health care associated infection*) adj5 (assistant? or employee* or personnel or professional? or staff or technician* or worker?)).tw,kw. (209)

378 ((healthcare associated transmi* or health care associated transmi*) adj5 (assistant? or employee* or personnel or professional? or staff or technician* or worker?)).tw,kw. (6)

379 (hospital infection* adj5 (assistant? or employee* or personnel or professional? or staff or technician* or worker?)).tw,kw. (266)

380 (nosocomial infection* adj5 (assistant? or employee* or personnel or professional? or staff or technician* or worker?)).tw,kw. (383)

381 (nosocomial adj5 transmi* adj5 (assistant? or employee* or personnel or professional? or staff or technician* or worker?)).tw,kw. (256)

382 ((disease* or infect* or viral* or virus*) adj5 risk* adj5 (assistant? or employee* or personnel or professional? or staff or technician* or worker?)).tw,kw. (7104)

383 (((disease* or infect* or viral* or virus*) adj3 transmi*) and (patient? adj5 (assistant? or employee* or personnel or professional? or staff or technician* or worker?))).tw,kw. (2477)

384 (((disease* or infect* or viral* or virus*) adj3 spread*) and (patient? adj5 (assistant? or employee* or personnel or professional? or staff or technician* or worker?))).tw,kw. (1171)

385 (((disease* or infect* or viral* or virus*) adj3 expos*) and (patient? adj5 (assistant? or employee* or personnel or professional? or staff or technician* or worker?))).tw,kw. (712)

386 (((disease* or infect* or viral* or virus*) adj3 contact*) and (patient? adj5 (assistant? or employee* or personnel or professional? or staff or technician* or worker?))).tw,kw. (428)

387 (((disease* or infect* or viral* or virus*) adj3 transmi*) and (patient? adj5 (allergist* or an?esthet?ist* or an?esthesia assistant* or an?esthesiologist* or audiologist* or cardiologist* or dental technician* or dentist* or dermatologist* or doctor* or emergency medical technician* or endocrinologist* or endodontist* or exodontist* or gastroenterologist* or gastro-enterologist* or general practitioner* or geriatrician* or gyn?ecologist* or hospitalist* or nephrologist* or neurologist* or nurse* or obstetrician* or oncologist* or ophthalmologist* or orthodontist* or osteopath or osteopaths or otolaryngologist* or paramedic* or pathologist* or p?ediatrician* or periodontist* or physiatrist* or physician* or physiotherapist* or physio-therapist* or prosthodontist* or pulmonologist* or radiologist* or respiratory technician* or rheumatologist* or surgeon* or therapist* or urologist*))).tw,kw. (1933)

388 (((disease* or infect* or viral* or virus*) adj3 spread*) and (patient? adj5 (allergist* or an?esthet?ist* or an?esthesia assistant* or an?esthesiologist* or audiologist* or cardiologist* or dental technician* or dentist* or dermatologist* or doctor* or emergency medical technician* or endocrinologist* or endodontist* or exodontist* or gastroenterologist* or gastro-enterologist* or general practitioner* or geriatrician* or gyn?ecologist* or hospitalist* or nephrologist* or neurologist* or nurse* or obstetrician* or oncologist* or ophthalmologist* or orthodontist* or osteopath or osteopaths or otolaryngologist* or paramedic* or pathologist* or p?ediatrician* or periodontist* or physiatrist* or physician* or physiotherapist* or physio-therapist* or prosthodontist* or pulmonologist* or radiologist* or respiratory technician* or rheumatologist* or surgeon* or therapist* or urologist*))).tw,kw. (656)

389 (((disease* or infect* or viral* or virus*) adj3 expos*) and (patient? adj5 (allergist* or an?esthet?ist* or an?esthesia assistant* or an?esthesiologist* or audiologist* or cardiologist* or dental technician* or dentist* or dermatologist* or doctor* or emergency medical technician* or endocrinologist* or endodontist* or exodontist* or gastroenterologist* or gastro-enterologist* or general practitioner* or geriatrician* or gyn?ecologist* or hospitalist* or nephrologist* or neurologist* or nurse* or obstetrician* or oncologist* or ophthalmologist* or orthodontist* or osteopath or osteopaths or otolaryngologist* or paramedic* or pathologist* or p?ediatrician* or periodontist* or physiatrist* or physician* or physiotherapist* or physio-therapist* or prosthodontist* or pulmonologist* or radiologist* or respiratory technician* or rheumatologist* or surgeon* or therapist* or urologist*))).tw,kw. (603)

390 (((disease* or infect* or viral* or virus*) adj3 contact*) and (patient? adj5 (allergist* or an?esthet?ist* or an?esthesia assistant* or an?esthesiologist* or audiologist* or cardiologist* or dental technician* or dentist* or dermatologist* or doctor* or emergency medical technician* or endocrinologist* or endodontist* or exodontist* or gastroenterologist* or gastro-enterologist* or general practitioner* or geriatrician* or gyn?ecologist* or hospitalist* or nephrologist* or neurologist* or nurse* or obstetrician* or oncologist* or ophthalmologist* or orthodontist* or osteopath or osteopaths or otolaryngologist* or paramedic* or pathologist* or p?ediatrician* or periodontist* or physiatrist* or physician* or physiotherapist* or physio-therapist* or prosthodontist* or pulmonologist* or radiologist* or respiratory technician* or rheumatologist* or surgeon* or therapist* or urologist*))).tw,kw. (312)

391 ((disease* or infect* or viral* or virus*) adj5 risk* adj5 (allergist* or an?esthet?ist* or an?esthesia assistant* or an?esthesiologist* or audiologist* or cardiologist* or dental technician* or dentist* or dermatologist* or doctor* or emergency medical technician* or endocrinologist* or endodontist* or exodontist* or gastroenterologist* or gastro-enterologist* or general practitioner* or geriatrician* or gyn?ecologist* or hospitalist* or nephrologist* or neurologist* or nurse* or obstetrician* or oncologist* or ophthalmologist* or orthodontist* or osteopath or osteopaths or otolaryngologist* or paramedic* or pathologist* or p?ediatrician* or periodontist* or physiatrist* or physician* or physiotherapist* or physio-therapist* or prosthodontist* or pulmonologist* or radiologist* or respiratory technician* or rheumatologist* or surgeon* or therapist* or urologist*)).tw,kw. (3758)

392 or/376-391 [DISEASE RISK/TRANSMISSION - PATIENT TO PROFESSIONAL] (18502)

393 375 and 392 [AEROSOL PROCEDURES - DISEASE RISK/TRANSMISSION - PATIENT TO PROFESSIONAL] (1829)

394 exp health care personnel/ (1698416)

395 ((allied health adj3 assistant*) or (allied health adj3 personnel) or (allied health adj3 employee*) or (allied health adj3 staff) or (allied health adj3 worker*) or (health care adj3 assistant*) or (healthcare adj3 assistant*) or (health care adj3 employee*) or (healthcare adj3 employee*) or (health care adj3 personnel) or (healthcare adj3 personnel) or (health care adj3 staff) or (healthcare adj3 staff) or (health care adj3 worker*) or (healthcare adj3 worker*) or (health adj3 personnel) or (health adj3 employee*) or (health adj3 staff) or (health adj3 worker*) or (health adj3 assistant*) or (health care adj3 professional*) or (healthcare adj3 professional*) or (health care adj3 provider*) or (healthcare adj3 provider*) or (hospital adj3 assistant*) or (hospital adj3 employee*) or (hospital adj3 personnel) or (hospital adj3 staff) or (hospital adj3 worker*) or (medical adj3 assistant*) or (medical adj3 employee*) or (medical adj3 personnel) or (medical adj3 staff) or (medical adj3 worker*) or (nursing adj3 assistant*) or (nursing adj3 employee*) or (nursing adj3 personnel*) or (nursing adj3 staff) or (nursing adj3 worker*) or (critical care adj3 assistant*) or (critical care adj3 employee*) or (critical care adj3 personnel) or (critical care adj3 staff) or (critical care adj3 worker*) or (intensive care adj3 assistant*) or (intensive care adj3 employee*) or (intensive care adj3 personnel) or (intensive care adj3 staff) or (intensive care adj3 worker*) or (ICU adj3 assistant*) or (ICU adj3 employee*) or (ICU adj3 personnel) or (ICU adj3 staff) or (ICU adj3 worker*) or (respiratory adj3 assistant*) or (respiratory adj3 employee*) or (respiratory adj3 personnel) or (respiratory adj3 staff) or (respiratory adj3 worker*) or (emergency adj3 assistant*) or (emergency adj3 employee*) or (emergency adj3 personnel) or (emergency adj3 staff) or (emergency adj3 worker*)).tw,kw. (616696)

396 (allergist* or an?esthet?ist* or an?esthesia assistant* or an?esthesiologist* or audiologist* or cardiologist* or dental assistant* or dental personnel or dental staff or dental technician* or dentist* or dermatologist* or doctor* or emergency medical personnel or emergency medical technician* or endocrinologist* or endodontist* or exodontist* or gastroenterologist* or gastro-enterologist* or general practitioner* or geriatrician* or gyn?ecologist* or hospitalist* or nephrologist* or neurologist* or nurse* or obstetrician* or occupational therapist* or oncologist* or ophthalmologist* or orthodontist* or osteopath or osteopaths or otolaryngologist* or paramedic* or pathologist* or p?ediatrician* or periodontist* or physiatrist* or physician* or physiotherapist* or physio-therapist* or prosthodontist* or pulmonologist* or radiologist* or respiratory technician* or rheumatologist* or surgeon* or therapist* or urologist*).tw,kw. (3440607)

397 or/394-396 [HEALTHCARE WORKERS] (4577666)

398 375 and 397 [AEROSOL PROCEDURES - HEALTHCARE WORKERS] (195106)

399 influenza/ (118967)

400 exp Influenza A virus/ (62663)

401 exp Influenzavirus B/ (6733)

402 Influenzavirus C/ (355)

403 (influenza* or flu or grippe).tw,kw. (300704)

404 (H1N1 or PH1N1 or H3N2 or AH1N1 or "A(H1N1)" or "A/H1N1" or AH3N2 or "A(H3N2)" or "A/H3N2" or H5N1).tw,kw. (62518)

405 common cold/ (13700)

406 (common cold or colds).tw,kw. (14319)

407 coryza.ti. (293)

408 coronavirus disease 2019/ (247899)

409 severe acute respiratory syndrome coronavirus 2/ (122415)

410 Coronavirinae/ (5163)

411 Betacoronavirus/ (41005)

412 coronavirus infection/ (58342)

413 (COVID-19 or COVID19).tw,kw. (312619)

414 ((coronavirus* or corona virus*) and (hubei or wuhan or beijing or shanghai)).tw,kw. (10798)

415 (wuhan adj5 virus*).tw,kw. (595)

416 (2019-nCoV or 19nCoV or 2019nCoV).tw,kw. (3721)

417 (nCoV or n-CoV or "CoV 2" or CoV2).tw,kw. (112166)

418 (SARS-CoV-2 or SARS-CoV2 or SARSCoV-2 or SARSCoV2 or SARS2 or SARS-2 or severe acute respiratory syndrome coronavirus 2).tw,kw. (121811)

419 (2019-novel CoV or Sars-coronavirus2 or Sars-coronavirus-2 or SARS-like coronavirus* or ((novel or new or nouveau) adj2 (CoV or nCoV or covid or coronavirus* or corona virus or Pandemi*2)) or (coronavirus* and pneumonia)).tw,kw. (40967)

420 (novel coronavirus* or novel corona virus* or novel CoV).tw,kw. (20388)

421 ((coronavirus* or corona virus*) adj2 "2019").tw,kw. (69494)

422 ((coronavirus* or corona virus*) adj2 "19").tw,kw. (10615)

423 ("coronavirus 2" or "corona virus 2").tw,kw. (35868)

424 (OC43 or NL63 or 229E or HKU1 or HCoV* or Sars-coronavirus*).tw,kw. (8155)

425 (coronavirus* or corona virus*).ti. (47697)

426 COVID.ti. (246259)

427 ("B.1.1.7" or "B.1.351" or "B.1.617" or "B.1.427" or "B.1.429").tw,kw. (1263)

428 ("P.1" and (Brazil* or variant?)).tw,kw. (3680)

429 (((alpha or beta or delta or eta or gamma or iota or kappa or lambda) adj3 variant?) and (coronavirus* or corona virus* or covid*)).tw,kw. (248)

430 SARS coronavirus/ (10760)

431 severe acute respiratory syndrome/ (16143)

432 ((severe acute or acute severe) adj2 respiratory syndrome*).tw,kw. (48946)

433 SARS.tw,kw. (131758)

434 exp Middle East respiratory syndrome coronavirus/ (5546)

435 (middle east adj2 respiratory syndrome*).tw,kw. (6604)

436 MERS.tw,kw. (14192)

437 Pneumovirus/ (349)

438 (pneumovirus* or pneumo-virus*).tw,kw. (895)

439 respiratory syncytial virus infection/ (14002)

440 Human respiratory syncytial virus/ (8800)

441 (respiratory adj1 sync#tial adj2 (virus* or pneumovir* or pneumo-vir*)).tw,kw. (33865)

442 (HRSV or HRSVs or RSV or RSVs or sync#tial virus* or sync#tial pneumovirus* or sync#tial pneumo-virus*).tw,kw. (44895)

443 (parainfluenza* or para-influenza*).tw,kw. (14863)

444 respiratory tract infection/ (103474)

445 (respiratory adj3 (infectio* or illness* or virus*)).tw,kw. (200949)

446 ((ARI or ARIs) adj5 (acute or respirator* or infect* or illness*)).tw,kw. (4570)

447 (RTI or RTIs or URTI or URTIs or LRTI or LRTIs).tw,kw. (15197)

448 exp virus pneumonia/ (29191)

449 (pneumonia* adj3 (viral* or virus*)).tw,kw. (10463)

450 or/399-449 [RESPIRATORY VIRAL INFECTIONS] (967385)

451 398 and 450 [AEROSOL PROCEDURES - HEALTHCARE WORKERS - RESPIRATORY VIRAL INFECTIONS] (13686)

452 disease transmission/ (104472)

453 virus transmission/ (73213)

454 ((disease* or infect* or viral* or virus*) adj5 transmi*).tw,kw. (259114)

455 ((Influenza* or flu or flus or grippe or common cold* or colds or COVID or COVID-19 or COVID19 or coronavirus* or corona virus* or 2019-nCoV or 19nCoV or 2019nCoV or nCoV or n-CoV or "CoV 2" or CoV2 or SARS-CoV-2 or SARS-CoV2 or SARSCoV-2 or SARSCoV2 or SARS2 or SARS-2 or SARS or MERS or respiratory syndrome* or pneumovirus* or pneumo-virus* or HRSV or HRSVs or RSV or RSVs or sync#tial virus* or sync#tial pneumovirus* or sync#tial pneumo-virus* or parainfluenza* or para-influenza* or RTI or RTIs or URTI or URTIs or LRTI or LRTIs or viral pneumonia* or pneumonia virus* or (respiratory adj2 infection*) or (respiratory adj2 virus*) or (respiratory adj2 illness*)) adj5 transmi*).tw,kw. (24292)

456 ((disease* or infect* or viral* or virus*) adj5 spread*).tw,kw. (97083)

457 ((Influenza* or flu or flus or grippe or common cold* or colds or COVID or COVID-19 or COVID19 or coronavirus* or corona virus* or 2019-nCoV or 19nCoV or 2019nCoV or nCoV or n-CoV or "CoV 2" or CoV2 or SARS-CoV-2 or SARS-CoV2 or SARSCoV-2 or SARSCoV2 or SARS2 or SARS-2 or SARS or MERS or respiratory syndrome* or pneumovirus* or pneumo-virus* or HRSV or HRSVs or RSV or RSVs or sync#tial virus* or sync#tial pneumovirus* or sync#tial pneumo-virus* or parainfluenza* or para-influenza* or RTI or RTIs or URTI or URTIs or LRTI or LRTIs or viral pneumonia* or pneumonia virus* or (respiratory adj2 infection*) or (respiratory adj2 virus*) or (respiratory adj2 illness*)) adj5 spread*).tw,kw. (25038)

458 ((disease* or infect* or viral* or virus*) adj5 expos*).tw,kw. (112436)

459 ((Influenza* or flu or flus or grippe or common cold* or colds or COVID or COVID-19 or COVID19 or coronavirus* or corona virus* or 2019-nCoV or 19nCoV or 2019nCoV or nCoV or n-CoV or "CoV 2" or CoV2 or SARS-CoV-2 or SARS-CoV2 or SARSCoV-2 or SARSCoV2 or SARS2 or SARS-2 or SARS or MERS or respiratory syndrome* or pneumovirus* or pneumo-virus* or HRSV or HRSVs or RSV or RSVs or sync#tial virus* or sync#tial pneumovirus* or sync#tial pneumo-virus* or parainfluenza* or para-influenza* or RTI or RTIs or URTI or URTIs or LRTI or LRTIs or viral pneumonia* or pneumonia virus* or (respiratory adj2 infection*) or (respiratory adj2 virus*) or (respiratory adj2 illness*)) adj5 expos*).tw,kw. (13276)

460 ((disease* or infect* or viral* or virus*) adj5 contact*).tw,kw. (32584)

461 ((Influenza* or flu or flus or grippe or common cold* or colds or COVID or COVID-19 or COVID19 or coronavirus* or corona virus* or 2019-nCoV or 19nCoV or 2019nCoV or nCoV or n-CoV or "CoV 2" or CoV2 or SARS-CoV-2 or SARS-CoV2 or SARSCoV-2 or SARSCoV2 or SARS2 or SARS-2 or SARS or MERS or respiratory syndrome* or pneumovirus* or pneumo-virus* or HRSV or HRSVs or RSV or RSVs or sync#tial virus* or sync#tial pneumovirus* or sync#tial pneumo-virus* or parainfluenza* or para-influenza* or RTI or RTIs or URTI or URTIs or LRTI or LRTIs or viral pneumonia* or pneumonia virus* or (respiratory adj2 infection*) or (respiratory adj2 virus*) or (respiratory adj2 illness*)) adj5 contact*).tw,kw. (5672)

462 occupational exposure/ (142940)

463 ((health care or healthcare or hospital* or occupation* or employee* or personnel* or procedur* or staff* or work*) adj5 (contact* or expos*)).tw,kw. (217390)

464 (air adj3 (microbiol* or micro-biol* or transmi*)).tw,kw. (1581)

465 (risk* adj5 (contact* or expos* or spread* or transmi*)).tw,kw. (219609)

466 or/452-465 [DISEASE TRANSMISSION/EXPOSURE] (1018464)

467 451 and 466 [AEROSOL PROCEDURES - HEALTHCARE WORKERS - RESPIRATORY VIRAL INFECTIONS - DISEASE TRANSMISSION Pt 1] (4779)

468 393 and 450 [AEROSOL PROCEDURES - HEALTHCARE WORKERS - RESPIRATORY VIRAL INFECTIONS - DISEASE TRANSMISSION Pt 2] (1122)

469 467 or 468 [AEROSOL PROCEDURES - HEALTHCARE WORKERS - RESPIRATORY VIRAL INFECTIONS - DISEASE TRANSMISSION - COMBINED] (5019)

470 exp animal/ or exp animal experimentation/ or exp animal model/ or exp animal experiment/ or nonhuman/ or exp vertebrate/ (54597866)

471 exp human/ or exp human experimentation/ or exp human experiment/ (42966401)

472 470 not 471 (11633200)

473 469 not 472 [ANIMAL-ONLY REMOVED] (4974)

474 conference abstract.pt. (4187998)

475 473 not 474 [CONFERENCE ABSTRACTS REMOVED] (4660)

476 475 use oemezd [EMBASE RECORDS] (2786)

477 exp Aerosols/ (94139)

478 (aerosoli#e* or aerosoli#ing).ti,ab,kw. (17867)

479 (aerosol* adj3 (contact* or dispers* or expos* or generat* or procedure* or produc* or spread* or transmi*)).ti,ab,kw. (20740)

480 ((AGP or AGPs or AGMP or AGMPs or AGDP or AGDPs) and aerosol*).ti,ab,kw. (306)

481 (aerosol* adj5 (clinic* or dent* or medic* or therap* or treatment*)).ti,ab,kw. (11521)

482 (bioaerosol* or bio-aerosol*).ti,ab,kw. (4764)

483 ("3-in-1" adj2 syringe?).ti,ab,kw. (29)

484 (water adj1 air? adj2 syringe?).ti,ab,kw. (227)

485 ((air rota or air rotar*) and dent*).ti,ab,kw. (1)

486 (((polish* or rotar* or finishing or handpiece* or hand piece*) adj3 (device* or equipment* or instrument*)) and dent*).ti,ab,kw. (2098)

487 ((ultrasonic* or sonic*) adj3 (scale* or scaling)).ti,ab,kw. (2010)

488 (air polish* or air abrasion* or air turbine handpiece? or air turbine hand piece?).ti,ab,kw. (2108)

489 (dent* adj3 (handpiece* or hand piece*) adj5 (highspeed* or high speed* or lowspeed* or low speed*)).ti,ab,kw. (159)

490 Autopsy/ (195259)

491 (autops* or postmortem exam* or post-mortem exam*).ti,ab,kw. (197930)

492 bag-valve*.ti,ab,kw. (1672)

493 (BVM adj10 (bag? or valve? or mask*)).ti,ab,kw. (391)

494 (ambu bag? or bag-mask? or (bag? adj1 resuscitat*) or (manual* adj1 resuscitat?) or self-inflating bag? or selfinflating bag? or "Revivator-Plus").ti,ab,kw. (2519)

495 exp Positive-Pressure Respiration/ (40077)

496 ((positive end-expiratory or (positive adj2 pressure)) adj (breathing or ventilat*)).ti,ab,kw. (18936)

497 positive airway pressure.ti,ab,kw. (36009)

498 (PEEP and (positive or expiratory or pressure)).ti,ab,kw. (14412)

499 (APRV or BiPAP or BPAP or CPCP or nCPAP or CPPB or CPPV or IPPB or IPPV).ti,ab,kw. (11314)

500 Noninvasive Ventilation/ (16076)

501 ((noninvasive* or non-invasive*) adj3 ventilat*).ti,ab,kw. (29974)

502 (NIV adj10 (noninvasive* or non-invasive* or ventilat*)).ti,ab,kw. (8745)

503 (noninvasive PPV or non-invasive PPV or NIPPV or NPPV).ti,ab,kw. (3797)

504 Oxygen/ and (atomi#er? or inhaler* or inhalator* or inhalation device? or nebuli* or vapo?ri*).ti,ab,kw. (1859)

505 (oxygen* adj5 (atomi#er? or inhaler* or inhalator* or inhalation device? or nebuli* or vapo?ri*)).ti,ab,kw. (1348)

506 ((air or gas$3) adj5 (atomi#er? or inhaler* or inhalator* or inhalation device? or nebuli* or vapo?ri*)).ti,ab,kw. (2872)

507 FLO2max*.ti,ab,kw. (0)

508 Bronchial Provocation Tests/ (13462)

509 ((bronchial* or endobronchial or endo-bronchial) adj3 (challenge? or provocation)).ti,ab,kw. (7991)

510 (BP test* adj10 bronch*).ti,ab,kw. (5)

511 ((inhalation adj3 (challenge? or provocation)) and test*).ti,ab,kw. (2275)

512 "Nebulizers and Vaporizers"/ (20187)

513 exp Metered Dose Inhalers/ (9341)

514 (nebulize* or nebulise* or (inhal* adj3 aerosol*)).ti,ab,kw. (37483)

515 Aerogen*.ti,ab,kw. (8618)

516 Bronchoscopy/ (79805)

517 (bronchoscop* or broncho-scop*).ti,ab,kw. (80152)

518 (bronch* adj3 endoscop*).ti,ab,kw. (2123)

519 (laryngotracheobronchoscop* or laryngo-tracheobronchoscop* or laryngo-tracheo-bronchoscop* or laryngotracheo-bronchoscop* or tracheobronchoscop* or tracheo-bronchoscop*).ti,ab,kw. (592)

520 exp Cardiopulmonary Resuscitation/ (138942)

521 ((cardiopulmonary or cardio-pulmonary or mouth-to-mouth) adj3 resuscitat*).ti,ab,kw. (44701)

522 (CPR and (BVM or respirat* or resuscitat* or intubat*)).ti,ab,kw. (25131)

523 (chest adj3 compress*).ti,ab,kw. (13249)

524 (cough* adj2 assist*).ti,ab,kw. (903)

525 (CoughAssist* or Pegaso* or Cofflator* or Cof-flator* or cough machine*).ti,ab,kw. (211)

526 (cough* adj2 augment*).ti,ab,kw. (232)

527 Cough/rh [Rehabilitation] (25)

528 Insufflation/ (17767)

529 ("in-exsufflator" or "in-exsufflators" or "in-exsufflation" or "in-exsufflations").ti,ab,kw. (296)

530 (insufflat* adj1 exsufflat*).ti,ab,kw. (511)

531 "MI-E".ti,ab,kw. (311)

532 (breathstack* or breath-stack*).ti,ab,kw. (181)

533 (airstack* or air-stack*).ti,ab,kw. (149)

534 (direct* adj2 cough*).ti,ab,kw. (176)

535 ((glossopharyn* or glosso-pharyn*) adj2 (breath* or respirat*)).ti,ab,kw. (168)

536 (cough* adj2 flow* adj5 (improv* or increas* or enhanc* or expan* or exten*)).ti,ab,kw. (188)

537 (respiratory muscle* adj2 (aid* or support*)).ti,ab,kw. (107)

538 (recruit* adj2 ("lung volume" or alveolar)).ti,ab,kw. (2420)

539 ((lung or alveolar) adj1 recruit* adj2 man?euv*).ti,ab,kw. (1094)

540 ((LVRM or LVR) adj10 (lung or volume or recruit* or man?euv*)).ti,ab,kw. (458)

541 Frozen Sections/is, mt [Instrumentation, Methods] (475)

542 (frozen adj2 (section* or specimen* or tissue*) adj3 (biops* or cut or cuts or cutting* or knife? or knives or shave or shaved or shaves or shaving* or microtome*)).ti,ab,kw. (2835)

543 (cryostat* or cryomicrotome* or cryo-microtome* or freezing microtome*).ti,ab,kw. (12350)

544 Suction/ (25121)

545 suction*.ti,ab,kw. (50552)

546 (aspirat* adj3 mechanical*).ti,ab,kw. (1017)

547 exp High-Frequency Ventilation/ (7579)

548 ((highfrequen* or high-frequen*) adj3 (ventilat* or oscillat*)).ti,ab,kw. (15533)

549 ((HFV or HFJV or HFOV) adj10 ventilat*).ti,ab,kw. (3489)

550 (HFV adj3 oscillat*).ti,ab,kw. (37)

551 Oxygen Inhalation Therapy/ (50738)

552 (("high flow" or highflow) adj5 (oxygen* or O2 or nasal* or can?ul*)).ti,ab,kw. (9651)

553 ((HHHFO or HHFO) and (heat* or humid* or "high flow" or highflow or oxygen* or O2)).ti,ab,kw. (2)

554 (AIRVO* or Optiflow* or Vapotherm*).ti,ab,kw. (656)

555 ((oxygen* or O2) adj10 flush flow*).ti,ab,kw. (2)

556 Venturi*.ti,ab,kw. (3536)

557 ((oxygen* or O2) adj5 (nonhumid* or non-humid* or dry)).ti,ab,kw. (1147)

558 ((non-rebreath* mask* or nonrebreath* mask* or NRBM or NRBMs) and (nasal can?ula* or nose tube? or nasal tube?)).ti,ab,kw. (144)

559 exp Intubation/ (163859)

560 Airway Extubation/ (24006)

561 (intubat* or extubat* or detubat*).ti,ab,kw. (209121)

562 Respiration, Artificial/ (191676)

563 (artificial* adj3 respirat*).ti,ab,kw. (5275)

564 (manual* adj3 ventilat*).ti,ab,kw. (2306)

565 (manual* adj3 resuscitat*).ti,ab,kw. (745)

566 ((selfinflat* or self-inflat* or flowinflat* or flow-inflat*) adj3 resuscitat*).ti,ab,kw. (228)

567 (T-piece adj3 resuscitat*).ti,ab,kw. (301)

568 exp Ventilators, Mechanical/ (14562)

569 (mechanical* adj3 ventilat*).ti,ab,kw. (167336)

570 (mechanical* adj3 resuscitat*).ti,ab,kw. (1368)

571 (support* adj3 (ventilat* or resuscitat*)).ti,ab,kw. (34130)

572 (nasopharyngoscop* or naso-pharyngoscop* or fiberoptic* nasendoscop* or fiber-optic* nasendoscop* or fibreoptic* nasendoscop* or fibre-optic* nasendoscop* or nasolaryngoscop* or naso-laryngoscop* or nasopharyngolaryngoscop* or naso-pharyngolaryngoscop* or (flexible adj2 laryngoscop*)).ti,ab,kw. (3560)

573 Tracheostomy/ (36680)

574 tracheostom*.ti,ab,kw. (41090)

575 Laryngectomy/ (21821)

576 (laryngectom* or laryn-gectom*).ti,ab,kw. (18376)

577 Sputum/ (48810)

578 (sputum* or expectorat* or spit*).ti,ab,kw. (288490)

579 ((supraglottic or supra-glottic) adj airway*).ti,ab,kw. (3471)

580 laryngeal airway*.ti,ab,kw. (676)

581 Airway Management/ and Laryngeal Masks/ (1464)

582 (LMA and (supraglottic or supra-glottic)).ti,ab,kw. (1103)

583 (I-GEL or I-GELR or I-GELTM).ti,ab,kw. (2292)

584 (King LT or King LTR or King LTTM).ti,ab,kw. (89)

585 (PAxpress* or SLIPA or SLIPAR or SLIPATM).ti,ab,kw. (176)

586 ((transsphenoid* or trans-sphenoid*) adj3 (surger* or surgical*)).ti,ab,kw. (9806)

587 Sphenoid Bone/su [surgery] (1804)

588 Sphenoid Sinus/su [surgery] (1619)

589 Aerosol Propellants/ (2361)

590 propellant*.ti,ab,kw. (4164)

591 (compress* adj1 (gas or gases or gasses) adj5 (administ* or deliver* or method* or treat* or therap*)).ti,ab,kw. (78)

592 ((analg?esi* or an?esthe*) and (cryo* or freez* or frozen*) and (agent* or spray*)).ti,ab,kw. (803)

593 (cryoan?esthesi* or cryo-an?esthesi* or cryoanalgesi* or cryo-analgesi*).ti,ab,kw. (616)

594 (cold adj (analg?esi* or an?esthe*)).ti,ab,kw. (105)

595 ((oral* or spray*) adj5 lidocaine*).ti,ab,kw. (2257)

596 Perfusion Imaging/ (31105)

597 (VQ scan* or "V/Q scan*" or (ventilation adj1 perfusion adj3 scan*) or (lung adj1 perfusion adj3 scan*) or (pulmonary adj1 perfusion adj3 scan*)).ti,ab,kw. (6401)

598 (VQ scintigraph* or "V/Q scintigraph*" or (ventilation adj1 perfusion adj3 scintigraph*) or (lung adj1 perfusion adj3 scintigraph*) or (pulmonary adj1 perfusion adj3 scintigraph*)).ti,ab,kw. (2590)

599 (VQ scintiscan* or "V/Q scintiscan*" or (ventilation adj1 perfusion adj3 scintiscan*) or (lung adj1 perfusion adj3 scintiscan*) or (pulmonary adj1 perfusion adj3 scintiscan*)).ti,ab,kw. (38)

600 (VQ scintigram* or "V/Q scintigram*" or (ventilation adj1 perfusion adj3 scintigram*) or (lung adj1 perfusion adj3 scintigram*) or (pulmonary adj1 perfusion adj3 scintigram*)).ti,ab,kw. (251)

601 (VQ scintiphoto* or "V/Q scintiphoto*" or (ventilation adj1 perfusion adj3 scintiphoto*) or (lung adj1 perfusion adj3 scintiphoto*) or (pulmonary adj1 perfusion adj3 scintiphoto*)).ti,ab,kw. (18)

602 (VQ scinti-photo* or "V/Q scinti-photo*" or (ventilation adj1 perfusion adj3 scinti-photo*) or (lung adj1 perfusion adj3 scinti-photo*) or (pulmonary adj1 perfusion adj3 scinti-photo*)).ti,ab,kw. (0)

603 droplet?.ti,ab,kw. (110627)

604 ((secrete* or secreti*) adj3 (breath* or cough* or mouth* or mucus* or nasal* or nose? or oral* or nasopharyn* or naso-pharyn* or oropharyn* or oro-pharyn* or pharyn* or respirat* or saliva*)).ti,ab,kw. (38208)

605 or/477-604 [AEROSOL PROCEDURES] (1942255)

606 Cross Infection/tm [transmission] (5377)

607 (cross infection* adj5 (assistant? or employee* or personnel or professional? or staff or technician* or worker?)).ti,ab,kw. (209)

608 ((healthcare associated infection* or health care associated infection*) adj5 (assistant? or employee* or personnel or professional? or staff or technician* or worker?)).ti,ab,kw. (209)

609 ((healthcare associated transmi* or health care associated transmi*) adj5 (assistant? or employee* or personnel or professional? or staff or technician* or worker?)).ti,ab,kw. (6)

610 (hospital infection* adj5 (assistant? or employee* or personnel or professional? or staff or technician* or worker?)).ti,ab,kw. (265)

611 (nosocomial infection* adj5 (assistant? or employee* or personnel or professional? or staff or technician* or worker?)).ti,ab,kw. (381)

612 (nosocomial adj5 transmi* adj5 (assistant? or employee* or personnel or professional? or staff or technician* or worker?)).ti,ab,kw. (255)

613 ((disease* or infect* or viral* or virus*) adj5 risk* adj5 (assistant? or employee* or personnel or professional? or staff or technician* or worker?)).ti,ab,kw. (7084)

614 Infectious Disease Transmission, Patient-to-Professional/ (109802)

615 (((disease* or infect* or viral* or virus*) adj3 transmi*) and (patient? adj5 (assistant? or employee* or personnel or professional? or staff or technician* or worker?))).ti,ab,kw. (2420)

616 (((disease* or infect* or viral* or virus*) adj3 spread*) and (patient? adj5 (assistant? or employee* or personnel or professional? or staff or technician* or worker?))).ti,ab,kw. (1137)

617 (((disease* or infect* or viral* or virus*) adj3 expos*) and (patient? adj5 (assistant? or employee* or personnel or professional? or staff or technician* or worker?))).ti,ab,kw. (685)

618 (((disease* or infect* or viral* or virus*) adj3 contact*) and (patient? adj5 (assistant? or employee* or personnel or professional? or staff or technician* or worker?))).ti,ab,kw. (407)

619 (((disease* or infect* or viral* or virus*) adj3 transmi*) and (patient? adj5 (allergist* or an?esthet?ist* or an?esthesia assistant* or an?esthesiologist* or audiologist* or cardiologist* or dental technician* or dentist* or dermatologist* or doctor* or emergency medical technician* or endocrinologist* or endodontist* or exodontist* or gastroenterologist* or gastro-enterologist* or general practitioner* or geriatrician* or gyn?ecologist* or hospitalist* or nephrologist* or neurologist* or nurse* or obstetrician* or oncologist* or ophthalmologist* or orthodontist* or osteopath or osteopaths or otolaryngologist* or paramedic* or pathologist* or p?ediatrician* or periodontist* or physiatrist* or physician* or physiotherapist* or physio-therapist* or prosthodontist* or pulmonologist* or radiologist* or respiratory technician* or rheumatologist* or surgeon* or therapist* or urologist*))).ti,ab,kw. (1874)

620 (((disease* or infect* or viral* or virus*) adj3 spread*) and (patient? adj5 (allergist* or an?esthet?ist* or an?esthesia assistant* or an?esthesiologist* or audiologist* or cardiologist* or dental technician* or dentist* or dermatologist* or doctor* or emergency medical technician* or endocrinologist* or endodontist* or exodontist* or gastroenterologist* or gastro-enterologist* or general practitioner* or geriatrician* or gyn?ecologist* or hospitalist* or nephrologist* or neurologist* or nurse* or obstetrician* or oncologist* or ophthalmologist* or orthodontist* or osteopath or osteopaths or otolaryngologist* or paramedic* or pathologist* or p?ediatrician* or periodontist* or physiatrist* or physician* or physiotherapist* or physio-therapist* or prosthodontist* or pulmonologist* or radiologist* or respiratory technician* or rheumatologist* or surgeon* or therapist* or urologist*))).ti,ab,kw. (626)

621 (((disease* or infect* or viral* or virus*) adj3 expos*) and (patient? adj5 (allergist* or an?esthet?ist* or an?esthesia assistant* or an?esthesiologist* or audiologist* or cardiologist* or dental technician* or dentist* or dermatologist* or doctor* or emergency medical technician* or endocrinologist* or endodontist* or exodontist* or gastroenterologist* or gastro-enterologist* or general practitioner* or geriatrician* or gyn?ecologist* or hospitalist* or nephrologist* or neurologist* or nurse* or obstetrician* or oncologist* or ophthalmologist* or orthodontist* or osteopath or osteopaths or otolaryngologist* or paramedic* or pathologist* or p?ediatrician* or periodontist* or physiatrist* or physician* or physiotherapist* or physio-therapist* or prosthodontist* or pulmonologist* or radiologist* or respiratory technician* or rheumatologist* or surgeon* or therapist* or urologist*))).ti,ab,kw. (586)

622 (((disease* or infect* or viral* or virus*) adj3 contact*) and (patient? adj5 (allergist* or an?esthet?ist* or an?esthesia assistant* or an?esthesiologist* or audiologist* or cardiologist* or dental technician* or dentist* or dermatologist* or doctor* or emergency medical technician* or endocrinologist* or endodontist* or exodontist* or gastroenterologist* or gastro-enterologist* or general practitioner* or geriatrician* or gyn?ecologist* or hospitalist* or nephrologist* or neurologist* or nurse* or obstetrician* or oncologist* or ophthalmologist* or orthodontist* or osteopath or osteopaths or otolaryngologist* or paramedic* or pathologist* or p?ediatrician* or periodontist* or physiatrist* or physician* or physiotherapist* or physio-therapist* or prosthodontist* or pulmonologist* or radiologist* or respiratory technician* or rheumatologist* or surgeon* or therapist* or urologist*))).ti,ab,kw. (293)

623 ((disease* or infect* or viral* or virus*) adj5 risk* adj5 (allergist* or an?esthet?ist* or an?esthesia assistant* or an?esthesiologist* or audiologist* or cardiologist* or dental technician* or dentist* or dermatologist* or doctor* or emergency medical technician* or endocrinologist* or endodontist* or exodontist* or gastroenterologist* or gastro-enterologist* or general practitioner* or geriatrician* or gyn?ecologist* or hospitalist* or nephrologist* or neurologist* or nurse* or obstetrician* or oncologist* or ophthalmologist* or orthodontist* or osteopath or osteopaths or otolaryngologist* or paramedic* or pathologist* or p?ediatrician* or periodontist* or physiatrist* or physician* or physiotherapist* or physio-therapist* or prosthodontist* or pulmonologist* or radiologist* or respiratory technician* or rheumatologist* or surgeon* or therapist* or urologist*)).ti,ab,kw. (3742)

624 or/606-623 [DISEASE RISK/TRANSMISSION - PATIENT TO PROFESSIONAL] (131236)

625 605 and 624 [AEROSOL PROCEDURES - DISEASE RISK/TRANSMISSION - PATIENT TO PROFESSIONAL] (6703)

626 exp Health Personnel/ (2261940)

627 ((allied health adj3 personnel) or (allied health adj3 employee*) or (allied health adj3 staff) or (allied health adj3 worker*) or (health care adj3 assistant*) or (healthcare adj3 assistant*) or (health care adj3 employee*) or (healthcare adj3 employee*) or (health care adj3 personnel) or (healthcare adj3 personnel) or (health care adj3 staff) or (healthcare adj3 staff) or (health care adj3 worker*) or (healthcare adj3 worker*) or (health adj3 personnel) or (health adj3 employee*) or (health adj3 staff) or (health adj3 worker*) or (health adj3 assistant*) or (health care adj3 professional*) or (healthcare adj3 professional*) or (health care adj3 provider*) or (healthcare adj3 provider*) or (hospital adj3 assistant*) or (hospital adj3 employee*) or (hospital adj3 personnel) or (hospital adj3 staff) or (hospital adj3 worker*) or (medical adj3 assistant*) or (medical adj3 employee*) or (medical adj3 personnel) or (medical adj3 staff) or (medical adj3 worker*) or (nursing adj3 assistant*) or (nursing adj3 employee*) or (nursing adj3 personnel*) or (nursing adj3 staff) or (nursing adj3 worker*) or (critical care adj3 assistant*) or (critical care adj3 employee*) or (critical care adj3 personnel) or (critical care adj3 staff) or (critical care adj3 worker*) or (intensive care adj3 assistant*) or (intensive care adj3 employee*) or (intensive care adj3 personnel) or (intensive care adj3 staff) or (intensive care adj3 worker*) or (ICU adj3 assistant*) or (ICU adj3 employee*) or (ICU adj3 personnel) or (ICU adj3 staff) or (ICU adj3 worker*) or (respiratory adj3 assistant*) or (respiratory adj3 employee*) or (respiratory adj3 personnel) or (respiratory adj3 staff) or (respiratory adj3 worker*) or (emergency adj3 assistant*) or (emergency adj3 employee*) or (emergency adj3 personnel) or (emergency adj3 staff) or (emergency adj3 worker*)).ti,ab,kw. (614611)

628 (allergist* or an?esthet?ist* or an?esthesia assistant* or an?esthesiologist* or audiologist* or cardiologist* or dental assistant* or dental personnel or dental staff or dental technician* or dentist* or dermatologist* or doctor* or emergency medical personnel or emergency medical technician* or endocrinologist* or endodontist* or exodontist* or gastroenterologist* or gastro-enterologist* or general practitioner* or geriatrician* or gyn?ecologist* or hospitalist* or nephrologist* or neurologist* or nurse* or obstetrician* or occupational therapist* or oncologist* or ophthalmologist* or orthodontist* or osteopath or osteopaths or otolaryngologist* or paramedic* or pathologist* or p?ediatrician* or periodontist* or physiatrist* or physician* or physiotherapist* or physio-therapist* or prosthodontist* or pulmonologist* or radiologist* or respiratory technician* or rheumatologist* or surgeon* or therapist* or urologist*).ti,ab,kw. (3434806)

629 or/626-628 [HEALTHCARE WORKERS] (4840536)

630 605 and 629 [AEROSOL PROCEDURES - HEALTHCARE WORKERS] (192550)

631 Influenza, Human/ (70922)

632 exp Influenza A virus/ (62663)

633 Influenza B virus/ (6536)

634 Influenzavirus C/ (355)

635 (influenza* or flu or grippe).ti,ab,kw. (300233)

636 (H1N1 or PH1N1 or H3N2 or AH1N1 or "A(H1N1)" or "A/H1N1" or AH3N2 or "A(H3N2)" or "A/H3N2" or H5N1).ti,ab,kw. (62489)

637 Common Cold/ (13700)

638 (common cold or colds).ti,ab,kw. (14225)

639 coryza.ti. (293)

640 COVID-19/ (104862)

641 SARS-CoV-2/ (101517)

642 Coronavirus/ (11706)

643 Betacoronavirus/ (41005)

644 Coronavirus Infections/ (57090)

645 (COVID-19 or COVID19).ti,ab,kw. (312554)

646 ((coronavirus* or corona virus*) and (hubei or wuhan or beijing or shanghai)).ti,ab,kw. (10772)

647 (wuhan adj5 virus*).ti,ab,kw. (586)

648 (2019-nCoV or 19nCoV or 2019nCoV).ti,ab,kw. (3690)

649 (nCoV or n-CoV or "CoV 2" or CoV2).ti,ab,kw. (112137)

650 (SARS-CoV-2 or SARS-CoV2 or SARSCoV-2 or SARSCoV2 or SARS2 or SARS-2 or severe acute respiratory syndrome coronavirus 2).ti,ab,kw. (121796)

651 (2019-novel CoV or Sars-coronavirus2 or Sars-coronavirus-2 or SARS-like coronavirus* or ((novel or new or nouveau) adj2 (CoV or nCoV or covid or coronavirus* or corona virus or Pandemi*2)) or (coronavirus* and pneumonia)).ti,ab,kw. (40934)

652 (novel coronavirus* or novel corona virus* or novel CoV).ti,ab,kw. (20359)

653 ((coronavirus* or corona virus*) adj2 "2019").ti,ab,kw. (69477)

654 ((coronavirus* or corona virus*) adj2 "19").ti,ab,kw. (10600)

655 ("coronavirus 2" or "corona virus 2").ti,ab,kw. (35849)

656 (OC43 or NL63 or 229E or HKU1 or HCoV* or Sars-coronavirus*).ti,ab,kw. (8129)

657 (COVID-19 or severe acute respiratory syndrome coronavirus 2).ti,ab,kw. (315988)

658 (coronavirus* or corona virus*).ti. (47697)

659 COVID.ti. (246259)

660 ("B.1.1.7" or "B.1.351" or "B.1.617" or "B.1.427" or "B.1.429").ti,ab,kw. (1261)

661 ("P.1" and (Brazil* or variant?)).ti,ab,kw. (3665)

662 (((alpha or beta or delta or eta or gamma or iota or kappa or lambda) adj3 variant?) and (coronavirus* or corona virus* or covid*)).ti,ab,kw. (248)

663 SARS Virus/ (10760)

664 Severe Acute Respiratory Syndrome/ (16143)

665 ((severe acute or acute severe) adj2 respiratory syndrome*).ti,ab,kw. (48920)

666 SARS.ti,ab,kw. (131733)

667 Middle East Respiratory Syndrome Coronavirus/ (5547)

668 (middle east adj2 respiratory syndrome*).ti,ab,kw. (6591)

669 MERS.ti,ab,kw. (14176)

670 Pneumovirus Infections/ (281)

671 (pneumovirus* or pneumo-virus*).ti,ab,kw. (872)

672 Respiratory Syncytial Virus Infections/ (10910)

673 Respiratory Syncytial Viruses/ or Respiratory Syncytial Virus, Human/ (15129)

674 (respiratory adj1 sync#tial adj2 (virus* or pneumovir* or pneumo-vir*)).ti,ab,kw. (33816)

675 (HRSV or HRSVs or RSV or RSVs or sync#tial virus* or sync#tial pneumovirus* or sync#tial pneumo-virus*).ti,ab,kw. (44779)

676 (parainfluenza* or para-influenza*).ti,ab,kw. (14831)

677 Respiratory Tract Infections/ (67150)

678 (respiratory adj3 (infectio* or illness* or virus*)).ti,ab,kw. (200311)

679 ((ARI or ARIs) adj5 (acute or respirator* or infect* or illness*)).ti,ab,kw. (4509)

680 (RTI or RTIs or URTI or URTIs or LRTI or LRTIs).ti,ab,kw. (14965)

681 Pneumonia, Viral/ (63969)

682 (pneumonia* adj3 (viral* or virus*)).ti,ab,kw. (10435)

683 or/631-682 [RESPIRATORY VIRAL INFECTIONS] (934072)

684 630 and 683 [AEROSOL PROCEDURES - HEALTHCARE WORKERS - RESPIRATORY VIRAL INFECTIONS] (12737)

685 Cross Infection/tm [transmission] (5377)

686 Disease Transmission, Infectious/ (115345)

687 Infectious Disease Transmission, Patient-to-Professional/ (109802)

688 Influenza, Human/tm [transmission] (3426)

689 Common Cold/tm [transmission] (106)

690 COVID-19/tm [transmission] (3680)

691 Coronavirus Infections/tm [transmission] (4704)

692 Severe Acute Respiratory Syndrome/tm [transmission] (934)

693 Pneumovirus Infections/tm [transmission] (6)

694 Respiratory Syncytial Virus Infections/tm [transmission] (151)

695 Respiratory Tract Infections/tm [transmission] (587)

696 Pneumonia, Viral/tm [transmission] (4203)

697 ((disease* or infect* or viral* or virus*) adj5 transmi*).ti,ab,kw. (258663)

698 ((Influenza* or flu or flus or grippe or common cold* or colds or COVID or COVID-19 or COVID19 or coronavirus* or corona virus* or 2019-nCoV or 19nCoV or 2019nCoV or nCoV or n-CoV or "CoV 2" or CoV2 or SARS-CoV-2 or SARS-CoV2 or SARSCoV-2 or SARSCoV2 or SARS2 or SARS-2 or SARS or MERS or respiratory syndrome* or pneumovirus* or pneumo-virus* or HRSV or HRSVs or RSV or RSVs or sync#tial virus* or sync#tial pneumovirus* or sync#tial pneumo-virus* or parainfluenza* or para-influenza* or RTI or RTIs or URTI or URTIs or LRTI or LRTIs or viral pneumonia* or pneumonia virus* or (respiratory adj2 infection*) or (respiratory adj2 virus*) or (respiratory adj2 illness*)) adj5 transmi*).ti,ab,kw. (24266)

699 ((disease* or infect* or viral* or virus*) adj5 spread*).ti,ab,kw. (96838)

700 ((Influenza* or flu or flus or grippe or common cold* or colds or COVID or COVID-19 or COVID19 or coronavirus* or corona virus* or 2019-nCoV or 19nCoV or 2019nCoV or nCoV or n-CoV or "CoV 2" or CoV2 or SARS-CoV-2 or SARS-CoV2 or SARSCoV-2 or SARSCoV2 or SARS2 or SARS-2 or SARS or MERS or respiratory syndrome* or pneumovirus* or pneumo-virus* or HRSV or HRSVs or RSV or RSVs or sync#tial virus* or sync#tial pneumovirus* or sync#tial pneumo-virus* or parainfluenza* or para-influenza* or RTI or RTIs or URTI or URTIs or LRTI or LRTIs or viral pneumonia* or pneumonia virus* or (respiratory adj2 infection*) or (respiratory adj2 virus*) or (respiratory adj2 illness*)) adj5 spread*).ti,ab,kw. (25009)

701 ((disease* or infect* or viral* or virus*) adj5 expos*).ti,ab,kw. (112186)

702 ((Influenza* or flu or flus or grippe or common cold* or colds or COVID or COVID-19 or COVID19 or coronavirus* or corona virus* or 2019-nCoV or 19nCoV or 2019nCoV or nCoV or n-CoV or "CoV 2" or CoV2 or SARS-CoV-2 or SARS-CoV2 or SARSCoV-2 or SARSCoV2 or SARS2 or SARS-2 or SARS or MERS or respiratory syndrome* or pneumovirus* or pneumo-virus* or HRSV or HRSVs or RSV or RSVs or sync#tial virus* or sync#tial pneumovirus* or sync#tial pneumo-virus* or parainfluenza* or para-influenza* or RTI or RTIs or URTI or URTIs or LRTI or LRTIs or viral pneumonia* or pneumonia virus* or (respiratory adj2 infection*) or (respiratory adj2 virus*) or (respiratory adj2 illness*)) adj5 expos*).ti,ab,kw. (13247)

703 ((disease* or infect* or viral* or virus*) adj5 contact*).ti,ab,kw. (32396)

704 ((Influenza* or flu or flus or grippe or common cold* or colds or COVID or COVID-19 or COVID19 or coronavirus* or corona virus* or 2019-nCoV or 19nCoV or 2019nCoV or nCoV or n-CoV or "CoV 2" or CoV2 or SARS-CoV-2 or SARS-CoV2 or SARSCoV-2 or SARSCoV2 or SARS2 or SARS-2 or SARS or MERS or respiratory syndrome* or pneumovirus* or pneumo-virus* or HRSV or HRSVs or RSV or RSVs or sync#tial virus* or sync#tial pneumovirus* or sync#tial pneumo-virus* or parainfluenza* or para-influenza* or RTI or RTIs or URTI or URTIs or LRTI or LRTIs or viral pneumonia* or pneumonia virus* or (respiratory adj2 infection*) or (respiratory adj2 virus*) or (respiratory adj2 illness*)) adj5 contact*).ti,ab,kw. (5649)

705 Occupational Exposure/ (142940)

706 ((health care or healthcare or hospital* or occupation* or employee* or personnel* or procedur* or staff* or work*) adj5 (contact* or expos*)).ti,ab,kw. (216698)

707 Air Microbiology/ (262736)

708 (air adj3 (microbiol* or micro-biol* or transmi*)).ti,ab,kw. (1576)

709 (risk* adj5 (contact* or expos* or spread* or transmi*)).ti,ab,kw. (218792)

710 or/685-709 [DISEASE TRANSMISSION/EXPOSURE] (1242322)

711 684 and 710 [AEROSOL PROCEDURES - HEALTHCARE WORKERS - RESPIRATORY VIRAL INFECTIONS - DISEASE TRANSMISSION Pt 1] (4486)

712 625 and 683 [AEROSOL PROCEDURES - HEALTHCARE WORKERS - RESPIRATORY VIRAL INFECTIONS - DISEASE TRANSMISSION Pt 2] (2809)

713 711 or 712 [AEROSOL PROCEDURES - HEALTHCARE WORKERS - RESPIRATORY VIRAL INFECTIONS - DISEASE TRANSMISSION - COMBINED] (5654)

714 (conference abstract or journal conference abstract).pt. (4368620)

715 713 not 714 [CONFERENCE ABSTRACTS REMOVED] (5295)

716 715 use cctr [CENTRAL RECORDS] (124)

717 (aerosoli#e* or aerosoli#ing).ti,ab,kw. (17867)

718 (aerosol* adj3 (contact* or dispers* or expos* or generat* or procedure* or produc* or spread* or transmi*)).ti,ab,kw. (20740)

719 ((AGP or AGPs or AGMP or AGMPs or AGDP or AGDPs) and aerosol*).ti,ab,kw. (306)

720 (aerosol* adj5 (clinic* or dent* or medic* or therap* or treatment*)).ti,ab,kw. (11521)

721 (bioaerosol* or bio-aerosol*).ti,ab,kw. (4764)

722 ("3-in-1" adj2 syringe?).ti,ab,kw. (29)

723 (water adj1 air? adj2 syringe?).ti,ab,kw. (227)

724 ((air rota or air rotar*) and dent*).ti,ab,kw. (1)

725 (((polish* or rotar* or finishing or handpiece* or hand piece*) adj3 (device* or equipment* or instrument*)) and dent*).ti,ab,kw. (2098)

726 ((ultrasonic* or sonic*) adj3 (scale* or scaling)).ti,ab,kw. (2010)

727 (air polish* or air abrasion* or air turbine handpiece? or air turbine hand piece?).ti,ab,kw. (2108)

728 (dent* adj3 (handpiece* or hand piece*) adj5 (highspeed* or high speed* or lowspeed* or low speed*)).ti,ab,kw. (159)

729 (autops* or postmortem exam* or post-mortem exam*).ti,ab,kw. (197930)

730 bag-valve*.ti,ab,kw. (1672)

731 (BVM adj10 (bag? or valve? or mask*)).ti,ab,kw. (391)

732 (ambu bag? or bag-mask? or (bag? adj1 resuscitat*) or (manual* adj1 resuscitat?) or self-inflating bag? or selfinflating bag? or "Revivator-Plus").ti,ab,kw. (2519)

733 ((positive end-expiratory or (positive adj2 pressure)) adj (breathing or ventilat*)).ti,ab,kw. (18936)

734 positive airway pressure.ti,ab,kw. (36009)

735 (PEEP and (positive or expiratory or pressure)).ti,ab,kw. (14412)

736 (APRV or BiPAP or BPAP or CPCP or nCPAP or CPPB or CPPV or IPPB or IPPV).ti,ab,kw. (11314)

737 ((noninvasive* or non-invasive*) adj3 ventilat*).ti,ab,kw. (29974)

738 (NIV adj10 (noninvasive* or non-invasive* or ventilat*)).ti,ab,kw. (8745)

739 (noninvasive PPV or non-invasive PPV or NIPPV or NPPV).ti,ab,kw. (3797)

740 (oxygen* adj5 (atomi#er? or inhaler* or inhalator* or inhalation device? or nebuli* or vapo?ri*)).ti,ab,kw. (1348)

741 ((air or gas$3) adj5 (atomi#er? or inhaler* or inhalator* or inhalation device? or nebuli* or vapo?ri*)).ti,ab,kw. (2872)

742 FLO2max*.ti,ab,kw. (0)

743 ((bronchial* or endobronchial or endo-bronchial) adj3 (challenge? or provocation)).ti,ab,kw. (7991)

744 (BP test* adj10 bronch*).ti,ab,kw. (5)

745 ((inhalation adj3 (challenge? or provocation)) and test*).ti,ab,kw. (2275)

746 (nebulize* or nebulise* or (inhal* adj3 aerosol*)).ti,ab,kw. (37483)

747 Aerogen*.ti,ab,kw. (8618)

748 (bronchoscop* or broncho-scop*).ti,ab,kw. (80152)

749 (bronch* adj3 endoscop*).ti,ab,kw. (2123)

750 (laryngotracheobronchoscop* or laryngo-tracheobronchoscop* or laryngo-tracheo-bronchoscop* or laryngotracheo-bronchoscop* or tracheobronchoscop* or tracheo-bronchoscop*).ti,ab,kw. (592)

751 ((cardiopulmonary or cardio-pulmonary or mouth-to-mouth) adj3 resuscitat*).ti,ab,kw. (44701)

752 (CPR and (BVM or respirat* or resuscitat* or intubat*)).ti,ab,kw. (25131)

753 (chest adj3 compress*).ti,ab,kw. (13249)

754 (cough* adj2 assist*).ti,ab,kw. (903)

755 (CoughAssist* or Pegaso* or Cofflator* or Cof-flator* or cough machine*).ti,ab,kw. (211)

756 (cough* adj2 augment*).ti,ab,kw. (232)

757 ("in-exsufflator" or "in-exsufflators" or "in-exsufflation" or "in-exsufflations").ti,ab,kw. (296)

758 (insufflat* adj1 exsufflat*).ti,ab,kw. (511)

759 "MI-E".ti,ab,kw. (311)

760 (breathstack* or breath-stack*).ti,ab,kw. (181)

761 (airstack* or air-stack*).ti,ab,kw. (149)

762 (direct* adj2 cough*).ti,ab,kw. (176)

763 ((glossopharyn* or glosso-pharyn*) adj2 (breath* or respirat*)).ti,ab,kw. (168)

764 (cough* adj2 flow* adj5 (improv* or increas* or enhanc* or expan* or exten*)).ti,ab,kw. (188)

765 (respiratory muscle* adj2 (aid* or support*)).ti,ab,kw. (107)

766 (recruit* adj2 ("lung volume" or alveolar)).ti,ab,kw. (2420)

767 ((lung or alveolar) adj1 recruit* adj2 man?euv*).ti,ab,kw. (1094)

768 ((LVRM or LVR) adj10 (lung or volume or recruit* or man?euv*)).ti,ab,kw. (458)

769 (frozen adj2 (section* or specimen* or tissue*) adj3 (biops* or cut or cuts or cutting* or knife? or knives or shave or shaved or shaves or shaving* or microtome*)).ti,ab,kw. (2835)

770 (cryostat* or cryomicrotome* or cryo-microtome* or freezing microtome*).ti,ab,kw. (12350)

771 suction*.ti,ab,kw. (50552)

772 (aspirat* adj3 mechanical*).ti,ab,kw. (1017)

773 ((highfrequen* or high-frequen*) adj3 (ventilat* or oscillat*)).ti,ab,kw. (15533)

774 ((HFV or HFJV or HFOV) adj10 ventilat*).ti,ab,kw. (3489)

775 (HFV adj3 oscillat*).ti,ab,kw. (37)

776 (("high flow" or highflow) adj5 (oxygen* or O2 or nasal* or can?ul*)).ti,ab,kw. (9651)

777 ((HHHFO or HHFO) and (heat* or humid* or "high flow" or highflow or oxygen* or O2)).ti,ab,kw. (2)

778 (AIRVO* or Optiflow* or Vapotherm*).ti,ab,kw. (656)

779 ((oxygen* or O2) adj10 flush flow*).ti,ab,kw. (2)

780 Venturi*.ti,ab,kw. (3536)

781 ((oxygen* or O2) adj5 (nonhumid* or non-humid* or dry)).ti,ab,kw. (1147)

782 ((non-rebreath* mask* or nonrebreath* mask* or NRBM or NRBMs) and (nasal can?ula* or nose tube? or nasal tube?)).ti,ab,kw. (144)

783 (intubat* or extubat* or detubat*).ti,ab,kw. (209121)

784 (artificial* adj3 respirat*).ti,ab,kw. (5275)

785 (manual* adj3 ventilat*).ti,ab,kw. (2306)

786 (manual* adj3 resuscitat*).ti,ab,kw. (745)

787 ((selfinflat* or self-inflat* or flowinflat* or flow-inflat*) adj3 resuscitat*).ti,ab,kw. (228)

788 (T-piece adj3 resuscitat*).ti,ab,kw. (301)

789 (mechanical* adj3 ventilat*).ti,ab,kw. (167336)

790 (mechanical* adj3 resuscitat*).ti,ab,kw. (1368)

791 (support* adj3 (ventilat* or resuscitat*)).ti,ab,kw. (34130)

792 (nasopharyngoscop* or naso-pharyngoscop* or fiberoptic* nasendoscop* or fiber-optic* nasendoscop* or fibreoptic* nasendoscop* or fibre-optic* nasendoscop* or nasolaryngoscop* or naso-laryngoscop* or nasopharyngolaryngoscop* or naso-pharyngolaryngoscop* or (flexible adj2 laryngoscop*)).ti,ab,kw. (3560)

793 tracheostom*.ti,ab,kw. (41090)

794 (laryngectom* or laryn-gectom*).ti,ab,kw. (18376)

795 (sputum* or expectorat* or spit*).ti,ab,kw. (288490)

796 ((supraglottic or supra-glottic) adj airway*).ti,ab,kw. (3471)

797 laryngeal airway*.ti,ab,kw. (676)

798 (LMA and (supraglottic or supra-glottic)).ti,ab,kw. (1103)

799 (I-GEL or I-GELR or I-GELTM).ti,ab,kw. (2292)

800 (King LT or King LTR or King LTTM).ti,ab,kw. (89)

801 (PAxpress* or SLIPA or SLIPAR or SLIPATM).ti,ab,kw. (176)

802 ((transsphenoid* or trans-sphenoid*) adj3 (surger* or surgical*)).ti,ab,kw. (9806)

803 propellant*.ti,ab,kw. (4164)

804 (compress* adj1 (gas or gases or gasses) adj5 (administ* or deliver* or method* or treat* or therap*)).ti,ab,kw. (78)

805 ((analg?esi* or an?esthe*) and (cryo* or freez* or frozen*) and (agent* or spray*)).ti,ab,kw. (803)

806 (cryoan?esthesi* or cryo-an?esthesi* or cryoanalgesi* or cryo-analgesi*).ti,ab,kw. (616)

807 (cold adj (analg?esi* or an?esthe*)).ti,ab,kw. (105)

808 ((oral* or spray*) adj5 lidocaine*).ti,ab,kw. (2257)

809 (VQ scan* or "V/Q scan*" or (ventilation adj1 perfusion adj3 scan*) or (lung adj1 perfusion adj3 scan*) or (pulmonary adj1 perfusion adj3 scan*)).ti,ab,kw. (6401)

810 (VQ scintigraph* or "V/Q scintigraph*" or (ventilation adj1 perfusion adj3 scintigraph*) or (lung adj1 perfusion adj3 scintigraph*) or (pulmonary adj1 perfusion adj3 scintigraph*)).ti,ab,kw. (2590)

811 (VQ scintiscan* or "V/Q scintiscan*" or (ventilation adj1 perfusion adj3 scintiscan*) or (lung adj1 perfusion adj3 scintiscan*) or (pulmonary adj1 perfusion adj3 scintiscan*)).ti,ab,kw. (38)

812 (VQ scintigram* or "V/Q scintigram*" or (ventilation adj1 perfusion adj3 scintigram*) or (lung adj1 perfusion adj3 scintigram*) or (pulmonary adj1 perfusion adj3 scintigram*)).ti,ab,kw. (251)

813 (VQ scintiphoto* or "V/Q scintiphoto*" or (ventilation adj1 perfusion adj3 scintiphoto*) or (lung adj1 perfusion adj3 scintiphoto*) or (pulmonary adj1 perfusion adj3 scintiphoto*)).ti,ab,kw. (18)

814 (VQ scinti-photo* or "V/Q scinti-photo*" or (ventilation adj1 perfusion adj3 scinti-photo*) or (lung adj1 perfusion adj3 scinti-photo*) or (pulmonary adj1 perfusion adj3 scinti-photo*)).ti,ab,kw. (0)

815 droplet?.ti,ab,kw. (110627)

816 ((secrete* or secreti*) adj3 (breath* or cough* or mouth* or mucus* or nasal* or nose? or oral* or nasopharyn* or naso-pharyn* or oropharyn* or oro-pharyn* or pharyn* or respirat* or saliva*)).ti,ab,kw. (38208)

817 or/717-816 [AEROSOL PROCEDURES] (1382538)

818 (cross infection* adj5 (assistant? or employee* or personnel or professional? or staff or technician* or worker?)).ti,ab,kw. (209)

819 ((healthcare associated infection* or health care associated infection*) adj5 (assistant? or employee* or personnel or professional? or staff or technician* or worker?)).ti,ab,kw. (209)

820 ((healthcare associated transmi* or health care associated transmi*) adj5 (assistant? or employee* or personnel or professional? or staff or technician* or worker?)).ti,ab,kw. (6)

821 (hospital infection* adj5 (assistant? or employee* or personnel or professional? or staff or technician* or worker?)).ti,ab,kw. (265)

822 (nosocomial infection* adj5 (assistant? or employee* or personnel or professional? or staff or technician* or worker?)).ti,ab,kw. (381)

823 (nosocomial adj5 transmi* adj5 (assistant? or employee* or personnel or professional? or staff or technician* or worker?)).ti,ab,kw. (255)

824 ((disease* or infect* or viral* or virus*) adj5 risk* adj5 (assistant? or employee* or personnel or professional? or staff or technician* or worker?)).ti,ab,kw. (7084)

825 (((disease* or infect* or viral* or virus*) adj3 transmi*) and (patient? adj5 (assistant? or employee* or personnel or professional? or staff or technician* or worker?))).ti,ab,kw. (2420)

826 (((disease* or infect* or viral* or virus*) adj3 spread*) and (patient? adj5 (assistant? or employee* or personnel or professional? or staff or technician* or worker?))).ti,ab,kw. (1137)

827 (((disease* or infect* or viral* or virus*) adj3 expos*) and (patient? adj5 (assistant? or employee* or personnel or professional? or staff or technician* or worker?))).ti,ab,kw. (685)

828 (((disease* or infect* or viral* or virus*) adj3 contact*) and (patient? adj5 (assistant? or employee* or personnel or professional? or staff or technician* or worker?))).ti,ab,kw. (407)

829 (((disease* or infect* or viral* or virus*) adj3 transmi*) and (patient? adj5 (allergist* or an?esthet?ist* or an?esthesia assistant* or an?esthesiologist* or audiologist* or cardiologist* or dental technician* or dentist* or dermatologist* or doctor* or emergency medical technician* or endocrinologist* or endodontist* or exodontist* or gastroenterologist* or gastro-enterologist* or general practitioner* or geriatrician* or gyn?ecologist* or hospitalist* or nephrologist* or neurologist* or nurse* or obstetrician* or oncologist* or ophthalmologist* or orthodontist* or osteopath or osteopaths or otolaryngologist* or paramedic* or pathologist* or p?ediatrician* or periodontist* or physiatrist* or physician* or physiotherapist* or physio-therapist* or prosthodontist* or pulmonologist* or radiologist* or respiratory technician* or rheumatologist* or surgeon* or therapist* or urologist*))).ti,ab,kw. (1874)

830 (((disease* or infect* or viral* or virus*) adj3 spread*) and (patient? adj5 (allergist* or an?esthet?ist* or an?esthesia assistant* or an?esthesiologist* or audiologist* or cardiologist* or dental technician* or dentist* or dermatologist* or doctor* or emergency medical technician* or endocrinologist* or endodontist* or exodontist* or gastroenterologist* or gastro-enterologist* or general practitioner* or geriatrician* or gyn?ecologist* or hospitalist* or nephrologist* or neurologist* or nurse* or obstetrician* or oncologist* or ophthalmologist* or orthodontist* or osteopath or osteopaths or otolaryngologist* or paramedic* or pathologist* or p?ediatrician* or periodontist* or physiatrist* or physician* or physiotherapist* or physio-therapist* or prosthodontist* or pulmonologist* or radiologist* or respiratory technician* or rheumatologist* or surgeon* or therapist* or urologist*))).ti,ab,kw. (626)

831 (((disease* or infect* or viral* or virus*) adj3 expos*) and (patient? adj5 (allergist* or an?esthet?ist* or an?esthesia assistant* or an?esthesiologist* or audiologist* or cardiologist* or dental technician* or dentist* or dermatologist* or doctor* or emergency medical technician* or endocrinologist* or endodontist* or exodontist* or gastroenterologist* or gastro-enterologist* or general practitioner* or geriatrician* or gyn?ecologist* or hospitalist* or nephrologist* or neurologist* or nurse* or obstetrician* or oncologist* or ophthalmologist* or orthodontist* or osteopath or osteopaths or otolaryngologist* or paramedic* or pathologist* or p?ediatrician* or periodontist* or physiatrist* or physician* or physiotherapist* or physio-therapist* or prosthodontist* or pulmonologist* or radiologist* or respiratory technician* or rheumatologist* or surgeon* or therapist* or urologist*))).ti,ab,kw. (586)

832 (((disease* or infect* or viral* or virus*) adj3 contact*) and (patient? adj5 (allergist* or an?esthet?ist* or an?esthesia assistant* or an?esthesiologist* or audiologist* or cardiologist* or dental technician* or dentist* or dermatologist* or doctor* or emergency medical technician* or endocrinologist* or endodontist* or exodontist* or gastroenterologist* or gastro-enterologist* or general practitioner* or geriatrician* or gyn?ecologist* or hospitalist* or nephrologist* or neurologist* or nurse* or obstetrician* or oncologist* or ophthalmologist* or orthodontist* or osteopath or osteopaths or otolaryngologist* or paramedic* or pathologist* or p?ediatrician* or periodontist* or physiatrist* or physician* or physiotherapist* or physio-therapist* or prosthodontist* or pulmonologist* or radiologist* or respiratory technician* or rheumatologist* or surgeon* or therapist* or urologist*))).ti,ab,kw. (293)

833 ((disease* or infect* or viral* or virus*) adj5 risk* adj5 (allergist* or an?esthet?ist* or an?esthesia assistant* or an?esthesiologist* or audiologist* or cardiologist* or dental technician* or dentist* or dermatologist* or doctor* or emergency medical technician* or endocrinologist* or endodontist* or exodontist* or gastroenterologist* or gastro-enterologist* or general practitioner* or geriatrician* or gyn?ecologist* or hospitalist* or nephrologist* or neurologist* or nurse* or obstetrician* or oncologist* or ophthalmologist* or orthodontist* or osteopath or osteopaths or otolaryngologist* or paramedic* or pathologist* or p?ediatrician* or periodontist* or physiatrist* or physician* or physiotherapist* or physio-therapist* or prosthodontist* or pulmonologist* or radiologist* or respiratory technician* or rheumatologist* or surgeon* or therapist* or urologist*)).ti,ab,kw. (3742)

834 or/818-833 [DISEASE RISK/TRANSMISSION - PATIENT TO PROFESSIONAL] (18321)

835 817 and 834 [AEROSOL PROCEDURES - DISEASE RISK/TRANSMISSION - PATIENT TO PROFESSIONAL] (1562)

836 ((allied health adj3 personnel) or (allied health adj3 employee*) or (allied health adj3 staff) or (allied health adj3 worker*) or (health care adj3 assistant*) or (healthcare adj3 assistant*) or (health care adj3 employee*) or (healthcare adj3 employee*) or (health care adj3 personnel) or (healthcare adj3 personnel) or (health care adj3 staff) or (healthcare adj3 staff) or (health care adj3 worker*) or (healthcare adj3 worker*) or (health adj3 personnel) or (health adj3 employee*) or (health adj3 staff) or (health adj3 worker*) or (health adj3 assistant*) or (health care adj3 professional*) or (healthcare adj3 professional*) or (health care adj3 provider*) or (healthcare adj3 provider*) or (hospital adj3 assistant*) or (hospital adj3 employee*) or (hospital adj3 personnel) or (hospital adj3 staff) or (hospital adj3 worker*) or (medical adj3 assistant*) or (medical adj3 employee*) or (medical adj3 personnel) or (medical adj3 staff) or (medical adj3 worker*) or (nursing adj3 assistant*) or (nursing adj3 employee*) or (nursing adj3 personnel*) or (nursing adj3 staff) or (nursing adj3 worker*) or (critical care adj3 assistant*) or (critical care adj3 employee*) or (critical care adj3 personnel) or (critical care adj3 staff) or (critical care adj3 worker*) or (intensive care adj3 assistant*) or (intensive care adj3 employee*) or (intensive care adj3 personnel) or (intensive care adj3 staff) or (intensive care adj3 worker*) or (ICU adj3 assistant*) or (ICU adj3 employee*) or (ICU adj3 personnel) or (ICU adj3 staff) or (ICU adj3 worker*) or (respiratory adj3 assistant*) or (respiratory adj3 employee*) or (respiratory adj3 personnel) or (respiratory adj3 staff) or (respiratory adj3 worker*) or (emergency adj3 assistant*) or (emergency adj3 employee*) or (emergency adj3 personnel) or (emergency adj3 staff) or (emergency adj3 worker*)).ti,ab,kw. (614611)

837 (allergist* or an?esthet?ist* or an?esthesia assistant* or an?esthesiologist* or audiologist* or cardiologist* or dental assistant* or dental personnel or dental staff or dental technician* or dentist* or dermatologist* or doctor* or emergency medical personnel or emergency medical technician* or endocrinologist* or endodontist* or exodontist* or gastroenterologist* or gastro-enterologist* or general practitioner* or geriatrician* or gyn?ecologist* or hospitalist* or nephrologist* or neurologist* or nurse* or obstetrician* or occupational therapist* or oncologist* or ophthalmologist* or orthodontist* or osteopath or osteopaths or otolaryngologist* or paramedic* or pathologist* or p?ediatrician* or periodontist* or physiatrist* or physician* or physiotherapist* or physio-therapist* or prosthodontist* or pulmonologist* or radiologist* or respiratory technician* or rheumatologist* or surgeon* or therapist* or urologist*).ti,ab,kw. (3434806)

838 or/836-837 [HEALTHCARE WORKERS] (3848603)

839 817 and 838 [AEROSOL PROCEDURES - HEALTHCARE WORKERS] (128235)

840 (influenza* or flu or grippe).ti,ab,kw. (300233)

841 (H1N1 or PH1N1 or H3N2 or AH1N1 or "A(H1N1)" or "A/H1N1" or AH3N2 or "A(H3N2)" or "A/H3N2" or H5N1).ti,ab,kw. (62489)

842 (common cold or colds).ti,ab,kw. (14225)

843 coryza.ti. (293)

844 (COVID-19 or COVID19).ti,ab,kw. (312554)

845 ((coronavirus* or corona virus*) and (hubei or wuhan or beijing or shanghai)).ti,ab,kw. (10772)

846 (wuhan adj5 virus*).ti,ab,kw. (586)

847 (2019-nCoV or 19nCoV or 2019nCoV).ti,ab,kw. (3690)

848 (nCoV or n-CoV or "CoV 2" or CoV2).ti,ab,kw. (112137)

849 (SARS-CoV-2 or SARS-CoV2 or SARSCoV-2 or SARSCoV2 or SARS2 or SARS-2 or severe acute respiratory syndrome coronavirus 2).ti,ab,kw. (121796)

850 (2019-novel CoV or Sars-coronavirus2 or Sars-coronavirus-2 or SARS-like coronavirus* or ((novel or new or nouveau) adj2 (CoV or nCoV or covid or coronavirus* or corona virus or Pandemi*2)) or (coronavirus* and pneumonia)).ti,ab,kw. (40934)

851 (novel coronavirus* or novel corona virus* or novel CoV).ti,ab,kw. (20359)

852 ((coronavirus* or corona virus*) adj2 "2019").ti,ab,kw. (69477)

853 ((coronavirus* or corona virus*) adj2 "19").ti,ab,kw. (10600)

854 ("coronavirus 2" or "corona virus 2").ti,ab,kw. (35849)

855 (OC43 or NL63 or 229E or HKU1 or HCoV* or Sars-coronavirus*).ti,ab,kw. (8129)

856 (COVID-19 or severe acute respiratory syndrome coronavirus 2).ti,ab,kw. (315988)

857 (coronavirus* or corona virus*).ti. (47697)

858 COVID.ti. (246259)

859 ("B.1.1.7" or "B.1.351" or "B.1.617" or "B.1.427" or "B.1.429").ti,ab,kw. (1261)

860 ("P.1" and (Brazil* or variant?)).ti,ab,kw. (3665)

861 (((alpha or beta or delta or eta or gamma or iota or kappa or lambda) adj3 variant?) and (coronavirus* or corona virus* or covid*)).ti,ab,kw. (248)

862 ((severe acute or acute severe) adj2 respiratory syndrome*).ti,ab,kw. (48920)

863 SARS.ti,ab,kw. (131733)

864 (middle east adj2 respiratory syndrome*).ti,ab,kw. (6591)

865 MERS.ti,ab,kw. (14176)

866 (pneumovirus* or pneumo-virus*).ti,ab,kw. (872)

867 (respiratory adj1 sync#tial adj2 (virus* or pneumovir* or pneumo-vir*)).ti,ab,kw. (33816)

868 (HRSV or HRSVs or RSV or RSVs or sync#tial virus* or sync#tial pneumovirus* or sync#tial pneumo-virus*).ti,ab,kw. (44779)

869 (parainfluenza* or para-influenza*).ti,ab,kw. (14831)

870 (respiratory adj3 (infectio* or illness* or virus*)).ti,ab,kw. (200311)

871 ((ARI or ARIs) adj5 (acute or respirator* or infect* or illness*)).ti,ab,kw. (4509)

872 (RTI or RTIs or URTI or URTIs or LRTI or LRTIs).ti,ab,kw. (14965)

873 (pneumonia* adj3 (viral* or virus*)).ti,ab,kw. (10435)

874 or/840-873 [RESPIRATORY VIRAL INFECTIONS] (871166)

875 839 and 874 [AEROSOL PROCEDURES - HEALTHCARE WORKERS - RESPIRATORY VIRAL INFECTIONS] (8515)

876 ((disease* or infect* or viral* or virus*) adj5 transmi*).ti,ab,kw. (258663)

877 ((Influenza* or flu or flus or grippe or common cold* or colds or COVID or COVID-19 or COVID19 or coronavirus* or corona virus* or 2019-nCoV or 19nCoV or 2019nCoV or nCoV or n-CoV or "CoV 2" or CoV2 or SARS-CoV-2 or SARS-CoV2 or SARSCoV-2 or SARSCoV2 or SARS2 or SARS-2 or SARS or MERS or respiratory syndrome* or pneumovirus* or pneumo-virus* or HRSV or HRSVs or RSV or RSVs or sync#tial virus* or sync#tial pneumovirus* or sync#tial pneumo-virus* or parainfluenza* or para-influenza* or RTI or RTIs or URTI or URTIs or LRTI or LRTIs or viral pneumonia* or pneumonia virus* or (respiratory adj2 infection*) or (respiratory adj2 virus*) or (respiratory adj2 illness*)) adj5 transmi*).ti,ab,kw. (24266)

878 ((disease* or infect* or viral* or virus*) adj5 spread*).ti,ab,kw. (96838)

879 ((Influenza* or flu or flus or grippe or common cold* or colds or COVID or COVID-19 or COVID19 or coronavirus* or corona virus* or 2019-nCoV or 19nCoV or 2019nCoV or nCoV or n-CoV or "CoV 2" or CoV2 or SARS-CoV-2 or SARS-CoV2 or SARSCoV-2 or SARSCoV2 or SARS2 or SARS-2 or SARS or MERS or respiratory syndrome* or pneumovirus* or pneumo-virus* or HRSV or HRSVs or RSV or RSVs or sync#tial virus* or sync#tial pneumovirus* or sync#tial pneumo-virus* or parainfluenza* or para-influenza* or RTI or RTIs or URTI or URTIs or LRTI or LRTIs or viral pneumonia* or pneumonia virus* or (respiratory adj2 infection*) or (respiratory adj2 virus*) or (respiratory adj2 illness*)) adj5 spread*).ti,ab,kw. (25009)

880 ((disease* or infect* or viral* or virus*) adj5 expos*).ti,ab,kw. (112186)

881 ((Influenza* or flu or flus or grippe or common cold* or colds or COVID or COVID-19 or COVID19 or coronavirus* or corona virus* or 2019-nCoV or 19nCoV or 2019nCoV or nCoV or n-CoV or "CoV 2" or CoV2 or SARS-CoV-2 or SARS-CoV2 or SARSCoV-2 or SARSCoV2 or SARS2 or SARS-2 or SARS or MERS or respiratory syndrome* or pneumovirus* or pneumo-virus* or HRSV or HRSVs or RSV or RSVs or sync#tial virus* or sync#tial pneumovirus* or sync#tial pneumo-virus* or parainfluenza* or para-influenza* or RTI or RTIs or URTI or URTIs or LRTI or LRTIs or viral pneumonia* or pneumonia virus* or (respiratory adj2 infection*) or (respiratory adj2 virus*) or (respiratory adj2 illness*)) adj5 expos*).ti,ab,kw. (13247)

882 ((disease* or infect* or viral* or virus*) adj5 contact*).ti,ab,kw. (32396)

883 ((Influenza* or flu or flus or grippe or common cold* or colds or COVID or COVID-19 or COVID19 or coronavirus* or corona virus* or 2019-nCoV or 19nCoV or 2019nCoV or nCoV or n-CoV or "CoV 2" or CoV2 or SARS-CoV-2 or SARS-CoV2 or SARSCoV-2 or SARSCoV2 or SARS2 or SARS-2 or SARS or MERS or respiratory syndrome* or pneumovirus* or pneumo-virus* or HRSV or HRSVs or RSV or RSVs or sync#tial virus* or sync#tial pneumovirus* or sync#tial pneumo-virus* or parainfluenza* or para-influenza* or RTI or RTIs or URTI or URTIs or LRTI or LRTIs or viral pneumonia* or pneumonia virus* or (respiratory adj2 infection*) or (respiratory adj2 virus*) or (respiratory adj2 illness*)) adj5 contact*).ti,ab,kw. (5649)

884 ((health care or healthcare or hospital* or occupation* or employee* or personnel* or procedur* or staff* or work*) adj5 (contact* or expos*)).ti,ab,kw. (216698)

885 (air adj3 (microbiol* or micro-biol* or transmi*)).ti,ab,kw. (1576)

886 (risk* adj5 (contact* or expos* or spread* or transmi*)).ti,ab,kw. (218792)

887 or/876-886 [DISEASE TRANSMISSION/EXPOSURE] (838923)

888 875 and 887 [AEROSOL PROCEDURES - HEALTHCARE WORKERS - RESPIRATORY VIRAL INFECTIONS - DISEASE TRANSMISSION Pt 1] (3136)

889 835 and 874 [AEROSOL PROCEDURES - HEALTHCARE WORKERS - RESPIRATORY VIRAL INFECTIONS - DISEASE TRANSMISSION Pt 2] (957)

890 888 or 889 [AEROSOL PROCEDURES - HEALTHCARE WORKERS - RESPIRATORY VIRAL INFECTIONS - DISEASE TRANSMISSION - COMBINED] (3366)

891 890 use coch [CDSR RECORDS] (6)

892 240 or 476 or 716 or 891 [ALL DATABASES] (5001)

893 remove duplicates from 892 (3594) [TOTAL UNIQUE RECORDS]

894 893 use ppez [MEDLINE UNIQUE RECORDS] (2070)

895 893 use oemezd [EMBASE UNIQUE RECORDS] (1415)

896 893 use cctr [CENTRAL UNIQUE RECORDS] (103)

897 893 use coch [CDSR UNIQUE RECORDS] (6)

***************************

*2021 Sep 9*

CINAHL

| # | Query | Limiters/Expanders | Last Run Via | Results |
| --- | --- | --- | --- | --- |
| S229 | S227 OR S228 | Search modes - Find all my search terms | Interface - EBSCOhost Research Databases  Search Screen - Advanced Search  Database - CINAHL Plus with Full Text | 681 |
| S228 | S147 AND S200 | Search modes - Find all my search terms | Interface - EBSCOhost Research Databases  Search Screen - Advanced Search  Database - CINAHL Plus with Full Text | 304 |
| S227 | S201 AND S226 | Search modes - Find all my search terms | Interface - EBSCOhost Research Databases  Search Screen - Advanced Search  Database - CINAHL Plus with Full Text | 595 |
| S226 | S202 OR S203 OR S204 OR S205 OR S206 OR S207 OR S208 OR S209 OR S210 OR S211 OR S212 OR S213 OR S214 OR S215 OR S216 OR S217 OR S218 OR S219 OR S220 OR S221 OR S222 OR S223 OR S224 OR S225 | Search modes - Find all my search terms | Interface - EBSCOhost Research Databases  Search Screen - Advanced Search  Database - CINAHL Plus with Full Text | 102,125 |
| S225 | TI ( risk* N5 (contact* or expos* or spread* or transmi*) ) OR AB ( risk* N5 (contact* or expos* or spread* or transmi*) ) | Search modes - Find all my search terms | Interface - EBSCOhost Research Databases  Search Screen - Advanced Search  Database - CINAHL Plus with Full Text | 27,598 |
| S224 | TI ( air N3 (microbiol* or micro-biol* or transmi*) ) OR AB ( air N3 (microbiol* or micro-biol* or transmi*) ) | Search modes - Find all my search terms | Interface - EBSCOhost Research Databases  Search Screen - Advanced Search  Database - CINAHL Plus with Full Text | 113 |
| S223 | (MH "Air Microbiology") | Search modes - Find all my search terms | Interface - EBSCOhost Research Databases  Search Screen - Advanced Search  Database - CINAHL Plus with Full Text | 584 |
| S222 | TI ( ("health care" or healthcare or hospital* or occupation* or employee* or personnel* or procedur* or staff* or work*) N5 (contact* or expos*) ) OR AB ( ("health care" or healthcare or hospital* or occupation* or employee* or personnel* or procedur* or staff* or work*) N5 (contact* or expos*) ) | Search modes - Find all my search terms | Interface - EBSCOhost Research Databases  Search Screen - Advanced Search  Database - CINAHL Plus with Full Text | 24,477 |
| S221 | (MH "Occupational Exposure") | Search modes - Find all my search terms | Interface - EBSCOhost Research Databases  Search Screen - Advanced Search  Database - CINAHL Plus with Full Text | 19,955 |
| S220 | TI ( (influenza* or flu or flus or grippe or (common W0 cold*) or colds or COVID or "COVID-19" or COVID19 or coronavirus* or (corona W0 virus*) or "2019-nCoV" or 19nCoV or 2019nCoV or nCoV or "n-CoV" or "CoV 2" or CoV2 or "SARS-CoV-2" or "SARS-CoV2" or "SARSCoV-2" or SARSCoV2 or SARS2 or "SARS-2" or SARS or MERS or (respiratory W0 syndrome*) or pneumovirus* or (pneumo W0 virus*) or HRSV or HRSVs or RSV or RSVs or (sync?tial W0 virus*) or (sync?tial W0 pneumovirus*) or (sync?tial W0 pneumo W0 virus*) or parainfluenza* or (para W0 influenza*) or RTI or RTIs or URTI or URTIs or LRTI or LRTIs or (viral W0 pneumonia*) or (pneumonia W0 virus*) or (respiratory N2 infection*) or (respiratory N2 virus*) or (respiratory N2 illness*)) N5 contact* ) OR AB ( (influenza* or flu or flus or grippe or (common W0 cold*) or colds or COVID or "COVID-19" or COVID19 or coronavirus* or (corona W0 virus*) or "2019-nCoV" or 19nCoV or 2019nCoV or nCoV or "n-CoV" or "CoV 2" or CoV2 or "SARS-CoV-2" or "SARS-CoV2" or "SARSCoV-2" or SARSCoV2 or SARS2 or "SARS-2" or SARS or MERS or (respiratory W0 syndrome*) or pneumovirus* or (pneumo W0 virus*) or HRSV or HRSVs or RSV or RSVs or (sync?tial W0 virus*) or (sync?tial W0 pneumovirus*) or (sync?tial W0 pneumo W0 virus*) or parainfluenza* or (para W0 influenza*) or RTI or RTIs or URTI or URTIs or LRTI or LRTIs or (viral W0 pneumonia*) or (pneumonia W0 virus*) or (respiratory N2 infection*) or (respiratory N2 virus*) or (respiratory N2 illness*)) N5 contact* ) | Search modes - Find all my search terms | Interface - EBSCOhost Research Databases  Search Screen - Advanced Search  Database - CINAHL Plus with Full Text | 939 |
| S219 | TI ( (disease* or infect* or viral* or virus*) N5 contact* ) OR AB ( (disease* or infect* or viral* or virus*) N5 contact* ) | Search modes - Find all my search terms | Interface - EBSCOhost Research Databases  Search Screen - Advanced Search  Database - CINAHL Plus with Full Text | 2,747 |
| S218 | TI ( (influenza* or flu or flus or grippe or (common W0 cold*) or colds or COVID or "COVID-19" or COVID19 or coronavirus* or (corona W0 virus*) or "2019-nCoV" or 19nCoV or 2019nCoV or nCoV or "n-CoV" or "CoV 2" or CoV2 or "SARS-CoV-2" or "SARS-CoV2" or "SARSCoV-2" or SARSCoV2 or SARS2 or "SARS-2" or SARS or MERS or (respiratory W0 syndrome*) or pneumovirus* or (pneumo W0 virus*) or HRSV or HRSVs or RSV or RSVs or (sync?tial W0 virus*) or (sync?tial W0 pneumovirus*) or (sync?tial W0 pneumo W0 virus*) or parainfluenza* or (para W0 influenza*) or RTI or RTIs or URTI or URTIs or LRTI or LRTIs or (viral W0 pneumonia*) or (pneumonia W0 virus*) or (respiratory N2 infection*) or (respiratory N2 virus*) or (respiratory N2 illness*)) N5 expos* ) OR AB ( (influenza* or flu or flus or grippe or (common W0 cold*) or colds or COVID or "COVID-19" or COVID19 or coronavirus* or (corona W0 virus*) or "2019-nCoV" or 19nCoV or 2019nCoV or nCoV or "n-CoV" or "CoV 2" or CoV2 or "SARS-CoV-2" or "SARS-CoV2" or "SARSCoV-2" or SARSCoV2 or SARS2 or "SARS-2" or SARS or MERS or (respiratory W0 syndrome*) or pneumovirus* or (pneumo W0 virus*) or HRSV or HRSVs or RSV or RSVs or (sync?tial W0 virus*) or (sync?tial W0 pneumovirus*) or (sync?tial W0 pneumo W0 virus*) or parainfluenza* or (para W0 influenza*) or RTI or RTIs or URTI or URTIs or LRTI or LRTIs or (viral W0 pneumonia*) or (pneumonia W0 virus*) or (respiratory N2 infection*) or (respiratory N2 virus*) or (respiratory N2 illness*)) N5 expos* ) | Search modes - Find all my search terms | Interface - EBSCOhost Research Databases  Search Screen - Advanced Search  Database - CINAHL Plus with Full Text | 2,416 |
| S217 | TI ( (disease* or infect* or viral* or virus*) N5 expos* ) OR AB ( (disease* or infect* or viral* or virus*) N5 expos* ) | Search modes - Find all my search terms | Interface - EBSCOhost Research Databases  Search Screen - Advanced Search  Database - CINAHL Plus with Full Text | 9,496 |
| S216 | TI ( (influenza* or flu or flus or grippe or (common W0 cold*) or colds or COVID or "COVID-19" or COVID19 or coronavirus* or (corona W0 virus*) or "2019-nCoV" or 19nCoV or 2019nCoV or nCoV or "n-CoV" or "CoV 2" or CoV2 or "SARS-CoV-2" or "SARS-CoV2" or "SARSCoV-2" or SARSCoV2 or SARS2 or "SARS-2" or SARS or MERS or (respiratory W0 syndrome*) or pneumovirus* or (pneumo W0 virus*) or HRSV or HRSVs or RSV or RSVs or (sync?tial W0 virus*) or (sync?tial W0 pneumovirus*) or (sync?tial W0 pneumo W0 virus*) or parainfluenza* or (para W0 influenza*) or RTI or RTIs or URTI or URTIs or LRTI or LRTIs or (viral W0 pneumonia*) or (pneumonia W0 virus*) or (respiratory N2 infection*) or (respiratory N2 virus*) or (respiratory N2 illness*)) N5 spread* ) OR AB ( (influenza* or flu or flus or grippe or (common W0 cold*) or colds or COVID or "COVID-19" or COVID19 or coronavirus* or (corona W0 virus*) or "2019-nCoV" or 19nCoV or 2019nCoV or nCoV or "n-CoV" or "CoV 2" or CoV2 or "SARS-CoV-2" or "SARS-CoV2" or "SARSCoV-2" or SARSCoV2 or SARS2 or "SARS-2" or SARS or MERS or (respiratory W0 syndrome*) or pneumovirus* or (pneumo W0 virus*) or HRSV or HRSVs or RSV or RSVs or (sync?tial W0 virus*) or (sync?tial W0 pneumovirus*) or (sync?tial W0 pneumo W0 virus*) or parainfluenza* or (para W0 influenza*) or RTI or RTIs or URTI or URTIs or LRTI or LRTIs or (viral W0 pneumonia*) or (pneumonia W0 virus*) or (respiratory N2 infection*) or (respiratory N2 virus*) or (respiratory N2 illness*)) N5 spread* ) | Search modes - Find all my search terms | Interface - EBSCOhost Research Databases  Search Screen - Advanced Search  Database - CINAHL Plus with Full Text | 3,069 |
| S215 | TI ( (disease* or infect* or viral* or virus*) N5 spread* ) OR AB ( (disease* or infect* or viral* or virus*) N5 spread* ) | Search modes - Find all my search terms | Interface - EBSCOhost Research Databases  Search Screen - Advanced Search  Database - CINAHL Plus with Full Text | 6,841 |
| S214 | TI ( (influenza* or flu or flus or grippe or (common W0 cold*) or colds or COVID or "COVID-19" or COVID19 or coronavirus* or (corona W0 virus*) or "2019-nCoV" or 19nCoV or 2019nCoV or nCoV or "n-CoV" or "CoV 2" or CoV2 or "SARS-CoV-2" or "SARS-CoV2" or "SARSCoV-2" or SARSCoV2 or SARS2 or "SARS-2" or SARS or MERS or (respiratory W0 syndrome*) or pneumovirus* or (pneumo W0 virus*) or HRSV or HRSVs or RSV or RSVs or (sync?tial W0 virus*) or (sync?tial W0 pneumovirus*) or (sync?tial W0 pneumo W0 virus*) or parainfluenza* or (para W0 influenza*) or RTI or RTIs or URTI or URTIs or LRTI or LRTIs or (viral W0 pneumonia*) or (pneumonia W0 virus*) or (respiratory N2 infection*) or (respiratory N2 virus*) or (respiratory N2 illness*)) N5 transmi* ) OR AB ( (influenza* or flu or flus or grippe or (common W0 cold*) or colds or COVID or "COVID-19" or COVID19 or coronavirus* or (corona W0 virus*) or "2019-nCoV" or 19nCoV or 2019nCoV or nCoV or "n-CoV" or "CoV 2" or CoV2 or "SARS-CoV-2" or "SARS-CoV2" or "SARSCoV-2" or SARSCoV2 or SARS2 or "SARS-2" or SARS or MERS or (respiratory W0 syndrome*) or pneumovirus* or (pneumo W0 virus*) or HRSV or HRSVs or RSV or RSVs or (sync?tial W0 virus*) or (sync?tial W0 pneumovirus*) or (sync?tial W0 pneumo W0 virus*) or parainfluenza* or (para W0 influenza*) or RTI or RTIs or URTI or URTIs or LRTI or LRTIs or (viral W0 pneumonia*) or (pneumonia W0 virus*) or (respiratory N2 infection*) or (respiratory N2 virus*) or (respiratory N2 illness*)) N5 transmi* ) | Search modes - Find all my search terms | Interface - EBSCOhost Research Databases  Search Screen - Advanced Search  Database - CINAHL Plus with Full Text | 3,067 |
| S213 | TI ( (disease* or infect* or viral* or virus*) N5 transmi* ) OR AB ( (disease* or infect* or viral* or virus*) N5 transmi* ) | Search modes - Find all my search terms | Interface - EBSCOhost Research Databases  Search Screen - Advanced Search  Database - CINAHL Plus with Full Text | 26,595 |
| S212 | (MH "Pneumonia, Viral/TM") | Search modes - Find all my search terms | Interface - EBSCOhost Research Databases  Search Screen - Advanced Search  Database - CINAHL Plus with Full Text | 926 |
| S211 | (MH "Respiratory Tract Infections/TM") | Search modes - Find all my search terms | Interface - EBSCOhost Research Databases  Search Screen - Advanced Search  Database - CINAHL Plus with Full Text | 189 |
| S210 | (MH "Respiratory Syncytial Virus Infections/TM") | Search modes - Find all my search terms | Interface - EBSCOhost Research Databases  Search Screen - Advanced Search  Database - CINAHL Plus with Full Text | 55 |
| S209 | (MH "Severe Acute Respiratory Syndrome/TM") | Search modes - Find all my search terms | Interface - EBSCOhost Research Databases  Search Screen - Advanced Search  Database - CINAHL Plus with Full Text | 301 |
| S208 | (MH "Coronavirus Infections/TM") | Search modes - Find all my search terms | Interface - EBSCOhost Research Databases  Search Screen - Advanced Search  Database - CINAHL Plus with Full Text | 1,064 |
| S207 | (MH "COVID-19/TM") | Search modes - Find all my search terms | Interface - EBSCOhost Research Databases  Search Screen - Advanced Search  Database - CINAHL Plus with Full Text | 1,170 |
| S206 | (MH "Common Cold/TM") | Search modes - Find all my search terms | Interface - EBSCOhost Research Databases  Search Screen - Advanced Search  Database - CINAHL Plus with Full Text | 53 |
| S205 | (MH "Influenza, Human+/TM") | Search modes - Find all my search terms | Interface - EBSCOhost Research Databases  Search Screen - Advanced Search  Database - CINAHL Plus with Full Text | 697 |
| S204 | (MH "Disease Transmission, Patient-to-Professional") | Search modes - Find all my search terms | Interface - EBSCOhost Research Databases  Search Screen - Advanced Search  Database - CINAHL Plus with Full Text | 1,117 |
| S203 | (MH "Disease Transmission, Horizontal") | Search modes - Find all my search terms | Interface - EBSCOhost Research Databases  Search Screen - Advanced Search  Database - CINAHL Plus with Full Text | 901 |
| S202 | (MH "Cross Infection/TM") | Search modes - Find all my search terms | Interface - EBSCOhost Research Databases  Search Screen - Advanced Search  Database - CINAHL Plus with Full Text | 1,110 |
| S201 | S152 AND S200 | Search modes - Find all my search terms | Interface - EBSCOhost Research Databases  Search Screen - Advanced Search  Database - CINAHL Plus with Full Text | 1,495 |
| S200 | S153 OR S154 OR S155 OR S156 OR S157 OR S158 OR S159 OR S160 OR S161 OR S162 OR S163 OR S164 OR S165 OR S166 OR S167 OR S168 OR S169 OR S170 OR S171 OR S172 OR S173 OR S174 OR S175 OR S176 OR S177 OR S178 OR S179 OR S180 OR S181 OR S182 OR S183 OR S184 OR S185 OR S186 OR S187 OR S188 OR S189 OR S190 OR S191 OR S192 OR S193 OR S194 OR S195 OR S196 OR S197 OR S198 OR S199 | Search modes - Find all my search terms | Interface - EBSCOhost Research Databases  Search Screen - Advanced Search  Database - CINAHL Plus with Full Text | 127,978 |
| S199 | TI ( pneumonia* N3 (viral* or virus*) ) OR AB ( pneumonia* N3 (viral* or virus*) ) | Search modes - Find all my search terms | Interface - EBSCOhost Research Databases  Search Screen - Advanced Search  Database - CINAHL Plus with Full Text | 785 |
| S198 | (MH "Pneumonia, Viral") | Search modes - Find all my search terms | Interface - EBSCOhost Research Databases  Search Screen - Advanced Search  Database - CINAHL Plus with Full Text | 9,371 |
| S197 | TI ( RTI or RTIs or URTI or URTIs or LRTI or LRTIs ) OR AB ( RTI or RTIs or URTI or URTIs or LRTI or LRTIs ) | Search modes - Find all my search terms | Interface - EBSCOhost Research Databases  Search Screen - Advanced Search  Database - CINAHL Plus with Full Text | 1,693 |
| S196 | TI ( (ARI or ARIs) N5 (acute or respirator* or infect* or illness*) ) OR AB ( (ARI or ARIs) N5 (acute or respirator* or infect* or illness*) ) | Search modes - Find all my search terms | Interface - EBSCOhost Research Databases  Search Screen - Advanced Search  Database - CINAHL Plus with Full Text | 458 |
| S195 | TI ( respiratory N3 (infectio* or illness* or virus*) ) OR AB ( respiratory N3 (infectio* or illness* or virus*) ) | Search modes - Find all my search terms | Interface - EBSCOhost Research Databases  Search Screen - Advanced Search  Database - CINAHL Plus with Full Text | 16,830 |
| S194 | (MH "Respiratory Tract Infections") | Search modes - Find all my search terms | Interface - EBSCOhost Research Databases  Search Screen - Advanced Search  Database - CINAHL Plus with Full Text | 8,969 |
| S193 | TI ( parainfluenza* or (para W0 influenza*) ) OR AB ( parainfluenza* or (para W0 influenza*) ) | Search modes - Find all my search terms | Interface - EBSCOhost Research Databases  Search Screen - Advanced Search  Database - CINAHL Plus with Full Text | 474 |
| S192 | TI ( HRSV or HRSVs or RSV or RSVs or sync?tial virus* or sync?tial pneumovirus* or sync?tial pneumo-virus* ) OR AB ( HRSV or HRSVs or RSV or RSVs or sync?tial virus* or sync?tial pneumovirus* or sync?tial pneumo-virus* ) | Search modes - Find all my search terms | Interface - EBSCOhost Research Databases  Search Screen - Advanced Search  Database - CINAHL Plus with Full Text | 2,903 |
| S191 | TI ( (respiratory N1 sync?tial N2 (virus* or pneumovir* or pneumo-vir*) ) OR AB ( (respiratory N1 sync?tial N2 (virus* or pneumovir* or pneumo-vir*) ) | Search modes - Find all my search terms | Interface - EBSCOhost Research Databases  Search Screen - Advanced Search  Database - CINAHL Plus with Full Text | 2,382 |
| S190 | (MH "Respiratory Syncytial Virus Infections") | Search modes - Find all my search terms | Interface - EBSCOhost Research Databases  Search Screen - Advanced Search  Database - CINAHL Plus with Full Text | 2,035 |
| S189 | (MH "Respiratory Syncytial Viruses") | Search modes - Find all my search terms | Interface - EBSCOhost Research Databases  Search Screen - Advanced Search  Database - CINAHL Plus with Full Text | 1,081 |
| S188 | TI ( pneumovirus* or (pneumo W0 virus*) ) OR AB ( pneumovirus* or (pneumo W0 virus*) ) | Search modes - Find all my search terms | Interface - EBSCOhost Research Databases  Search Screen - Advanced Search  Database - CINAHL Plus with Full Text | 6 |
| S187 | TI MERS OR AB MERS | Search modes - Find all my search terms | Interface - EBSCOhost Research Databases  Search Screen - Advanced Search  Database - CINAHL Plus with Full Text | 1,324 |
| S186 | TI "middle east" N2 (respiratory W0 syndrome*) OR AB "middle east" N2 (respiratory W0 syndrome*) | Search modes - Find all my search terms | Interface - EBSCOhost Research Databases  Search Screen - Advanced Search  Database - CINAHL Plus with Full Text | 818 |
| S185 | (MH "Middle East Respiratory Syndrome Coronavirus") | Search modes - Find all my search terms | Interface - EBSCOhost Research Databases  Search Screen - Advanced Search  Database - CINAHL Plus with Full Text | 448 |
| S184 | TI SARS AND AB SARS | Search modes - Find all my search terms | Interface - EBSCOhost Research Databases  Search Screen - Advanced Search  Database - CINAHL Plus with Full Text | 466 |
| S183 | TI ( ("severe acute" or "acute severe") N2 respiratory W0 syndrome* ) OR AB ( ("severe acute" or "acute severe") N2 respiratory W0 syndrome* ) | Search modes - Find all my search terms | Interface - EBSCOhost Research Databases  Search Screen - Advanced Search  Database - CINAHL Plus with Full Text | 4,790 |
| S182 | (MH "Severe Acute Respiratory Syndrome") | Search modes - Find all my search terms | Interface - EBSCOhost Research Databases  Search Screen - Advanced Search  Database - CINAHL Plus with Full Text | 2,480 |
| S181 | (MH "SARS Virus") | Search modes - Find all my search terms | Interface - EBSCOhost Research Databases  Search Screen - Advanced Search  Database - CINAHL Plus with Full Text | 360 |
| S180 | TI ( ((alpha or beta or delta or eta or gamma or iota or kappa or lambda) N3 variant#) and (coronavirus* or (corona W0 virus*) or covid*)) ) OR AB ( ((alpha or beta or delta or eta or gamma or iota or kappa or lambda) N3 variant#) and (coronavirus* or (corona W0 virus*) or covid*)) ) | Search modes - Find all my search terms | Interface - EBSCOhost Research Databases  Search Screen - Advanced Search  Database - CINAHL Plus with Full Text | 14 |
| S179 | TI ( "P.1" and (Brazil* or variant#) ) OR AB ( "P.1" and (Brazil* or variant#) ) | Search modes - Find all my search terms | Interface - EBSCOhost Research Databases  Search Screen - Advanced Search  Database - CINAHL Plus with Full Text | 11 |
| S178 | TI ( "B.1.1.7" or "B.1.351" or "B.1.617" or "B.1.427" or "B.1.429" ) OR AB ( "B.1.1.7" or "B.1.351" or "B.1.617" or "B.1.427" or "B.1.429" ) | Search modes - Find all my search terms | Interface - EBSCOhost Research Databases  Search Screen - Advanced Search  Database - CINAHL Plus with Full Text | 64 |
| S177 | TI coronavirus* or (corona W0 virus*) or Covid | Search modes - Find all my search terms | Interface - EBSCOhost Research Databases  Search Screen - Advanced Search  Database - CINAHL Plus with Full Text | 45,758 |
| S176 | TI ( OC43 or NL63 or 229E or HKU1 or HCoV* or Sars-coronavirus* ) OR AB ( OC43 or NL63 or 229E or HKU1 or HCoV* or Sars-coronavirus* ) | Search modes - Find all my search terms | Interface - EBSCOhost Research Databases  Search Screen - Advanced Search  Database - CINAHL Plus with Full Text | 277 |
| S175 | TI ( "coronavirus 2" or "corona virus 2" ) OR AB ( "coronavirus 2" or "corona virus 2" ) | Search modes - Find all my search terms | Interface - EBSCOhost Research Databases  Search Screen - Advanced Search  Database - CINAHL Plus with Full Text | 3,068 |
| S174 | TI ( (coronavirus* or (corona W0 virus*)) N2 ("2019" or "19") ) OR AB ( (coronavirus* or (corona W0 virus*)) N2 ("2019" or "19") ) | Search modes - Find all my search terms | Interface - EBSCOhost Research Databases  Search Screen - Advanced Search  Database - CINAHL Plus with Full Text | 9,431 |
| S173 | TI ( novel W0 (coronavirus* or (corona W0 virus*) or CoV) ) OR AB ( novel W0 (coronavirus* or (corona W0 virus*) or CoV) ) | Search modes - Find all my search terms | Interface - EBSCOhost Research Databases  Search Screen - Advanced Search  Database - CINAHL Plus with Full Text | 2,325 |
| S172 | TI ( ("2019-novel CoV" or "Sars-coronavirus2" or "Sars-coronavirus-2" or ("SARS-like" W0 coronavirus*) or ((novel or new or nouveau) N2 (CoV or nCoV or covid or coronavirus* or "corona virus" or Pandemi*2)) or (coronavirus* and pneumonia)) ) OR AB ( ("2019-novel CoV" or "Sars-coronavirus2" or "Sars-coronavirus-2" or ("SARS-like" W0 coronavirus*) or ((novel or new or nouveau) N2 (CoV or nCoV or covid or coronavirus* or "corona virus" or Pandemi*2)) or (coronavirus* and pneumonia)) ) | Search modes - Find all my search terms | Interface - EBSCOhost Research Databases  Search Screen - Advanced Search  Database - CINAHL Plus with Full Text | 4,389 |
| S171 | TI ( "SARS-CoV-2" or "SARS-CoV2" or "SARSCoV-2" or SARSCoV2 or SARS2 or "SARS-2" or "severe acute respiratory syndrome coronavirus 2" ) OR AB ( "SARS-CoV-2" or "SARS-CoV2" or "SARSCoV-2" or SARSCoV2 or SARS2 or "SARS-2" or "severe acute respiratory syndrome coronavirus 2" ) | Search modes - Find all my search terms | Interface - EBSCOhost Research Databases  Search Screen - Advanced Search  Database - CINAHL Plus with Full Text | 9,255 |
| S170 | TI ( nCoV or n-CoV or "CoV 2" or CoV2 ) OR AB ( nCoV or n-CoV or "CoV 2" or CoV2 ) | Search modes - Find all my search terms | Interface - EBSCOhost Research Databases  Search Screen - Advanced Search  Database - CINAHL Plus with Full Text | 164 |
| S169 | TI ( "2019-nCoV" or 19nCoV or 2019nCoV ) OR AB ( "2019-nCoV" or 19nCoV or 2019nCoV ) | Search modes - Find all my search terms | Interface - EBSCOhost Research Databases  Search Screen - Advanced Search  Database - CINAHL Plus with Full Text | 263 |
| S168 | TI wuhan N5 virus* OR AB wuhan N5 virus* | Search modes - Find all my search terms | Interface - EBSCOhost Research Databases  Search Screen - Advanced Search  Database - CINAHL Plus with Full Text | 54 |
| S167 | TI ( (coronavirus* or (corona W0 virus*)) and (hubei or wuhan or beijing or shanghai) ) OR AB ( (coronavirus* or (corona W0 virus*)) and (hubei or wuhan or beijing or shanghai) ) | Search modes - Find all my search terms | Interface - EBSCOhost Research Databases  Search Screen - Advanced Search  Database - CINAHL Plus with Full Text | 1,048 |
| S166 | TI ( "COVID-19" or COVID19 ) OR AB ( "COVID-19" or COVID19 ) | Search modes - Find all my search terms | Interface - EBSCOhost Research Databases  Search Screen - Advanced Search  Database - CINAHL Plus with Full Text | 52,288 |
| S165 | (MH "Coronavirus Infections+") | Search modes - Find all my search terms | Interface - EBSCOhost Research Databases  Search Screen - Advanced Search  Database - CINAHL Plus with Full Text | 30,210 |
| S164 | (MH "Coronavirus") | Search modes - Find all my search terms | Interface - EBSCOhost Research Databases  Search Screen - Advanced Search  Database - CINAHL Plus with Full Text | 996 |
| S163 | (MH "SARS-CoV-2") | Search modes - Find all my search terms | Interface - EBSCOhost Research Databases  Search Screen - Advanced Search  Database - CINAHL Plus with Full Text | 511 |
| S162 | (MH "COVID-19") | Search modes - Find all my search terms | Interface - EBSCOhost Research Databases  Search Screen - Advanced Search  Database - CINAHL Plus with Full Text | 18,480 |
| S161 | TI coryza | Search modes - Find all my search terms | Interface - EBSCOhost Research Databases  Search Screen - Advanced Search  Database - CINAHL Plus with Full Text | 0 |
| S160 | TI ( "common cold" or colds ) OR AB ( "common cold" or colds ) | Search modes - Find all my search terms | Interface - EBSCOhost Research Databases  Search Screen - Advanced Search  Database - CINAHL Plus with Full Text | 16,141 |
| S159 | (MH "Common Cold") | Search modes - Find all my search terms | Interface - EBSCOhost Research Databases  Search Screen - Advanced Search  Database - CINAHL Plus with Full Text | 2,762 |
| S158 | TI ( H1N1 or PH1N1 or H3N2 or AH1N1 or "A(H1N1)" or "A/H1N1" or AH3N2 or "A(H3N2)" or "A/H3N2" or H5N1 ) OR AB ( H1N1 or PH1N1 or H3N2 or AH1N1 or "A(H1N1)" or "A/H1N1" or AH3N2 or "A(H3N2)" or "A/H3N2" or H5N1 ) | Search modes - Find all my search terms | Interface - EBSCOhost Research Databases  Search Screen - Advanced Search  Database - CINAHL Plus with Full Text | 5,636 |
| S157 | TI ( influenza* or flu or grippe ) OR AB ( influenza* or flu or grippe ) | Search modes - Find all my search terms | Interface - EBSCOhost Research Databases  Search Screen - Advanced Search  Database - CINAHL Plus with Full Text | 28,400 |
| S156 | (MH "Influenzavirus C") | Search modes - Find all my search terms | Interface - EBSCOhost Research Databases  Search Screen - Advanced Search  Database - CINAHL Plus with Full Text | 12 |
| S155 | (MH "Influenza B Virus") | Search modes - Find all my search terms | Interface - EBSCOhost Research Databases  Search Screen - Advanced Search  Database - CINAHL Plus with Full Text | 466 |
| S154 | (MH "Influenza A Virus") | Search modes - Find all my search terms | Interface - EBSCOhost Research Databases  Search Screen - Advanced Search  Database - CINAHL Plus with Full Text | 1,890 |
| S153 | (MH "Influenza, Human") | Search modes - Find all my search terms | Interface - EBSCOhost Research Databases  Search Screen - Advanced Search  Database - CINAHL Plus with Full Text | 6,732 |
| S152 | S127 AND S151 | Search modes - Find all my search terms | Interface - EBSCOhost Research Databases  Search Screen - Advanced Search  Database - CINAHL Plus with Full Text | 23,975 |
| S151 | S148 OR S149 OR S150 | Search modes - Find all my search terms | Interface - EBSCOhost Research Databases  Search Screen - Advanced Search  Database - CINAHL Plus with Full Text | 1,232,926 |
| S150 | (MH "Health Personnel+") | Search modes - Find all my search terms | Interface - EBSCOhost Research Databases  Search Screen - Advanced Search  Database - CINAHL Plus with Full Text | 606,565 |
| S149 | TI ( allergist* or an#esthet#ist* or an#esthesia assistant* or an#esthesiologist* or audiologist* or cardiologist* or dental technician* or dentist* or dermatologist* or doctor* or emergency medical technician* or endocrinologist* or endodontist* or exodontist* or gastroenterologist* or gastro-enterologist* or (general W0 practitioner*) or geriatrician* or gyn#ecologist* or hospitalist* or nephrologist* or neurologist* or nurse* or obstetrician* or oncologist* or ophthalmologist* or orthodontist* or osteopath or osteopaths or otolaryngologist* or paramedic* or pathologist* or p#ediatrician* or periodontist* or physiatrist* or physician* or physiotherapist* or physio-therapist* or prosthodontist* or pulmonologist* or radiologist* or respiratory technician* or rheumatologist* or surgeon* or therapist* or urologist* ) OR AB ( allergist* or an#esthet#ist* or an#esthesia assistant* or an#esthesiologist* or audiologist* or cardiologist* or dental technician* or dentist* or dermatologist* or doctor* or emergency medical technician* or endocrinologist* or endodontist* or exodontist* or gastroenterologist* or gastro-enterologist* or (general W0 practitioner*) or geriatrician* or gyn#ecologist* or hospitalist* or nephrologist* or neurologist* or nurse* or obstetrician* or oncologist* or ophthalmologist* or orthodontist* or osteopath or osteopaths or otolaryngologist* or paramedic* or pathologist* or p#ediatrician* or periodontist* or physiatrist* or physician* or physiotherapist* or physio-therapist* or prosthodontist* or pulmonologist* or radiologist* or respiratory technician* or rheumatologist* or surgeon* or therapist* or urologist* ) | Search modes - Find all my search terms | Interface - EBSCOhost Research Databases  Search Screen - Advanced Search  Database - CINAHL Plus with Full Text | 765,443 |
| S148 | TI ( (“allied health” or health or “health care” or healthcare or hospital or medical or nursing or “critical care” or “intensive care” or ICU or respiratory or emergency) N3 (assistant* or employee* or personnel or professional* or staff or worker*) ) OR AB ( (“allied health” or health or “health care” or healthcare or hospital or medical or nursing or “critical care” or “intensive care” or ICU or respiratory or emergency) N3 (assistant* or employee* or personnel or professional* or staff or worker*) ) | Search modes - Find all my search terms | Interface - EBSCOhost Research Databases  Search Screen - Advanced Search  Database - CINAHL Plus with Full Text | 182,044 |
| S147 | S127 AND S146 | Search modes - Find all my search terms | Interface - EBSCOhost Research Databases  Search Screen - Advanced Search  Database - CINAHL Plus with Full Text | 486 |
| S146 | S128 OR S129 OR S130 OR S131 OR S132 OR S133 OR S134 OR S135 OR S136 OR S137 OR S138 OR S139 OR S140 OR S141 OR S142 OR S143 OR S144 OR S145 | Search modes - Find all my search terms | Interface - EBSCOhost Research Databases  Search Screen - Advanced Search  Database - CINAHL Plus with Full Text | 5,520 |
| S145 | TI ( ((disease* or infect* or viral* or virus*) N5 risk* N5 (allergist* or an#esthet#ist* or an#esthesia assistant* or an#esthesiologist* or audiologist* or cardiologist* or dental technician* or dentist* or dermatologist* or doctor* or emergency medical technician* or endocrinologist* or endodontist* or exodontist* or gastroenterologist* or gastro-enterologist* or (general W0 practitioner*) or geriatrician* or gyn#ecologist* or hospitalist* or nephrologist* or neurologist* or nurse* or obstetrician* or oncologist* or ophthalmologist* or orthodontist* or osteopath or osteopaths or otolaryngologist* or paramedic* or pathologist* or p#ediatrician* or periodontist* or physiatrist* or physician* or physiotherapist* or physio-therapist* or prosthodontist* or pulmonologist* or radiologist* or respiratory technician* or rheumatologist* or surgeon* or therapist* or urologist*)) ) OR AB ( ((disease* or infect* or viral* or virus*) N5 risk* N5 (allergist* or an#esthet#ist* or an#esthesia assistant* or an#esthesiologist* or audiologist* or cardiologist* or dental technician* or dentist* or dermatologist* or doctor* or emergency medical technician* or endocrinologist* or endodontist* or exodontist* or gastroenterologist* or gastro-enterologist* or (general W0 practitioner*) or geriatrician* or gyn#ecologist* or hospitalist* or nephrologist* or neurologist* or nurse* or obstetrician* or oncologist* or ophthalmologist* or orthodontist* or osteopath or osteopaths or otolaryngologist* or paramedic* or pathologist* or p#ediatrician* or periodontist* or physiatrist* or physician* or physiotherapist* or physio-therapist* or prosthodontist* or pulmonologist* or radiologist* or respiratory technician* or rheumatologist* or surgeon* or therapist* or urologist*)) ) | Search modes - Find all my search terms | Interface - EBSCOhost Research Databases  Search Screen - Advanced Search  Database - CINAHL Plus with Full Text | 834 |
| S144 | TI ( ((disease* or infect* or viral* or virus*) N3 contact*) and (patient? N5 (allergist* or an#esthet#ist* or an#esthesia assistant* or an#esthesiologist* or audiologist* or cardiologist* or dental technician* or dentist* or dermatologist* or doctor* or emergency medical technician* or endocrinologist* or endodontist* or exodontist* or gastroenterologist* or gastro-enterologist* or (general W0 practitioner*) or geriatrician* or gyn#ecologist* or hospitalist* or nephrologist* or neurologist* or nurse* or obstetrician* or oncologist* or ophthalmologist* or orthodontist* or osteopath or osteopaths or otolaryngologist* or paramedic* or pathologist* or p#ediatrician* or periodontist* or physiatrist* or physician* or physiotherapist* or physio-therapist* or prosthodontist* or pulmonologist* or radiologist* or respiratory technician* or rheumatologist* or surgeon* or therapist* or urologist*)) ) OR AB ( ((disease* or infect* or viral* or virus*) N3 contact*) and (patient? N5 (allergist* or an#esthet#ist* or an#esthesia assistant* or an#esthesiologist* or audiologist* or cardiologist* or dental technician* or dentist* or dermatologist* or doctor* or emergency medical technician* or endocrinologist* or endodontist* or exodontist* or gastroenterologist* or gastro-enterologist* or (general W0 practitioner*) or geriatrician* or gyn#ecologist* or hospitalist* or nephrologist* or neurologist* or nurse* or obstetrician* or oncologist* or ophthalmologist* or orthodontist* or osteopath or osteopaths or otolaryngologist* or paramedic* or pathologist* or p#ediatrician* or periodontist* or physiatrist* or physician* or physiotherapist* or physio-therapist* or prosthodontist* or pulmonologist* or radiologist* or respiratory technician* or rheumatologist* or surgeon* or therapist* or urologist*)) ) | Search modes - Find all my search terms | Interface - EBSCOhost Research Databases  Search Screen - Advanced Search  Database - CINAHL Plus with Full Text | 47 |
| S143 | TI ( ((disease* or infect* or viral* or virus*) N3 expos*) and (patient? N5 (allergist* or an#esthet#ist* or an#esthesia assistant* or an#esthesiologist* or audiologist* or cardiologist* or dental technician* or dentist* or dermatologist* or doctor* or emergency medical technician* or endocrinologist* or endodontist* or exodontist* or gastroenterologist* or gastro-enterologist* or (general W0 practitioner*) or geriatrician* or gyn#ecologist* or hospitalist* or nephrologist* or neurologist* or nurse* or obstetrician* or oncologist* or ophthalmologist* or orthodontist* or osteopath or osteopaths or otolaryngologist* or paramedic* or pathologist* or p#ediatrician* or periodontist* or physiatrist* or physician* or physiotherapist* or physio-therapist* or prosthodontist* or pulmonologist* or radiologist* or respiratory technician* or rheumatologist* or surgeon* or therapist* or urologist*)) ) OR AB ( ((disease* or infect* or viral* or virus*) N3 expos*) and (patient? N5 (allergist* or an#esthet#ist* or an#esthesia assistant* or an#esthesiologist* or audiologist* or cardiologist* or dental technician* or dentist* or dermatologist* or doctor* or emergency medical technician* or endocrinologist* or endodontist* or exodontist* or gastroenterologist* or gastro-enterologist* or (general W0 practitioner*) or geriatrician* or gyn#ecologist* or hospitalist* or nephrologist* or neurologist* or nurse* or obstetrician* or oncologist* or ophthalmologist* or orthodontist* or osteopath or osteopaths or otolaryngologist* or paramedic* or pathologist* or p#ediatrician* or periodontist* or physiatrist* or physician* or physiotherapist* or physio-therapist* or prosthodontist* or pulmonologist* or radiologist* or respiratory technician* or rheumatologist* or surgeon* or therapist* or urologist*)) ) | Search modes - Find all my search terms | Interface - EBSCOhost Research Databases  Search Screen - Advanced Search  Database - CINAHL Plus with Full Text | 84 |
| S142 | TI ( ((disease* or infect* or viral* or virus*) N3 spread*) and (patient? N5 (allergist* or an#esthet#ist* or an#esthesia assistant* or an#esthesiologist* or audiologist* or cardiologist* or dental technician* or dentist* or dermatologist* or doctor* or emergency medical technician* or endocrinologist* or endodontist* or exodontist* or gastroenterologist* or gastro-enterologist* or (general W0 practitioner*) or geriatrician* or gyn#ecologist* or hospitalist* or nephrologist* or neurologist* or nurse* or obstetrician* or oncologist* or ophthalmologist* or orthodontist* or osteopath or osteopaths or otolaryngologist* or paramedic* or pathologist* or p#ediatrician* or periodontist* or physiatrist* or physician* or physiotherapist* or physio-therapist* or prosthodontist* or pulmonologist* or radiologist* or respiratory technician* or rheumatologist* or surgeon* or therapist* or urologist*)) ) OR AB ( ((disease* or infect* or viral* or virus*) N3 spread*) and (patient? N5 (allergist* or an#esthet#ist* or an#esthesia assistant* or an#esthesiologist* or audiologist* or cardiologist* or dental technician* or dentist* or dermatologist* or doctor* or emergency medical technician* or endocrinologist* or endodontist* or exodontist* or gastroenterologist* or gastro-enterologist* or (general W0 practitioner*) or geriatrician* or gyn#ecologist* or hospitalist* or nephrologist* or neurologist* or nurse* or obstetrician* or oncologist* or ophthalmologist* or orthodontist* or osteopath or osteopaths or otolaryngologist* or paramedic* or pathologist* or p#ediatrician* or periodontist* or physiatrist* or physician* or physiotherapist* or physio-therapist* or prosthodontist* or pulmonologist* or radiologist* or respiratory technician* or rheumatologist* or surgeon* or therapist* or urologist*)) ) | Search modes - Find all my search terms | Interface - EBSCOhost Research Databases  Search Screen - Advanced Search  Database - CINAHL Plus with Full Text | 125 |
| S141 | TI ( ((disease* or infect* or viral* or virus*) N3 transmi*) and (patient? N5 (allergist* or an#esthet#ist* or an#esthesia assistant* or an#esthesiologist* or audiologist* or cardiologist* or dental technician* or dentist* or dermatologist* or doctor* or emergency medical technician* or endocrinologist* or endodontist* or exodontist* or gastroenterologist* or gastro-enterologist* or (general W0 practitioner*) or geriatrician* or gyn#ecologist* or hospitalist* or nephrologist* or neurologist* or nurse* or obstetrician* or oncologist* or ophthalmologist* or orthodontist* or osteopath or osteopaths or otolaryngologist* or paramedic* or pathologist* or p#ediatrician* or periodontist* or physiatrist* or physician* or physiotherapist* or physio-therapist* or prosthodontist* or pulmonologist* or radiologist* or respiratory technician* or rheumatologist* or surgeon* or therapist* or urologist*)) ) OR AB ( ((disease* or infect* or viral* or virus*) N3 transmi*) and (patient? N5 (allergist* or an#esthet#ist* or an#esthesia assistant* or an#esthesiologist* or audiologist* or cardiologist* or dental technician* or dentist* or dermatologist* or doctor* or emergency medical technician* or endocrinologist* or endodontist* or exodontist* or gastroenterologist* or gastro-enterologist* or (general W0 practitioner*) or geriatrician* or gyn#ecologist* or hospitalist* or nephrologist* or neurologist* or nurse* or obstetrician* or oncologist* or ophthalmologist* or orthodontist* or osteopath or osteopaths or otolaryngologist* or paramedic* or pathologist* or p#ediatrician* or periodontist* or physiatrist* or physician* or physiotherapist* or physio-therapist* or prosthodontist* or pulmonologist* or radiologist* or respiratory technician* or rheumatologist* or surgeon* or therapist* or urologist*)) ) | Search modes - Find all my search terms | Interface - EBSCOhost Research Databases  Search Screen - Advanced Search  Database - CINAHL Plus with Full Text | 325 |
| S140 | TI ( ((disease* or infect* or viral* or virus*) N3 contact*) and (patient# N5 (assistant# or employee* or personnel or professional# or staff or technician* or worker#)) ) OR AB ( ((disease* or infect* or viral* or virus*) N3 contact*) and (patient# N5 (assistant# or employee* or personnel or professional# or staff or technician* or worker#)) ) | Search modes - Find all my search terms | Interface - EBSCOhost Research Databases  Search Screen - Advanced Search  Database - CINAHL Plus with Full Text | 78 |
| S139 | TI ( ((disease* or infect* or viral* or virus*) N3 expos*) and (patient# N5 (assistant# or employee* or personnel or professional# or staff or technician* or worker#)) ) OR AB ( ((disease* or infect* or viral* or virus*) N3 expos*) and (patient# N5 (assistant# or employee* or personnel or professional# or staff or technician* or worker#)) ) | Search modes - Find all my search terms | Interface - EBSCOhost Research Databases  Search Screen - Advanced Search  Database - CINAHL Plus with Full Text | 137 |
| S138 | TI ( ((disease* or infect* or viral* or virus*) N3 spread*) and (patient# N5 (assistant# or employee* or personnel or professional# or staff or technician* or worker#)) ) OR AB ( ((disease* or infect* or viral* or virus*) N3 spread*) and (patient# N5 (assistant# or employee* or personnel or professional# or staff or technician* or worker#)) ) | Search modes - Find all my search terms | Interface - EBSCOhost Research Databases  Search Screen - Advanced Search  Database - CINAHL Plus with Full Text | 200 |
| S137 | TI ( ((disease* or infect* or viral* or virus*) N3 transmi*) and (patient# N5 (assistant# or employee* or personnel or professional# or staff or technician* or worker#)) ) OR AB ( ((disease* or infect* or viral* or virus*) N3 transmi*) and (patient# N5 (assistant# or employee* or personnel or professional# or staff or technician* or worker#)) ) | Search modes - Find all my search terms | Interface - EBSCOhost Research Databases  Search Screen - Advanced Search  Database - CINAHL Plus with Full Text | 410 |
| S136 | (MH "Disease Transmission, Patient-to-Professional") | Search modes - Find all my search terms | Interface - EBSCOhost Research Databases  Search Screen - Advanced Search  Database - CINAHL Plus with Full Text | 1,116 |
| S135 | TI ( (disease* or infect* or viral* or virus*) N5 risk* N5 (assistant# or employee* or personnel or professional# or staff or technician* or worker#) ) OR AB ( (disease* or infect* or viral* or virus*) N5 risk* N5 (assistant# or employee* or personnel or professional# or staff or technician* or worker#) ) | Search modes - Find all my search terms | Interface - EBSCOhost Research Databases  Search Screen - Advanced Search  Database - CINAHL Plus with Full Text | 1,310 |
| S134 | TI ( nosocomial N5 transmi* N5 (assistant# or employee* or personnel or professional# or staff or technician* or worker#) ) OR AB ( nosocomial N5 transmi* N5 (assistant# or employee* or personnel or professional# or staff or technician* or worker#) ) | Search modes - Find all my search terms | Interface - EBSCOhost Research Databases  Search Screen - Advanced Search  Database - CINAHL Plus with Full Text | 39 |
| S133 | TI ( (nosocomial W0 infection*) N5 (assistant# or employee* or personnel or professional# or staff or technician* or worker#) ) OR AB ( (nosocomial W0 infection*) N5 (assistant# or employee* or personnel or professional# or staff or technician* or worker#) ) | Search modes - Find all my search terms | Interface - EBSCOhost Research Databases  Search Screen - Advanced Search  Database - CINAHL Plus with Full Text | 74 |
| S132 | TI ( (hospital W0 infection*) N5 (assistant# or employee* or personnel or professional# or staff or technician* or worker#) ) OR AB ( (hospital W0 infection*) N5 (assistant# or employee* or personnel or professional# or staff or technician* or worker#) ) | Search modes - Find all my search terms | Interface - EBSCOhost Research Databases  Search Screen - Advanced Search  Database - CINAHL Plus with Full Text | 129 |
| S131 | TI ( (("healthcare associated" W0 transmi*) or ("health care associated" W0 transmi*) N5 (assistant# or employee* or personnel or professional# or staff or technician* or worker#) ) OR AB ( (("healthcare associated" W0 transmi*) or ("health care associated" W0 transmi*) N5 (assistant# or employee* or personnel or professional# or staff or technician* or worker#) ) | Search modes - Find all my search terms | Interface - EBSCOhost Research Databases  Search Screen - Advanced Search  Database - CINAHL Plus with Full Text | 11 |
| S130 | TI ( (("healthcare associated" W0 infection*) or ("health care associated" W0 infection*)) N5 (assistant# or employee* or personnel or professional# or staff or technician* or worker#) ) OR AB ( (("healthcare associated" W0 infection*) or ("health care associated" W0 infection*)) N5 (assistant# or employee* or personnel or professional# or staff or technician* or worker#) ) | Search modes - Find all my search terms | Interface - EBSCOhost Research Databases  Search Screen - Advanced Search  Database - CINAHL Plus with Full Text | 65 |
| S129 | TI ( (cross W0 infection*) N5 (assistant# or employee* or personnel or professional# or staff or technician* or worker#) ) OR AB ( (cross W0 infection*) N5 (assistant# or employee* or personnel or professional# or staff or technician* or worker#) ) | Search modes - Find all my search terms | Interface - EBSCOhost Research Databases  Search Screen - Advanced Search  Database - CINAHL Plus with Full Text | 36 |
| S128 | (MH "Cross Infection/TM") | Search modes - Find all my search terms | Interface - EBSCOhost Research Databases  Search Screen - Advanced Search  Database - CINAHL Plus with Full Text | 1,109 |
| S127 | S1 OR S2 OR S3 OR S4 OR S5 OR S6 OR S7 OR S8 OR S9 OR S10 OR S11 OR S12 OR S13 OR S14 OR S15 OR S16 OR S17 OR S18 OR S19 OR S20 OR S21 OR S22 OR S23 OR S24 OR S25 OR S26 OR S27 OR S28 OR S29 OR S30 OR S31 OR S32 OR S33 OR S34 OR S35 OR S36 OR S37 OR S38 OR S39 OR S40 OR S41 OR S42 OR S43 OR S44 OR S45 OR S46 OR S47 OR S48 OR S49 OR S50 OR S51 OR S52 OR S53 OR S54 OR S55 OR S56 OR S57 OR S58 OR S59 OR S60 OR S61 OR S62 OR S63 OR S64 OR S65 OR S66 OR S67 OR S68 OR S69 OR S70 OR S71 OR S72 OR S73 OR S74 OR S75 OR S76 OR S77 OR S78 OR S79 OR S80 OR S81 OR S82 OR S83 OR S84 OR S85 OR S86 OR S87 OR S88 OR S89 OR S90 OR S91 OR S92 OR S93 OR S94 OR S95 OR S96 OR S97 OR S98 OR S99 OR S100 OR S101 OR S102 OR S103 OR S104 OR S105 OR S106 OR S107 OR S108 OR S109 OR S110 OR S111 OR S112 OR S113 OR S114 OR S115 OR S116 OR S117 OR S118 OR S119 OR S120 OR S121 OR S122 OR S123 OR S124 OR S125 OR S126 | Search modes - Find all my search terms | Interface - EBSCOhost Research Databases  Search Screen - Advanced Search  Database - CINAHL Plus with Full Text | 161,153 |
| S126 | TI ( (secrete* or secreti*) N3 (breath* or cough* or mouth* or mucus* or nasal* or nose# or oral* or nasopharyn* or naso-pharyn* or oropharyn* or oro-pharyn* or pharyn* or respirat* or saliva*) ) OR AB ( (secrete* or secreti*) N3 (breath* or cough* or mouth* or mucus* or nasal* or nose# or oral* or nasopharyn* or naso-pharyn* or oropharyn* or oro-pharyn* or pharyn* or respirat* or saliva*) ) | Search modes - Find all my search terms | Interface - EBSCOhost Research Databases  Search Screen - Advanced Search  Database - CINAHL Plus with Full Text | 1,850 |
| S125 | TI droplet# OR AB droplet# | Search modes - Find all my search terms | Interface - EBSCOhost Research Databases  Search Screen - Advanced Search  Database - CINAHL Plus with Full Text | 2,200 |
| S124 | TI ( (VQ W0 scinti-photo*) or ("V/Q" W0 scinti-photo*) or (ventilation N1 perfusion N3 scinti-photo*) or (lung N1 perfusion N3 scinti-photo*) or (pulmonary N1 perfusion N3 scinti-photo*) ) OR AB ( (VQ W0 scinti-photo*) or ("V/Q" W0 scinti-photo*) or (ventilation N1 perfusion N3 scinti-photo*) or (lung N1 perfusion N3 scinti-photo*) or (pulmonary N1 perfusion N3 scinti-photo*) ) | Search modes - Find all my search terms | Interface - EBSCOhost Research Databases  Search Screen - Advanced Search  Database - CINAHL Plus with Full Text | 0 |
| S123 | TI ( (VQ W0 scintiphoto*) or ("V/Q" W0 scintiphoto*) or (ventilation N1 perfusion N3 scintiphoto*) or (lung N1 perfusion N3 scintiphoto*) or (pulmonary N1 perfusion N3 scintiphoto*) ) OR AB ( (VQ W0 scintiphoto*) or ("V/Q" W0 scintiphoto*) or (ventilation N1 perfusion N3 scintiphoto*) or (lung N1 perfusion N3 scintiphoto*) or (pulmonary N1 perfusion N3 scintiphoto*) ) | Search modes - Find all my search terms | Interface - EBSCOhost Research Databases  Search Screen - Advanced Search  Database - CINAHL Plus with Full Text | 2 |
| S122 | TI ( (VQ W0 scintigram*) or ("V/Q" W0 scintigram*) or (ventilation N1 perfusion N3 scintigram*) or (lung N1 perfusion N3 scintigram*) or (pulmonary N1 perfusion N3 scintigram*) ) OR AB ( (VQ W0 scintigram*) or ("V/Q" W0 scintigram*) or (ventilation N1 perfusion N3 scintigram*) or (lung N1 perfusion N3 scintigram*) or (pulmonary N1 perfusion N3 scintigram*) ) | Search modes - Find all my search terms | Interface - EBSCOhost Research Databases  Search Screen - Advanced Search  Database - CINAHL Plus with Full Text | 1 |
| S121 | TI ( (VQ W0 scintiscan*) or ("V/Q" W0 scintiscan*) or (ventilation N1 perfusion N3 scintiscan*) or (lung N1 perfusion N3 scintiscan*) or (pulmonary N1 perfusion N3 scintiscan*) ) OR AB ( (VQ W0 scintiscan*) or ("V/Q" W0 scintiscan*) or (ventilation N1 perfusion N3 scintiscan*) or (lung N1 perfusion N3 scintiscan*) or (pulmonary N1 perfusion N3 scintiscan*) ) | Search modes - Find all my search terms | Interface - EBSCOhost Research Databases  Search Screen - Advanced Search  Database - CINAHL Plus with Full Text | 3 |
| S120 | TI ( (VQ W0 scintigraph*) or ("V/Q" W0 scintigraph*) or (ventilation N1 perfusion N3 scintigraph*) or (lung N1 perfusion N3 scintigraph*) or (pulmonary N1 perfusion N3 scintigraph*) ) OR AB ( (VQ W0 scintigraph*) or ("V/Q" W0 scintigraph*) or (ventilation N1 perfusion N3 scintigraph*) or (lung N1 perfusion N3 scintigraph*) or (pulmonary N1 perfusion N3 scintigraph*) ) | Search modes - Find all my search terms | Interface - EBSCOhost Research Databases  Search Screen - Advanced Search  Database - CINAHL Plus with Full Text | 168 |
| S119 | TI ( (VQ W0 scan*) or ("V/Q" W0 scan*) or (ventilation N1 perfusion N3 scan*) or (lung N1 perfusion N3 scan*) or (pulmonary N1 perfusion N3 scan*) ) OR AB ( (VQ W0 scan*) or ("V/Q" W0 scan*) or (ventilation N1 perfusion N3 scan*) or (lung N1 perfusion N3 scan*) or (pulmonary N1 perfusion N3 scan*) ) | Search modes - Find all my search terms | Interface - EBSCOhost Research Databases  Search Screen - Advanced Search  Database - CINAHL Plus with Full Text | 460 |
| S118 | (MH "Perfusion Imaging") | Search modes - Find all my search terms | Interface - EBSCOhost Research Databases  Search Screen - Advanced Search  Database - CINAHL Plus with Full Text | 1,481 |
| S117 | TI ( (oral* or spray*) N5 lidocaine* ) OR AB ( (oral* or spray*) N5 lidocaine* ) | Search modes - Find all my search terms | Interface - EBSCOhost Research Databases  Search Screen - Advanced Search  Database - CINAHL Plus with Full Text | 187 |
| S116 | TI ( cold W0 (analg#esi* or an#esthe*) ) OR AB ( cold W0 (analg#esi* or an#esthe*) ) | Search modes - Find all my search terms | Interface - EBSCOhost Research Databases  Search Screen - Advanced Search  Database - CINAHL Plus with Full Text | 6 |
| S115 | TI ( cryoan#esthesi* or (cryo W0 an#esthesi*) or cryoanalgesi* or (cryo W0 analgesi*) ) OR AB ( cryoan#esthesi* or (cryo W0 an#esthesi*) or cryoanalgesi* or (cryo W0 analgesi*) ) | Search modes - Find all my search terms | Interface - EBSCOhost Research Databases  Search Screen - Advanced Search  Database - CINAHL Plus with Full Text | 52 |
| S114 | TI ( (analg#esi* or an#esthe*) and (cryo* or freez* or frozen*) and (agent* or spray*) ) OR AB ( (analg#esi* or an#esthe*) and (cryo* or freez* or frozen*) and (agent* or spray*) ) | Search modes - Find all my search terms | Interface - EBSCOhost Research Databases  Search Screen - Advanced Search  Database - CINAHL Plus with Full Text | 36 |
| S113 | TI ( compress* N1 (gas or gases or gasses) N5 (administ* or deliver* or method* or treat* or therap*) ) OR AB ( compress* N1 (gas or gases or gasses) N5 (administ* or deliver* or method* or treat* or therap*) ) | Search modes - Find all my search terms | Interface - EBSCOhost Research Databases  Search Screen - Advanced Search  Database - CINAHL Plus with Full Text | 7 |
| S112 | TI propellant* OR AB propellant* | Search modes - Find all my search terms | Interface - EBSCOhost Research Databases  Search Screen - Advanced Search  Database - CINAHL Plus with Full Text | 144 |
| S111 | (MH "Sphenoid Sinus/SU") | Search modes - Find all my search terms | Interface - EBSCOhost Research Databases  Search Screen - Advanced Search  Database - CINAHL Plus with Full Text | 177 |
| S110 | (MH "Sphenoid Bone/SU") | Search modes - Find all my search terms | Interface - EBSCOhost Research Databases  Search Screen - Advanced Search  Database - CINAHL Plus with Full Text | 158 |
| S109 | TI ( (transsphenoid* or trans-sphenoid*) W0 (surger* or surgical*) ) OR AB ( (transsphenoid* or trans-sphenoid*) W0 (surger* or surgical*) ) | Search modes - Find all my search terms | Interface - EBSCOhost Research Databases  Search Screen - Advanced Search  Database - CINAHL Plus with Full Text | 266 |
| S108 | TI ( PAxpress* or SLIPA or SLIPAR or SLIPATM ) OR AB ( PAxpress* or SLIPA or SLIPAR or SLIPATM ) | Search modes - Find all my search terms | Interface - EBSCOhost Research Databases  Search Screen - Advanced Search  Database - CINAHL Plus with Full Text | 22 |
| S107 | TI ( "King LT" or "King LTR" or "King LTTM" ) OR AB ( "King LT" or "King LTR" or "King LTTM" ) | Search modes - Find all my search terms | Interface - EBSCOhost Research Databases  Search Screen - Advanced Search  Database - CINAHL Plus with Full Text | 29 |
| S106 | TI ( "I-GEL" or "I-GELR" or "I-GELTM" ) OR AB ( "I-GEL" or "I-GELR" or "I-GELTM" ) | Search modes - Find all my search terms | Interface - EBSCOhost Research Databases  Search Screen - Advanced Search  Database - CINAHL Plus with Full Text | 230 |
| S105 | TI ( LMA and (supraglottic or "supra-glottic") ) OR AB ( LMA and (supraglottic or "supra-glottic") ) | Search modes - Find all my search terms | Interface - EBSCOhost Research Databases  Search Screen - Advanced Search  Database - CINAHL Plus with Full Text | 91 |
| S104 | (MH "Airway Management") AND (MH "Laryngeal Masks") | Search modes - Find all my search terms | Interface - EBSCOhost Research Databases  Search Screen - Advanced Search  Database - CINAHL Plus with Full Text | 410 |
| S103 | TI laryngeal W0 airway* OR AB laryngeal W0 airway* | Search modes - Find all my search terms | Interface - EBSCOhost Research Databases  Search Screen - Advanced Search  Database - CINAHL Plus with Full Text | 82 |
| S102 | TI ( (supraglottic or "supra-glottic") W0 airway* ) OR AB ( (supraglottic or "supra-glottic") W0 airway* ) | Search modes - Find all my search terms | Interface - EBSCOhost Research Databases  Search Screen - Advanced Search  Database - CINAHL Plus with Full Text | 522 |
| S101 | TI ( sputum* or expectorat* or spit* ) OR AB ( sputum* or expectorat* or spit* ) | Search modes - Find all my search terms | Interface - EBSCOhost Research Databases  Search Screen - Advanced Search  Database - CINAHL Plus with Full Text | 14,111 |
| S100 | (MH "Sputum") | Search modes - Find all my search terms | Interface - EBSCOhost Research Databases  Search Screen - Advanced Search  Database - CINAHL Plus with Full Text | 3,257 |
| S99 | TI ( laryngectom* or (laryn W0 gectom*) ) OR AB ( laryngectom* or (laryn W0 gectom*) ) | Search modes - Find all my search terms | Interface - EBSCOhost Research Databases  Search Screen - Advanced Search  Database - CINAHL Plus with Full Text | 1,675 |
| S98 | (MH "Laryngectomy") | Search modes - Find all my search terms | Interface - EBSCOhost Research Databases  Search Screen - Advanced Search  Database - CINAHL Plus with Full Text | 2,002 |
| S97 | TI tracheostom* OR AB tracheostom* | Search modes - Find all my search terms | Interface - EBSCOhost Research Databases  Search Screen - Advanced Search  Database - CINAHL Plus with Full Text | 4,830 |
| S96 | (MH "Tracheostomy") | Search modes - Find all my search terms | Interface - EBSCOhost Research Databases  Search Screen - Advanced Search  Database - CINAHL Plus with Full Text | 4,656 |
| S95 | TI ( nasopharyngoscop* or (naso W0 pharyngoscop*) or (fiberoptic* W0 nasendoscop*) or (fiber W0 optic* W0 nasendoscop*) or (fibreoptic* W0 nasendoscop*) or (fibre W0 optic* W0 nasendoscop*) or nasolaryngoscop* or (naso W0 laryngoscop*) or nasopharyngolaryngoscop* or (naso W0 pharyngolaryngoscop*) or (flexible N2 laryngoscop*) ) OR AB ( nasopharyngoscop* or (naso W0 pharyngoscop*) or (fiberoptic* W0 nasendoscop*) or (fiber W0 optic* W0 nasendoscop*) or (fibreoptic* W0 nasendoscop*) or (fibre W0 optic* W0 nasendoscop*) or nasolaryngoscop* or (naso W0 laryngoscop*) or nasopharyngolaryngoscop* or (naso W0 pharyngolaryngoscop*) or (flexible N2 laryngoscop*) ) | Search modes - Find all my search terms | Interface - EBSCOhost Research Databases  Search Screen - Advanced Search  Database - CINAHL Plus with Full Text | 453 |
| S94 | TI ( support* N3 (ventilat* or resuscitat*) ) OR AB ( support* N3 (ventilat* or resuscitat*) ) | Search modes - Find all my search terms | Interface - EBSCOhost Research Databases  Search Screen - Advanced Search  Database - CINAHL Plus with Full Text | 4,405 |
| S93 | TI mechanical* N3 resuscitat* OR AB mechanical* N3 resuscitat* | Search modes - Find all my search terms | Interface - EBSCOhost Research Databases  Search Screen - Advanced Search  Database - CINAHL Plus with Full Text | 265 |
| S92 | TI mechanical* N3 ventilat* OR AB mechanical* N3 ventilat* | Search modes - Find all my search terms | Interface - EBSCOhost Research Databases  Search Screen - Advanced Search  Database - CINAHL Plus with Full Text | 20,741 |
| S91 | (MH "Ventilators, Mechanical") | Search modes - Find all my search terms | Interface - EBSCOhost Research Databases  Search Screen - Advanced Search  Database - CINAHL Plus with Full Text | 3,160 |
| S90 | TI "T-piece" N3 resuscitat* OR AB "T-piece" N3 resuscitat* | Search modes - Find all my search terms | Interface - EBSCOhost Research Databases  Search Screen - Advanced Search  Database - CINAHL Plus with Full Text | 60 |
| S89 | TI ( (selfinflat* or (self W0 inflat*) or flowinflat* or (flow W0 inflat*)) N3 resuscitat* ) OR AB ( (selfinflat* or (self W0 inflat*) or flowinflat* or (flow W0 inflat*)) N3 resuscitat* ) | Search modes - Find all my search terms | Interface - EBSCOhost Research Databases  Search Screen - Advanced Search  Database - CINAHL Plus with Full Text | 53 |
| S88 | TI manual* N3 resuscitat* OR AB manual* N3 resuscitat* | Search modes - Find all my search terms | Interface - EBSCOhost Research Databases  Search Screen - Advanced Search  Database - CINAHL Plus with Full Text | 190 |
| S87 | TI manual* N3 ventilat* OR AB manual* N3 ventilat* | Search modes - Find all my search terms | Interface - EBSCOhost Research Databases  Search Screen - Advanced Search  Database - CINAHL Plus with Full Text | 294 |
| S86 | TI artificial* N3 respirat* OR AB artificial* N3 respirat* | Search modes - Find all my search terms | Interface - EBSCOhost Research Databases  Search Screen - Advanced Search  Database - CINAHL Plus with Full Text | 129 |
| S85 | TI ( AIRVO* or Optiflow* or Vapotherm* ) OR AB ( AIRVO* or Optiflow* or Vapotherm* ) | Search modes - Find all my search terms | Interface - EBSCOhost Research Databases  Search Screen - Advanced Search  Database - CINAHL Plus with Full Text | 79 |
| S84 | (MH "Respiration, Artificial") | Search modes - Find all my search terms | Interface - EBSCOhost Research Databases  Search Screen - Advanced Search  Database - CINAHL Plus with Full Text | 0 |
| S83 | TI ( intubat* or extubat* or detubat* ) OR AB ( intubat* or extubat* or detubat* ) | Search modes - Find all my search terms | Interface - EBSCOhost Research Databases  Search Screen - Advanced Search  Database - CINAHL Plus with Full Text | 21,758 |
| S82 | (MH "Extubation") | Search modes - Find all my search terms | Interface - EBSCOhost Research Databases  Search Screen - Advanced Search  Database - CINAHL Plus with Full Text | 1,140 |
| S81 | (MH "Intubation+") | Search modes - Find all my search terms | Interface - EBSCOhost Research Databases  Search Screen - Advanced Search  Database - CINAHL Plus with Full Text | 19,354 |
| S80 | TI ( ((non W0 rebreath* W0 mask*) or (nonrebreath* W0 mask*) or NRBM or NRBMs)) and (nasal can#ula* or "nose tube" or "nose tubes" or "nasal tube" or "nasal tubes")) ) OR AB ( ((non W0 rebreath* W0 mask*) or (nonrebreath* W0 mask*) or NRBM or NRBMs)) and (nasal can#ula* or "nose tube" or "nose tubes" or "nasal tube" or "nasal tubes")) ) | Search modes - Find all my search terms | Interface - EBSCOhost Research Databases  Search Screen - Advanced Search  Database - CINAHL Plus with Full Text | 17 |
| S79 | TI ( (oxygen* or O2) N5 (nonhumid* or non-humid* or dry) ) OR AB ( (oxygen* or O2) N5 (nonhumid* or non-humid* or dry) ) | Search modes - Find all my search terms | Interface - EBSCOhost Research Databases  Search Screen - Advanced Search  Database - CINAHL Plus with Full Text | 42 |
| S78 | TI Venturi* OR AB Venturi* | Search modes - Find all my search terms | Interface - EBSCOhost Research Databases  Search Screen - Advanced Search  Database - CINAHL Plus with Full Text | 283 |
| S77 | TI ( (oxygen* or O2) N10 (flush W0 flow*) ) OR AB ( (oxygen* or O2) N10 (flush W0 flow*) ) | Search modes - Find all my search terms | Interface - EBSCOhost Research Databases  Search Screen - Advanced Search  Database - CINAHL Plus with Full Text | 1 |
| S76 | TI ( (HHHFO or HHFO) and (heat* or humid* or "high flow" or highflow or oxygen* or O2) ) OR AB ( (HHHFO or HHFO) and (heat* or humid* or "high flow" or highflow or oxygen* or O2) ) | Search modes - Find all my search terms | Interface - EBSCOhost Research Databases  Search Screen - Advanced Search  Database - CINAHL Plus with Full Text | 0 |
| S75 | TI ( ("high flow" or highflow) N5 (oxygen* or O2 or nasal* or can#ul*) ) OR AB ( ("high flow" or highflow) N5 (oxygen* or O2 or nasal* or can#ul*) ) | Search modes - Find all my search terms | Interface - EBSCOhost Research Databases  Search Screen - Advanced Search  Database - CINAHL Plus with Full Text | 1,468 |
| S74 | (MH "Oxygen Therapy+") | Search modes - Find all my search terms | Interface - EBSCOhost Research Databases  Search Screen - Advanced Search  Database - CINAHL Plus with Full Text | 9,524 |
| S73 | TI HFV N3 oscillat* OR AB HFV N3 oscillat* | Search modes - Find all my search terms | Interface - EBSCOhost Research Databases  Search Screen - Advanced Search  Database - CINAHL Plus with Full Text | 4 |
| S72 | TI ( (HFV or HFJV or HFOV) N10 ventilat* ) OR AB ( (HFV or HFJV or HFOV) N10 ventilat* ) | Search modes - Find all my search terms | Interface - EBSCOhost Research Databases  Search Screen - Advanced Search  Database - CINAHL Plus with Full Text | 365 |
| S71 | TI ( (highfrequen* or (high W0 frequen*)) N3 (ventilat* or oscillat*) ) OR AB ( (highfrequen* or (high W0 frequen*)) N3 (ventilat* or oscillat*) ) | Search modes - Find all my search terms | Interface - EBSCOhost Research Databases  Search Screen - Advanced Search  Database - CINAHL Plus with Full Text | 1,568 |
| S70 | (MH "Ventilation, High Frequency+") | Search modes - Find all my search terms | Interface - EBSCOhost Research Databases  Search Screen - Advanced Search  Database - CINAHL Plus with Full Text | 1,197 |
| S69 | TI aspirat* N3 mechanical* OR AB aspirat* N3 mechanical* | Search modes - Find all my search terms | Interface - EBSCOhost Research Databases  Search Screen - Advanced Search  Database - CINAHL Plus with Full Text | 119 |
| S68 | TI suction* OR AB suction* | Search modes - Find all my search terms | Interface - EBSCOhost Research Databases  Search Screen - Advanced Search  Database - CINAHL Plus with Full Text | 4,036 |
| S67 | (MH "Suction+") | Search modes - Find all my search terms | Interface - EBSCOhost Research Databases  Search Screen - Advanced Search  Database - CINAHL Plus with Full Text | 3,520 |
| S66 | TI ( cryostat* or cryomicrotome* or (cryo W0 microtome*) or (freezing W0 microtome*) ) OR AB ( cryostat* or cryomicrotome* or (cryo W0 microtome*) or (freezing W0 microtome*) ) | Search modes - Find all my search terms | Interface - EBSCOhost Research Databases  Search Screen - Advanced Search  Database - CINAHL Plus with Full Text | 93 |
| S65 | TI ( frozen N2 (section* or specimen* or tissue*) N3 (biops* or cut or cuts or cutting* or knife# or knives or shave or shaved or shaves or shaving* or microtome*) ) OR AB ( frozen N2 (section* or specimen* or tissue*) N3 (biops* or cut or cuts or cutting* or knife# or knives or shave or shaved or shaves or shaving* or microtome*) ) | Search modes - Find all my search terms | Interface - EBSCOhost Research Databases  Search Screen - Advanced Search  Database - CINAHL Plus with Full Text | 129 |
| S64 | (MH "Frozen Sections/MT") | Search modes - Find all my search terms | Interface - EBSCOhost Research Databases  Search Screen - Advanced Search  Database - CINAHL Plus with Full Text | 69 |
| S63 | TI ( (LVRM or LVR) N10 (lung or volume or recruit* or man#euv*) ) OR AB ( (LVRM or LVR) N10 (lung or volume or recruit* or man#euv*) ) | Search modes - Find all my search terms | Interface - EBSCOhost Research Databases  Search Screen - Advanced Search  Database - CINAHL Plus with Full Text | 204 |
| S62 | TI ( (lung or alveolar) N1 recruit* N2 man#euv* ) OR AB ( (lung or alveolar) N1 recruit* N2 man#euv* ) | Search modes - Find all my search terms | Interface - EBSCOhost Research Databases  Search Screen - Advanced Search  Database - CINAHL Plus with Full Text | 150 |
| S61 | TI ( recruit* N2 ("lung volume" or alveolar) ) OR AB ( recruit* N2 ("lung volume" or alveolar) ) | Search modes - Find all my search terms | Interface - EBSCOhost Research Databases  Search Screen - Advanced Search  Database - CINAHL Plus with Full Text | 292 |
| S60 | TI ( respiratory muscle* N2 (aid* or support*) ) OR AB ( respiratory muscle* N2 (aid* or support*) ) | Search modes - Find all my search terms | Interface - EBSCOhost Research Databases  Search Screen - Advanced Search  Database - CINAHL Plus with Full Text | 124 |
| S59 | TI ( cough* N2 flow* N5 (improv* or increas* or enhanc* or expan* or exten*) ) OR AB ( cough* N2 flow* N5 (improv* or increas* or enhanc* or expan* or exten*) ) | Search modes - Find all my search terms | Interface - EBSCOhost Research Databases  Search Screen - Advanced Search  Database - CINAHL Plus with Full Text | 35 |
| S58 | TI ( (glossopharyn* or glosso-pharyn*) N2 (breath* or respirat*) ) OR AB ( (glossopharyn* or glosso-pharyn*) N2 (breath* or respirat*) ) | Search modes - Find all my search terms | Interface - EBSCOhost Research Databases  Search Screen - Advanced Search  Database - CINAHL Plus with Full Text | 30 |
| S57 | TI direct* N2 cough* OR AB direct* N2 cough* | Search modes - Find all my search terms | Interface - EBSCOhost Research Databases  Search Screen - Advanced Search  Database - CINAHL Plus with Full Text | 34 |
| S56 | TI ( airstack* or (air W0 stack*) ) OR AB ( airstack* or (air W0 stack*) ) | Search modes - Find all my search terms | Interface - EBSCOhost Research Databases  Search Screen - Advanced Search  Database - CINAHL Plus with Full Text | 21 |
| S55 | TI ( breathstack* or (breath W0 stack*) ) OR AB ( breathstack* or (breath W0 stack*) ) | Search modes - Find all my search terms | Interface - EBSCOhost Research Databases  Search Screen - Advanced Search  Database - CINAHL Plus with Full Text | 34 |
| S54 | TI "MI-E" OR AB "MI-E" | Search modes - Find all my search terms | Interface - EBSCOhost Research Databases  Search Screen - Advanced Search  Database - CINAHL Plus with Full Text | 53 |
| S53 | TI insufflat* N1 exsufflat* OR AB insufflat* N1 exsufflat* | Search modes - Find all my search terms | Interface - EBSCOhost Research Databases  Search Screen - Advanced Search  Database - CINAHL Plus with Full Text | 113 |
| S52 | TI ( "in-exsufflator" or "in-exsufflators" or "in-exsufflation" or "in-exsufflations" ) OR AB ( "in-exsufflator" or "in-exsufflators" or "in-exsufflation" or "in-exsufflations" ) | Search modes - Find all my search terms | Interface - EBSCOhost Research Databases  Search Screen - Advanced Search  Database - CINAHL Plus with Full Text | 161 |
| S51 | (MH "Insufflation") | Search modes - Find all my search terms | Interface - EBSCOhost Research Databases  Search Screen - Advanced Search  Database - CINAHL Plus with Full Text | 831 |
| S50 | (MH "Cough/RH") | Search modes - Find all my search terms | Interface - EBSCOhost Research Databases  Search Screen - Advanced Search  Database - CINAHL Plus with Full Text | 7 |
| S49 | TI cough* N2 augment* OR AB cough* N2 augment* | Search modes - Find all my search terms | Interface - EBSCOhost Research Databases  Search Screen - Advanced Search  Database - CINAHL Plus with Full Text | 40 |
| S48 | TI ( CoughAssist* or Pegaso* or Cofflator* or (Cof W0 flator*) or (cough W0 machine*) ) OR AB ( CoughAssist* or Pegaso* or Cofflator* or (Cof W0 flator*) or (cough W0 machine*) ) | Search modes - Find all my search terms | Interface - EBSCOhost Research Databases  Search Screen - Advanced Search  Database - CINAHL Plus with Full Text | 29 |
| S47 | TI cough* N2 assist* OR AB cough* N2 assist* | Search modes - Find all my search terms | Interface - EBSCOhost Research Databases  Search Screen - Advanced Search  Database - CINAHL Plus with Full Text | 155 |
| S46 | TI chest N3 compress* OR AB chest N3 compress* | Search modes - Find all my search terms | Interface - EBSCOhost Research Databases  Search Screen - Advanced Search  Database - CINAHL Plus with Full Text | 2,417 |
| S45 | TI ( CPR and (BVM or respirat* or resuscitat* or intubat*) ) OR AB ( CPR and (BVM or respirat* or resuscitat* or intubat*) ) | Search modes - Find all my search terms | Interface - EBSCOhost Research Databases  Search Screen - Advanced Search  Database - CINAHL Plus with Full Text | 3,667 |
| S44 | TI ( (cardiopulmonary or "cardio-pulmonary" or "mouth-to-mouth") N3 resuscitat* ) OR AB ( (cardiopulmonary or "cardio-pulmonary" or "mouth-to-mouth") N3 resuscitat* ) | Search modes - Find all my search terms | Interface - EBSCOhost Research Databases  Search Screen - Advanced Search  Database - CINAHL Plus with Full Text | 7,916 |
| S43 | (MH "Resuscitation, Cardiopulmonary+") | Search modes - Find all my search terms | Interface - EBSCOhost Research Databases  Search Screen - Advanced Search  Database - CINAHL Plus with Full Text | 16,104 |
| S42 | TI ( laryngotracheobronchoscop* or (laryngo W0 tracheobronchoscop*) or (laryngo W0 tracheo W0 bronchoscop*) or (laryngotracheo W0 bronchoscop*) or tracheobronchoscop* or (tracheo W0 bronchoscop*) ) OR AB ( laryngotracheobronchoscop* or (laryngo W0 tracheobronchoscop*) or (laryngo W0 tracheo W0 bronchoscop*) or (laryngotracheo W0 bronchoscop*) or tracheobronchoscop* or (tracheo W0 bronchoscop*) ) | Search modes - Find all my search terms | Interface - EBSCOhost Research Databases  Search Screen - Advanced Search  Database - CINAHL Plus with Full Text | 18 |
| S41 | TI bronch* N3 endoscop* OR AB bronch* N3 endoscop* | Search modes - Find all my search terms | Interface - EBSCOhost Research Databases  Search Screen - Advanced Search  Database - CINAHL Plus with Full Text | 125 |
| S40 | TI ( bronchoscop* or broncho W0 scop* ) OR AB ( bronchoscop* or broncho W0 scop* ) | Search modes - Find all my search terms | Interface - EBSCOhost Research Databases  Search Screen - Advanced Search  Database - CINAHL Plus with Full Text | 5,636 |
| S39 | (MH "Bronchoscopy") | Search modes - Find all my search terms | Interface - EBSCOhost Research Databases  Search Screen - Advanced Search  Database - CINAHL Plus with Full Text | 6,261 |
| S38 | TI Aerogen* OR AB Aerogen* | Search modes - Find all my search terms | Interface - EBSCOhost Research Databases  Search Screen - Advanced Search  Database - CINAHL Plus with Full Text | 201 |
| S37 | TI ( nebulize* or nebulise* or (inhal* N3 aerosol*) ) OR AB ( nebulize* or nebulise* or (inhal* N3 aerosol*) ) | Search modes - Find all my search terms | Interface - EBSCOhost Research Databases  Search Screen - Advanced Search  Database - CINAHL Plus with Full Text | 2,764 |
| S36 | (MH "Nebulizers and Vaporizers") | Search modes - Find all my search terms | Interface - EBSCOhost Research Databases  Search Screen - Advanced Search  Database - CINAHL Plus with Full Text | 5,421 |
| S35 | TI ( (inhalation N3 (challenge# or provocation)) and test* ) OR AB ( (inhalation N3 (challenge# or provocation)) and test* ) | Search modes - Find all my search terms | Interface - EBSCOhost Research Databases  Search Screen - Advanced Search  Database - CINAHL Plus with Full Text | 106 |
| S34 | TI BP W0 test* N10 bronch* OR AB BP W0 test* N10 bronch* | Search modes - Find all my search terms | Interface - EBSCOhost Research Databases  Search Screen - Advanced Search  Database - CINAHL Plus with Full Text | 0 |
| S33 | TI ( (bronchial* or endobronchial or "endo-bronchial") N3 (challenge# or provocation) ) OR AB ( (bronchial* or endobronchial or "endo-bronchial") N3 (challenge# or provocation) ) | Search modes - Find all my search terms | Interface - EBSCOhost Research Databases  Search Screen - Advanced Search  Database - CINAHL Plus with Full Text | 284 |
| S32 | (MH "Bronchial Provocation Tests") | Search modes - Find all my search terms | Interface - EBSCOhost Research Databases  Search Screen - Advanced Search  Database - CINAHL Plus with Full Text | 926 |
| S31 | TI FLO2max* OR AB FLO2max* | Search modes - Find all my search terms | Interface - EBSCOhost Research Databases  Search Screen - Advanced Search  Database - CINAHL Plus with Full Text | 1 |
| S30 | TI ( (air or gas or gases or gasses) N5 (atomi?er# or inhaler* or inhalator* or (inhalation W0 device#) or nebuli* or vapo#ri*) ) OR AB ( (air or gas or gases or gasses) N5 (atomi?er# or inhaler* or inhalator* or (inhalation W0 device#) or nebuli* or vapo#ri*) ) | Search modes - Find all my search terms | Interface - EBSCOhost Research Databases  Search Screen - Advanced Search  Database - CINAHL Plus with Full Text | 152 |
| S29 | ( oxygen* N5 (atomi?er# or inhaler* or inhalator* or (inhalation W0 device#) or nebuli* or vapo#ri*) ) OR ( oxygen* N5 (atomi?er# or inhaler* or inhalator* or (inhalation W0 device#) or nebuli* or vapo#ri*) ) | Search modes - Find all my search terms | Interface - EBSCOhost Research Databases  Search Screen - Advanced Search  Database - CINAHL Plus with Full Text | 160 |
| S28 | ( (MH "Oxygen") AND TI (atomi?er# or inhaler* or inhalator* or (inhalation W0 device#) or nebuli* or vapo#ri*) ) OR ( (MH "Oxygen") AND AB (atomi?er# or inhaler* or inhalator* or (inhalation W0 device#) or nebuli* or vapo#ri*) ) | Search modes - Find all my search terms | Interface - EBSCOhost Research Databases  Search Screen - Advanced Search  Database - CINAHL Plus with Full Text | 95 |
| S27 | TI ( "noninvasive PPV" or "non-invasive PPV" or NIPPV or NPPV ) OR AB ( "noninvasive PPV" or "non-invasive PPV" or NIPPV or NPPV ) | Search modes - Find all my search terms | Interface - EBSCOhost Research Databases  Search Screen - Advanced Search  Database - CINAHL Plus with Full Text | 423 |
| S26 | TI ( NIV N10 (noninvasive* or (non W0 nvasive*) or ventilat*) ) OR AB ( NIV N10 (noninvasive* or (non W0 nvasive*) or ventilat*) ) | Search modes - Find all my search terms | Interface - EBSCOhost Research Databases  Search Screen - Advanced Search  Database - CINAHL Plus with Full Text | 972 |
| S25 | TI ( (noninvasive* or (non W0 invasive*)) N3 ventilat* ) OR AB ( (noninvasive* or (non W0 invasive*)) N3 ventilat* ) | Search modes - Find all my search terms | Interface - EBSCOhost Research Databases  Search Screen - Advanced Search  Database - CINAHL Plus with Full Text | 4,023 |
| S24 | ( (MH "Noninvasive Procedures") and TI ventilat* ) OR ( (MH "Noninvasive Procedures") and AB ventilat* ) | Search modes - Find all my search terms | Interface - EBSCOhost Research Databases  Search Screen - Advanced Search  Database - CINAHL Plus with Full Text | 849 |
| S23 | TI ( APRV or BiPAP or BPAP or CPCP or nCPAP or CPPB or CPPV or IPPB or IPPV ) OR AB ( APRV or BiPAP or BPAP or CPCP or nCPAP or CPPB or CPPV or IPPB or IPPV ) | Search modes - Find all my search terms | Interface - EBSCOhost Research Databases  Search Screen - Advanced Search  Database - CINAHL Plus with Full Text | 968 |
| S22 | TI ( PEEP and (positive or expiratory or pressure) ) OR AB ( PEEP and (positive or expiratory or pressure) ) | Search modes - Find all my search terms | Interface - EBSCOhost Research Databases  Search Screen - Advanced Search  Database - CINAHL Plus with Full Text | 1,370 |
| S21 | TI "positive airway pressure" OR AB "positive airway pressure" | Search modes - Find all my search terms | Interface - EBSCOhost Research Databases  Search Screen - Advanced Search  Database - CINAHL Plus with Full Text | 4,479 |
| S20 | TI ( ((positive end-expiratory or (positive N2 pressure)) W0 (breathing or ventilat*)) ) OR AB ( ((positive end-expiratory or (positive N2 pressure)) W0 (breathing or ventilat*)) ) | Search modes - Find all my search terms | Interface - EBSCOhost Research Databases  Search Screen - Advanced Search  Database - CINAHL Plus with Full Text | 2,208 |
| S19 | (MH "Positive Pressure Ventilation+") | Search modes - Find all my search terms | Interface - EBSCOhost Research Databases  Search Screen - Advanced Search  Database - CINAHL Plus with Full Text | 12,144 |
| S18 | TI ( (ambu W0 bag#) or (bag W0 mask#) or (bag# N1 resuscitat*) or (manual* N1 resuscitat*) or ("self-inflating" W0 bag#) or (selfinflating W0 bag#) or "Revivator-Plus" ) OR AB ( (ambu W0 bag#) or (bag W0 mask#) or (bag# N1 resuscitat*) or (manual* N1 resuscitat*) or ("self-inflating" W0 bag#) or (selfinflating W0 bag#) or "Revivator-Plus" ) | Search modes - Find all my search terms | Interface - EBSCOhost Research Databases  Search Screen - Advanced Search  Database - CINAHL Plus with Full Text | 514 |
| S17 | TI ( BVM N10 (bag# or valve# or mask*) ) OR AB ( BVM N10 (bag# or valve# or mask*) ) | Search modes - Find all my search terms | Interface - EBSCOhost Research Databases  Search Screen - Advanced Search  Database - CINAHL Plus with Full Text | 72 |
| S16 | TI (bag W0 valve*) OR AB (bag W0 valve*) | Search modes - Find all my search terms | Interface - EBSCOhost Research Databases  Search Screen - Advanced Search  Database - CINAHL Plus with Full Text | 323 |
| S15 | TI ( autops* or ((postmortem or "post-mortem") W0 exam*) ) OR AB ( autops* or ((postmortem or "post-mortem") W0 exam*) ) | Search modes - Find all my search terms | Interface - EBSCOhost Research Databases  Search Screen - Advanced Search  Database - CINAHL Plus with Full Text | 9,929 |
| S14 | (MH "Autopsy") | Search modes - Find all my search terms | Interface - EBSCOhost Research Databases  Search Screen - Advanced Search  Database - CINAHL Plus with Full Text | 5,283 |
| S13 | TI ( (dent* N3 (handpiece* or (hand W0 piece*)) N5 (highspeed* or (high W0 speed*) or lowspeed* or (low W0 speed*)) ) OR AB ( (dent* N3 (handpiece* or (hand W0 piece*)) N5 (highspeed* or (high W0 speed*) or lowspeed* or (low W0 speed*)) ) | Search modes - Find all my search terms | Interface - EBSCOhost Research Databases  Search Screen - Advanced Search  Database - CINAHL Plus with Full Text | 19 |
| S12 | TI ( (air W0 polish*) or (air W0 abrasion*) or ("air turbine" W0 handpiece#) or ("air turbine hand" W0 piece#) ) OR AB ( (air W0 polish*) or (air W0 abrasion*) or ("air turbine" W0 handpiece#) or ("air turbine hand" W0 piece#) ) | Search modes - Find all my search terms | Interface - EBSCOhost Research Databases  Search Screen - Advanced Search  Database - CINAHL Plus with Full Text | 282 |
| S11 | TI ( (ultrasonic* or sonic*) N3 (scale* or scaling) ) OR AB ( (ultrasonic* or sonic*) N3 (scale* or scaling) ) | Search modes - Find all my search terms | Interface - EBSCOhost Research Databases  Search Screen - Advanced Search  Database - CINAHL Plus with Full Text | 244 |
| S10 | TI ( ((polish* or rotar* or finishing or handpiece* or hand piece*) N3 (device* or equipment* or instrument*)) and dent* ) OR AB ( ((polish* or rotar* or finishing or handpiece* or hand piece*) N3 (device* or equipment* or instrument*)) and dent* ) | Search modes - Find all my search terms | Interface - EBSCOhost Research Databases  Search Screen - Advanced Search  Database - CINAHL Plus with Full Text | 181 |
| S9 | TI ( (air rota or air rotar*) and dent* ) OR AB ( (air rota or air rotar*) and dent* ) | Search modes - Find all my search terms | Interface - EBSCOhost Research Databases  Search Screen - Advanced Search  Database - CINAHL Plus with Full Text | 6 |
| S8 | TI water N1 air# N2 syringe# OR AB water N1 air# N2 syringe# | Search modes - Find all my search terms | Interface - EBSCOhost Research Databases  Search Screen - Advanced Search  Database - CINAHL Plus with Full Text | 38 |
| S7 | TI "3-in-1" N2 syringe# OR AB "3-in-1" N2 syringe# | Search modes - Find all my search terms | Interface - EBSCOhost Research Databases  Search Screen - Advanced Search  Database - CINAHL Plus with Full Text | 3 |
| S6 | TI ( bioaerosol* or bio-aerosol* ) OR AB ( bioaerosol* or bio-aerosol* ) | Search modes - Find all my search terms | Interface - EBSCOhost Research Databases  Search Screen - Advanced Search  Database - CINAHL Plus with Full Text | 253 |
| S5 | TI ( aerosol* N5 (clinic* or dent* or medic* or therap* or treatment*) ) OR AB ( aerosol* N5 (clinic* or dent* or medic* or therap* or treatment*) ) | Search modes - Find all my search terms | Interface - EBSCOhost Research Databases  Search Screen - Advanced Search  Database - CINAHL Plus with Full Text | 902 |
| S4 | TI ( (AGP or AGPs or AGMP or AGMPs or AGDP or AGDPs) and aerosol* ) OR AB ( (AGP or AGPs or AGMP or AGMPs or AGDP or AGDPs) and aerosol* ) | Search modes - Find all my search terms | Interface - EBSCOhost Research Databases  Search Screen - Advanced Search  Database - CINAHL Plus with Full Text | 47 |
| S3 | TI ( aerosol* N3 (contact* or dispers* or expos* or generat* or procedure* or produc* or spread* or transmi*) ) OR AB ( aerosol* N3 (contact* or dispers* or expos* or generat* or procedure* or produc* or spread* or transmi*) ) | Search modes - Find all my search terms | Interface - EBSCOhost Research Databases  Search Screen - Advanced Search  Database - CINAHL Plus with Full Text | 1,377 |
| S2 | TI ( aerosoli?e* or aerosoli?ing ) OR AB ( aerosoli?e* or aerosoli?ing ) | Search modes - Find all my search terms | Interface - EBSCOhost Research Databases  Search Screen - Advanced Search  Database - CINAHL Plus with Full Text | 1,116 |
| S1 | (MH "Aerosols") | Search modes - Find all my search terms | Interface - EBSCOhost Research Databases  Search Screen - Advanced Search  Database - CINAHL Plus with Full Text | 3,494 |

**Aerosol Tranmission - HCWs**

Covid-Related Sources

2021 Sep 10

Cochrane

<https://covid-19.cochrane.org/>

2021 Sep 10

Healthcare Professional + Exposure to Covid-19

*47 studies (60 refs)*

Nursing Staff + Exposure to Covid-19

*1 studies (1 ref)*

Covid-END

<https://www.mcmasterforum.org/networks/covid-end/resources-to-support-decision-makers/Inventory-of-best-evidence-syntheses>

2021 Sep 10

Public Health Measures – CTRL+F - aerosol, worker, HCW – also scanned full list – results found

<https://www.mcmasterforum.org/find-evidence/covid-19-evidence/covid-19-evidence-from-hse-and-sse> - scanned full list – nothing new

L-OVE

<https://app.iloveevidence.com/>

PICO search: Health workers – Epidemiology – 1435 articles (21 broad synthesis, 121 systematic reviews, 1293 primary studies)

Note: Attempted to constructed searches using Advanced search interface with no success using various combinations of the following:

Aerosol* or AGP or AGPs or AGMP or AGMPs or AGDP or AGDPs

+

transmi* or spread* or expos* or contact* or risk* (*the above 2 combinations produce only 2 records* – *both opinion pieces*)

+

HCW or HCWs or "health care worker" or "healthcare worker" or "health care workers" or "healthcare workers" or "health worker" or "health workers"

"health care assistant" or "healthcare assistant" or "health assistant" or "health care assistants" or "healthcare assistants" or "health assistants" or "health care employee" or "healthcare employee" or "health employee" or "health care employees" or "healthcare employees" or "health employees" or "health care personnel" or "healthcare personnel" or "health personnel" or "health care professional" or "healthcare professional" or "health professional" or "health care professionals" or "healthcare professionals" or "health professionals" or "health care staff" or "healthcare staff" or "health staff"

"hospital assistant" or "hospital assistants" or "hospital employee" or "hospital employees" or "hospital personnel" or "hospital professional" or "hospital professionals" or "hospital staff"

"medical assistant" or "medical assistants" or "medical employee" or "medical employees" or "medical personnel" or "medical professional" or "medical professionals" or "medical staff"

"nursing assistant" or "nursing assistants" or "nursing employee" or "nursing employees" or "nursing personnel" or "nursing professional" or "nursing professionals" or "nursing staff"

"intensive care assistant" or "intensive care" or "intensive care employee" or "intensive care" or "intensive care personnel" or "intensive care professional" or "intensive care professionals" or "intensive care staff"

"critical care assistant" or "critical care assistants" or "critical care employee" or "critical care employees" or "critical care personnel" or "critical care professional" or "critical care professionals" or "critical care staff"

"ICU assistant" or "ICU assistants" or "ICU employee" or "ICU employees" or "ICU personnel" or "ICU professional" or "ICU professionals" or "ICU staff"

allergist* or anesthetist* or anaesthetist* or anesthethist* or anaesthethist* or anesthesiologist* or anaesthesiologist* or audiologist* or cardiologist* or dentist* or dermatologist* or doctor* or endocrinologist* or endodontist* or exodontist* or gastroenterologist* or gastro-enterologist* or practitioner" or geriatrician*

gynecologist* or gynaecologist* or hospitalist* or nephrologist* or neurologist* or nurse* or obstetrician* or oncologist* or ophthalmologist* or orthodontist* or osteopath or osteopaths or otolaryngologist*

paramedic* or pathologist* or pediatrician* or paediatrician* or periodontist* or physiatrist* or physician* or physiotherapist* or physio-therapist* or prosthodontist* or pulmonologist* or radiologist*

"respiratory technician" or "respiratory technicians" or respirologist* or rheumatologist* or surgeon* or therapist* or urologist*

"dental assistant" or "dental assistants" or "dental personnel" or "dental professional" or "dental professionals" or "dental staff" or hygienist* or "emr assistant" or "emr assistants" or "emr employee" or "emr employees" or "emr personnel" or "emr professional" or "emr professionals" or "emr staff" or "emr technician" or "emr technicians"

#1 AND #2 AND (#3 OR #4 OR #5 OR #6 OR #7 OR #8 OR #9 OR #10 OR #11 OR #12 OR #13 OR #14 OR #15)

UNCOVER

<https://www.ed.ac.uk/usher/uncover/register-of-reviews>

(*Use CTRL+F to browse full list*)

2021 Sep 10

Browsed by “aerosol”, “worker” – results found

ClinicalTrials.gov – Covid-19 resources

<https://clinicaltrials.gov/ct2/results?cond=COVID-19>

88 Studies found for: **HCW | COVID-19**

88 Studies found for: **HCWs | COVID-19**

634 Studies found for: **"health care worker" | COVID-19**

634 Studies found for: **"health care workers" | COVID-19**

**Synonyms:**

| health care worker | 634 studies | 6,300 studies |
| --- | --- | --- |
| health care professionals | 137 studies | 1,643 studies |
| Health Care Provider | 107 studies | 2,530 studies |
| health professionals | 73 studies | 1,247 studies |
| Health Personnel | 42 studies | 158 studies |
| health care personnel | 39 studies | 154 studies |
| Medical Personnel | 28 studies | 238 studies |
| Care personnel | 15 studies | 87 studies |
| Health Profession | 1 studies | 83 studies |

11 Studies found for: **"hospital worker" | COVID-19**

11 Studies found for: **"hospital workers" | COVID-19**

45 Studies found for: **"hospital personnel" | COVID-19**

45 Studies found for: **"hospital staff" | COVID-19**

165 Studies found for: **nurse | COVID-19**

165 Studies found for: **nurses | COVID-19**

No Studies found for: **"respiratory technician" | COVID-19**

No Studies found for: **"respiratory technicians" | COVID-19**

No Studies found for: **"respiratory technologist" | COVID-19**

No Studies found for: **"respiratory technologists" | COVID-19**

8 Studies found for: **"respiratory therapist" | COVID-19**

8 Studies found for: **"respiratory therapists" | COVID-19**

1 Study found for: **respirologist | COVID-19**

1 Study found for: **respirologists | COVID-**

72 Studies found for: **Aerosol | COVID-19**

72 Studies found for: **Aerosols | COVID-19**

WHO Covid-19 Database

<https://search.bvsalud.org/global-literature-on-novel-coronavirus-2019-ncov/>

2021 Sep 10

Infectious Disease Transmission, Patient-to-Professional – 795 records

MedRxiv

292 results for abstract or title "aerosol aerosols aerosolised aerosolized aerosolisation aerosolization bioaerosol bioaerosols AGP AGPs AGMP AGMPs AGDP AGDPs"

*picked selectively (73 records) from these results*
