# Supplementary material for: Risk of transmission of respiratory viruses during aerosol-generating medical procedures (AGMPs) revisited in the COVID-19 pandemic: a systematic review
Source: Antimicrob Resist Infect Control. 2022 Aug 11;11:102. doi: 10.1186/s13756-022-01133-8 (PMC9366810; doi:10.1186/s13756-022-01133-8)
Supplement: Supplementary file 2 — Additional file 2. List of AGMPs Considered in the Context of this Review. List of all the AGMPs considered in the context of this review, based on existing AGMP lists and by consensus from the Alberta Health Services’ AGMP guidance tool. [file 13756_2022_1133_MOESM2_ESM.docx]

Additional File 2: List of AGMPs Considered in the Context of this Review

1. Aerosol Generating Dental Instruments Such As: 3-In-1 Water/Air Syringe; Polishing Rotary Equipment, Rotary Instruments/Handpieces (All Speeds), Trimming Handpieces
2. Aerosolized Medication Administration
3. Autopsies
4. Bag Valve Masks, Includes Where Performed with ECT
5. Bi-level Positive Airway Pressure (e.g. BiPAP, CPAP) - Noninvasive Ventilation (NIV)
6. Blow by Oxygen Administration with Nebulizer
7. Bronchial Challenge (Pulmonary Function Studies) - Provocation with Nebulized Methacholine
8. Bronchoscopy
9. Cardiopulmonary Resuscitation (CPR) with Respiratory (BVM, Intubation)
10. Cough - Mechanical Cough Assist, Mechanical Insufflation Exsufflation (MIE)
11. Cutting/Shaving Frozen Sections (Cryostat)
12. Deep Tracheal Suctioning
13. High Frequency Oscillatory Ventilation
14. Infants/Young Children - Heated Humidified High Flow Oxygen (HHHFO)
15. Intubation & Related Procedures (e.g., Manual Ventilation, Open Endotracheal Suctioning, Extubation)
16. Intubation with Neonates
17. Lung Volume Recruitment Maneuvers (LVRM)
18. Manual resuscitator (Includes Bag Valve Mask, T-piece, Flow Inflating)
19. Mechanical Ventiliation, Unfiltered Air - Open System
20. Nasopharyngoscopy or Flexible Laryngoscopy Procedures - with High Pressure Irrigation or Suction
21. Neonatal: CPAP in Isolette
22. Open Respiratory or Airway Suctioning
23. Open Tracheostomy or Laryngectomy Tube, with Procedure or Manipulation Performed
24. Oxygen Devices with Total Delivered Flow Greater Than 30 LPM, Including Flush Flow (E.G. Venturi Devices, Or Combination of Interfaces)
25. Oxygen, Flush Flow, Regardless of Device Used
26. Oxygen, Heated Humidified High Flow (HHHFO) (E.G. AIRVO, Optiflow or Vapotherm)
27. Oxygen, Less Than & Including 15 LPM, Filtered Mask (E.G. FLO2max) - With Nebulizer
28. Oxygen / Medical Gas, Nebulized, Producing Visible Mist (Aerosols) (E.G. Cold, Medication, Small Volume, or Vibrating Mesh Nebulizers, I.E., Aerogen)
29. Oxygen, Non-Humidified (Dry), Total Flow Greater Than 30 LPM (Or Flush Flow), Any Single or Combination of Devices (E.G., Non-Rebreather Mask Over Nasal Cannula, Venturi Devices, Dry Cold Nebulizer)
30. Sputum Induction
31. Supraglottic Airways (i.e., LMA, King LT, iGel Used Pre-Hospital)
32. Tracheostomy Care
33. Transsphenoidal Surgery
34. Use of Propellant (e.g. Compressed Gas Delivery Method) Anesthetic Freezing Sprays on Mucosal Surfaces, such as Oral Lidocaine
35. Use of Propellant Freezing Sprays (Lab Use)
36. VQ Scan with Aerosol-Based Ventilation Agents
