# Supplementary material for: Risk of transmission of respiratory viruses during aerosol-generating medical procedures (AGMPs) revisited in the COVID-19 pandemic: a systematic review
Source: Antimicrob Resist Infect Control. 2022 Aug 11;11:102. doi: 10.1186/s13756-022-01133-8 (PMC9366810; doi:10.1186/s13756-022-01133-8)
Supplement: Supplementary file 3 — Additional file 3. List of Excluded Studies. List of excluded studies and main reason for exclusion. [file 13756_2022_1133_MOESM3_ESM.docx]

### **Additional File 3: List of Excluded Studies**

| **Study ID** | **Author, Year** | **Reason for Exclude** |
| --- | --- | --- |
| 54 | Abbas, 2021 | Not AGMP vs no AGMP |
| 79 | Zhang, 2021 | Not AGMP vs no AGMP |
| 173 | Kumar Goenka, 2021 | Not an AGMP |
| 180 | Bischoff, 2019 | Not an AGMP |
| 203 | Gao, 2020 | Not AGMP vs no AGMP |
| 242 | Jain, 2021 | Not AGMP vs no AGMP |
| 268 | Lawati, 2021 | Not an AGMP |
| 391 | Sun, 2020 | Not AGMP vs no AGMP |
| 480 | Yassi, 2021 | Not an AGMP |
| 492 | Nicholson, 2021 | Not comparative study design |
| 525 | Jones, 2021 | Not AGMP vs no AGMP |
| 528 | Westafer, 2021 | Not AGMP vs no AGMP |
| 762 | Pombo, 2020 | Not AGMP vs no AGMP |
| 770 | Vecchio, 2021 | Not AGMP vs no AGMP |
| 799 | Parotto, 2020 | Not AGMP vs no AGMP |
| 809 | Jungo, 2021 | Not AGMP vs no AGMP |
| 820 | Chang, 2021 | Not AGMP vs no AGMP |
| 855 | Basso, 2020 | Not AGMP vs no AGMP |
| 914 | Zhang, 2020 | Not AGMP vs no AGMP |
| 977 | Onoyama, 2020 | Not an AGMP |
| 1005 | Martinez, 2020 | Not AGMP vs no AGMP |
| 1031 | Foster, 2020 | Not comparative study design |
| 1047 | Boghdadly, 2020 | Not AGMP vs no AGMP |
| 1108 | Sowerby, 2020 | Not comparative study design |
| 1271 | Ashinyo, 2020 | Not acute respiratory infection |
| 1420 | Liu, 2020 | Not AGMP vs no AGMP |
| 1631 | McIntyre, 2017 | Not an AGMP |
| 1702 | Thompson, 2013 | Not AGMP vs no AGMP |
| 1847 | Caputo, 2006 | Not AGMP vs no AGMP |
| 1867 | Seto, 2013 | Not an AGMP |
| 2146 | Manjiyil, 2021 | Not an AGMP |
| 2158 | Gao, 2021 | Not AGMP vs no AGMP |
| 2249 | Rakislova, 2021 | Not AGMP vs no AGMP |
| 2282 | Schmitz, 2021 | Not AGMP vs no AGMP |
| 2533 | Shah, 2020 | Not AGMP vs no AGMP |
| 2545 | Inganson, 2021 | Not AGMP vs no AGMP |
| 2600 | Jin, 2020 | Not AGMP vs no AGMP |
| 2616 | Zhong, 2020 | Not an AGMP |
| 2771 | Cao, 2020 | Not AGMP vs no AGMP |
| 2867 | Gray, 2020 | Not comparative study design |
| 2895 | Laccourreye, 2020 | Not an AGMP |
| 2958 | Jabbar, 2020 | Not an AGMP |
| 3151 | Ver, 2020 | Duplicate |
| 3183 | Mattioli, 2020 | Not AGMP vs no AGMP |
| 3226 | Kori, 2020 | Not AGMP vs no AGMP |
| 3265 | Butt, 2016 | Not AGMP vs no AGMP |
| 3284 | Cummings, 2014 | Not AGMP vs no AGMP |
| 3683 | Mitchell, 2012 | Not AGMP vs no AGMP |
| 3769 | Faria, 2021 | Not AGMP vs no AGMP |
| 3805 | Montero, 2021 | Not AGMP vs no AGMP |
| 3807 | Boffetta, 2021 | Not AGMP vs no AGMP |
| 3829 | Morris, 2021 | Not AGMP vs no AGMP |
| 3837 | Kua, 2021 | Not AGMP vs no AGMP |
| 3854 | Mahto, 2021 | Not AGMP vs no AGMP |
| 3872 | Larribere, 2021 | Not AGMP vs no AGMP |
| 3931 | Mani, 2020 | Not AGMP vs no AGMP |
| 3941 | Batool, 2021 | Not AGMP vs no AGMP |
| 3957 | Samaranayake, 2021 | Not an AGMP |
| 3975 | Sims, 2020 | Not an AGMP |
| 3977 | Elfstrom, 2021 | Not AGMP vs no AGMP |
| 3982 | Dautzenberg, 2020 | Not AGMP vs no AGMP |
| 4017 | Bahrs, 2020 | Not AGMP vs no AGMP |
| 4030 | Sharma, 2021 | Not AGMP vs no AGMP |
| 4062 | Rebeiro, 2020 | Not AGMP vs no AGMP |
| 4093 | Ghosh, 2021 | Not AGMP vs no AGMP |
| 4108 | Dacosta, 2020 | Not AGMP vs no AGMP |
| 4117 | Handal, 2021 | Not AGMP vs no AGMP |
| 4156 | Datonye, 2021 | Not AGMP vs no AGMP |
| 4252 | Wang, 2020 | Not AGMP vs no AGMP |
| 4286 | Yavorovsky, 2020 | Not AGMP vs no AGMP |
| 4868 | Vimercati, 2021 | Not AGMP vs no AGMP |
| 4986 | Ochoa-Leite, 2021 | Not AGMP vs no AGMP |
| 5830 | Kavanagh, 2021 | Not AGMP vs no AGMP |
| 5897 | Schneider, 2020 | Not AGMP vs no AGMP |
| 6002 | Zheng, 2020 | Not AGMP vs no AGMP |
| 6054 | Wang, 2020 | Not AGMP vs no AGMP |
| 6058 | Lai, 2020 | Not AGMP vs no AGMP |
| 6154 | Canova, 2020 | Not comparative study design |
| HS12 | Periyasamy, 2020 | Not AGMP vs no AGMP |
| HS4 | Lotta, 2020 | Not AGMP vs no AGMP |
| HS6 | Loeb, 2009 | Not AGMP vs no AGMP |
| HS7 | MacIntyre, 2011 | Not AGMP vs no AGMP |
| HS8 | Radonovich, 2019 | Not AGMP vs no AGMP |
